# Effect of oral nintedanib vs placebo on epistaxis in hereditary hemorrhagic telangiectasia: the EPICURE multicenter randomized double-blind trial

**DOI:** 10.1007/s10456-024-09962-4

**Published:** 2024-12-24

**Authors:** Ruben Hermann, Vincent Grobost, Xavier Le-Guillou, Christian Lavigne, Antoine Parrot, Sophie Rivière, Julie Séguier, Anne-Emmanuelle Fargeton, Aurélie de-Montigny, Margaux Huot, Evelyne Decullier, Adeline Roux, Caroline Gervaise, César Cartier, Xavier Dufour, Margaux Grall, Frank Jegoux, Laurent Laccourreye, Justin Michel, Nicolas Saroul, Isabelle Wagner, Mallorie Kerjouan, Sophie Dupuis-Girod

**Affiliations:** 1 Service d’ORL, chirurgie cervico-faciale et d’audiophonologie, Hôpital Edouard Herriot, Hospices Civils de Lyon, 5 Place d’Arsonval, 69003 Lyon, France; 2 Service de Médecine Interne, CHU Estaing, Clermont-Ferrand University Hospital, Clermont-Ferrand, France; 3 Medical Genetics Department, University Hospital of Poitiers, 86000 Poitiers, France; 4 Department of Internal Medicine and Clinical Immunology, Angers University Hospital, Angers, France; 5 Assistance Publique-Hôpitaux de Paris, Service de Pneumologie et Centre de Compétence de la Maladie de Rendu Osler, Hôpital Tenon, 75020 Paris, France; 6 Service de Médecine Interne A, Centre Hospitalier Universitaire, Montpellier, France; 7 CHU de Montpellier, Hôpital St Eloi, Service de médecine Interne A and Centre d’Investigation Clinique, Inserm, CIC 1411, 34295 Montpellier Cedex, France; 8 Aix-Marseille Univ, APHM, Médecine Interne, Hôpital de la Timone, Marseille, France; 9 Service de Génétique et centre de reference de la maladie de Rendu-Osler, Hôpital Femme-Mère-Enfants, Hospices Civils de Lyon, Bron, France; 10 Service de Biostatistique et Bioinformatique des HCL, Lyon, France; 11 Pôle Santé Publique, Hospices Civils de Lyon, Lyon, France; 12 Service de pharmaceutique, Groupement Hospitalier Est, Hospices Civils de Lyon, Bron, France; 13 Service d’ORL Centre Hospitalier Universitaire, Montpellier, France; 14 Service de Chirurgie ORL, Centre Hospitalier Universitaire de Poitiers, Poitiers, France; 15 Service de Chirurgie ORL, Centre Hospitalier Universitaire Pontchaillou, Rennes, France; 16 Service d’ORL, CHU d’Angers, Angers cedex 9, France; 17 Department of Otorhinolaryngology-Head and Neck Surgery, Aix Marseille University, 36900APHM, IUSTI, La Conception University Hospital, Marseille, France; 18 Service d’ORL, Hôpital Gabriel Montpied, CHU Clermont Ferrand, Clermont-Ferrand, France; 19 Department of Otorhinolaryngology-Head and Neck Surgery, Sorbonne University, Tenon Hospital, AP-HP, Paris, France; 20 Service de Pneumologie, CHU Rennes, Rennes, France

**Keywords:** Hereditary hemorrhagic telangiectasia, Epistaxis, Anti-angiogenic, Nintedanib, Anemia, Tyrosine kinase inhibitors

## Abstract

Epistaxis greatly affects patients with hereditary hemorrhagic telangiectasia (HHT). Although few systemic treatment exist, nintedanib, is a good candidate thanks to its anti-angiogenic activity. Our main objective was to evaluate the efficacy of oral nintedanib on epistaxis duration in HHT patients with moderate to severe epistaxis. This multicenter phase 2 randomized, placebo-controlled, double-blind trial was conducted between June 2020 and February 2023. Inclusion criteria were being over 18 years old and having a confirmed HHT diagnosis with an epistaxis severity score greater than 4. Sixty patients were randomized to receive either nintedanib or placebo for 12 weeks with a 12 week follow-up. The primary endpoint was the proportion of patients achieving a reduction of at least 50% in mean monthly epistaxis duration comparing the 8 weeks before treatment to the last 8 weeks of treatment. Main secondary outcomes included monthly duration and frequency of epistaxis and hemoglobin levels. Of the 60 randomized patients, 56 completed the trial. Thirteen patients (43%) in the nintedanib group *vs* 8 (27%) in the placebo group met the primary endpoint (*p* = 0.28). We observed a significant decrease in median epistaxis (57% vs 27%, *p* = 0.013) and a significant increase in median hemoglobin levels (+ 18 vs − 1 g/L, *p* = 0.02) in the nintedanib vs the placebo group. Although we did not achieve our primary outcome, we observed a significant reduction in epistaxis duration and a significant increase in hemoglobin levels in patients treated with nintedanib. This supports the efficacy of nintedanib, and further studies are needed.

**Supplementary Information:**

The online version contains supplementary material available at 10.1007/s10456-024-09962-4.

## Introduction

Epistaxis is the most common manifestation of hereditary hemorrhagic telangiectasia (HHT), affecting over 90% of patients [1]. It is the main expression of the nasal telangiectases, which result from impaired angiogenesis in HHT. In most cases, HHT is associated with heterozygous mutations of the *ACVRL1* or *ENG* genes, which respectively encode a bone morphogenetic protein receptor activin receptor-like kinase 1 and a co-receptor named endoglin. In addition, mutations in the *SMAD4* gene, which are responsible for juvenile polyposis/HHT overlap syndrome, have been described. All the products of these genes regulate the same bone morphogenetic protein 9/10 (BMP) signaling pathway and vascular quiescence [2].

In addition to reducing quality of life [3, 4], these recurrent and often severe nose bleeds can lead to iron deficiency as well as life threatening anemia [5]. To date, treatment recommendations include the use of moisturizing topical therapies, tranexamic acid and ablative therapy [6]. Although these options are important aspects of managing HHT, they have not been shown to decrease epistaxis in the medium to long term. Furthermore, ablative therapy can lead to perforation of the nasal septum, which in turn increases the frequency and severity of epistaxis. In cases of insufficient response to these options, systemic anti-angiogenic agents such as bevacizumab can be used. This treatment has shown promising results [7], but its use is limited to severe epistaxis because of to the administration route, price and absence of market authorization. Exploring other anti-angiogenic drugs for use in HHT is thus of critical importance.

Tyrosine kinase inhibitors (TKI) are a class of anti-angiogenic drugs that can be taken orally and could potentially be used to treat epistaxis in HHT. Some TKI such as sorafenib and pazopanib have shown interesting results in the development of adult-onset arteriovenous malformations in a murine model of HHT [8]. A previous study has also shown promising results for pazopanib in patients but the trial was discontinued due to external factors [9]. Another interesting candidate is nintedanib, a TKI that inhibits growth factor receptors involved in angiogenesis such as platelet-derived growth factor receptor (PDGFR), fibroblast growth factor receptor (FGFR) and vascular endothelial growth factor receptor (VEGFR). It has been shown to prevent vascular pathologies and reduce gastro-intestinal bleeding in a murine model of HHT when added to sirolimus [10]. Additionally, a case report of an HHT patient treated with nintedanib for pulmonary fibrosis has been published recently showing a decrease in the epistaxis severity score (ESS) from 5.5/10 to 0.5/10 [11]. Its safety is well documented as it has been used for many years for the treatment of idiopathic pulmonary fibrosis, systemic sclerosis associated interstitial lung disease and progressive pulmonary fibrosis [12, 13]. The most common adverse reactions are gastro-intestinal, such as diarrhea, vomiting and abdominal pain. We hypothesize that nintedanib, acting by indirect inhibition of the VEGFR should make possible reductions in epistaxis in HHT patients. However, to date, evidence is lacking, and placebo-controlled trials are needed to validate this hypothesis.

The main objective of this study was to evaluate the efficacy on epistaxis duration of an oral nintedanib treatment (2 × 150 mg/day for 12 weeks) versus placebo, at the end of the treatment period, in patients with HHT complicated by moderate to severe epistaxis.

## Methods

### Study design (Fig. 1)

**Fig. 1:**
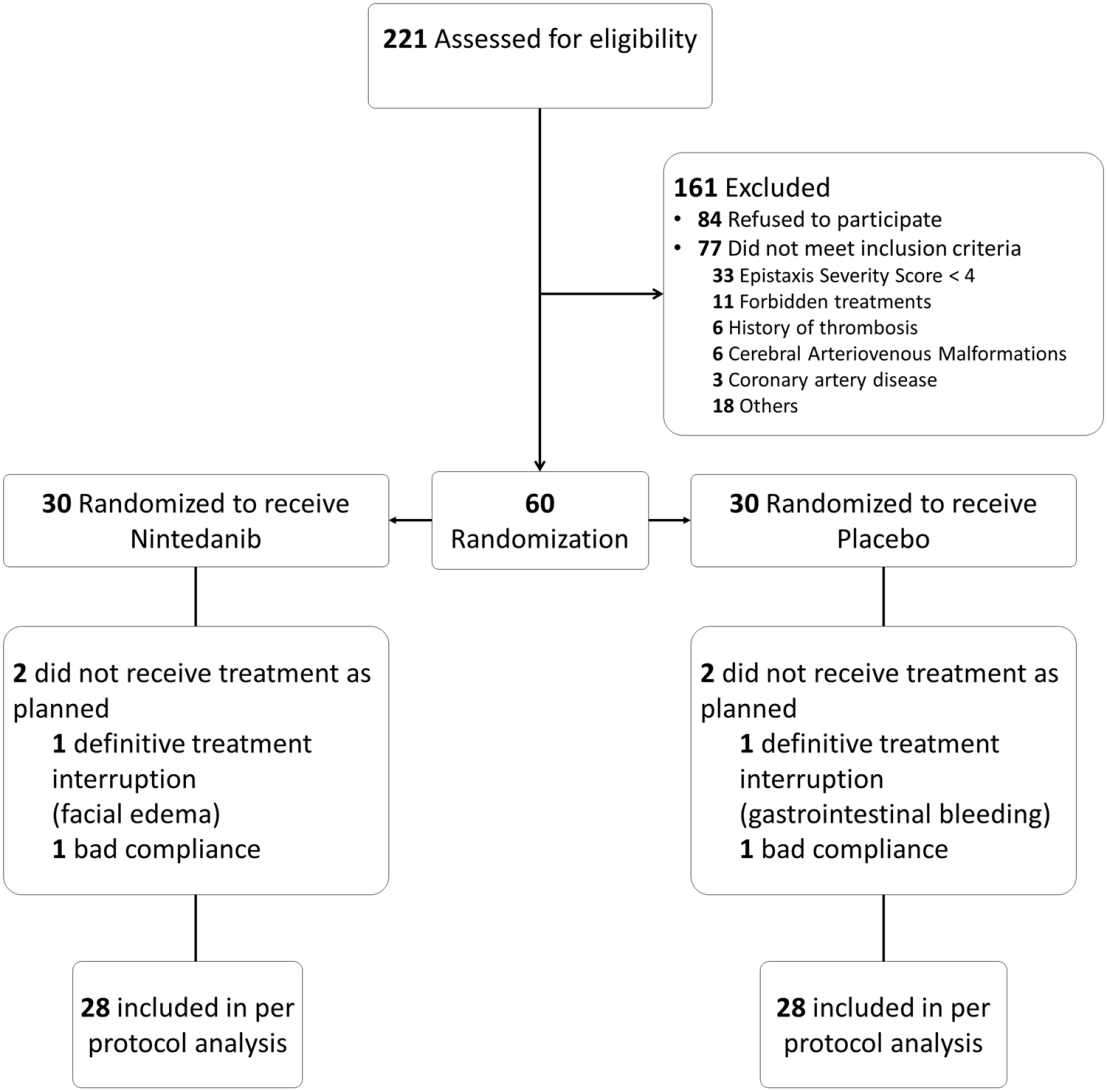
Flow diagram

This phase II study was a national, prospective, multicenter trial, comparing nintedanib to placebo in a parallel-group, randomized design with a 1:1 ratio. It was conducted in a double-blind setting, with both the treatment duration and follow-up period set at 12 weeks.

It was approved by the research ethics committee and authorized by the French medical products agency (ANSM). All patients gave their oral and written consent in accordance with national regulations. The study was registered in a public trials registry (clinicaltrials.gov identifier NCT03954782).

### Participants

This study enrolled patients aged 18 years and older, with clinically confirmed HHT and moderate to severe epistaxis defined by an epistaxis severity score (ESS) greater than 4.

Patients in the HHT network were informed during a standard follow-up consultation in an HHT center. All French HHT centers were involved in patient selection, but treatments were centralized in ten hospitals across the country.

Exclusion criteria included the presence of non-treated pulmonary AVMs, hemoptysis, hematuria, overt gastro-intestinal bleeding or ulcers within 12 months, cerebral AVM on MRI, having a liver disease or renal failure or active infection, known coronary artery disease or predisposition to thrombosis, use of certain medications (e.g., anticoagulant or antiplatelet therapies, other anti-angiogenic treatments, P-glycoprotein substrates/inducers/inhibitor treatments), presence of unhealed wounds or recent surgery; having QTc prolongation.

### Randomization and blinding

The randomization process was centralized. A unique list for all centers was generated using SAS® by the Pôle de Santé Publique at the Hospices Civils de Lyon—clinical research unit, using random block sizes of 4 and 6. Patients included were randomly assigned to one of two treatment groups using the IWRS (interactive web response system) based on this list. The software ENNOV clinical version 7.1 (clinsight) was used for the data-management.

### Interventions

In the nintedanib group, patients received 150 mg of nintedanib twice daily, administered orally approximately 12 h apart every day for 12 weeks. The nintedanib was manufactured by the company Boehringer Ingelheim and commercialized as OFEV® 100 mg and 150 mg containing respectively 100 mg and 150 mg of nintedanib as esilate. The comparative treatment was a placebo provided by the same company as soft gelatin capsules (100 mg and 150 mg) containing a suspension of titanium dioxide as the drug substance substitute, and identical in appearance to the nintedanib. If a dose was missed, administration resumed at the next scheduled time at the recommended dose and no additional dose was taken so as not to exceed the recommended maximum daily dose of 300 mg. In case of adverse reactions, the treatment could be temporarily interrupted or adjusted to 200 mg (100 mg twice daily). If adverse reactions persisted after the dose reduction, the treatment was discontinued.

### Outcomes

#### Primary outcome

The primary endpoint was the proportion of patients achieving a reduction of at least 50% in mean monthly duration of epistaxis in the last 8 weeks of treatment (P2) as compared to the 8 weeks before treatment (P1). This criterion was assessed by monitoring epistaxis grids filled in daily by the patients and collected at each visit or filled in online. These grids contained the number of episodes per day and their duration. The mean monthly duration of epistaxis on each reporting period was computed over the last 56 days or less (corresponding to 8 weeks) and normalized/reduced to 28 days, considering that 1 month is equal to 4 weeks, thanks to the following formula: $28* \frac{{\sum }_{1}^{max56}epistaxis\,daily\,duration}{total\,number\,of\,days\,observed}$

#### Secondary outcomes

Epistaxis was also assessed with regard to.

- Epistaxis monthly duration as a continuous variable and epistaxis frequency before, during and after treatment.
- Reduction of at least 50% in the mean monthly duration of epistaxis during the last 8 weeks of follow-up (P3) as compared to the 8 weeks before treatment (P1).
- Epistaxis severity score (ESS) at the inclusion visit, end of treatment, and end of follow-up.

Other clinical criteria were also assessed, such as quality of life using SF-36 questionnaires (filled out by the patients at the inclusion visit, at the end of the treatment and follow-up periods), and number of red blood cell (RBC) units transfused and iron infusions (for the 8 weeks before treatment, during the last 8 weeks of treatment and follow-up periods). National recommendations were used regarding indications for RBC unit transfusions. No specific protocol was implemented for iron supplementation during the study.

Biological criteria such as hemoglobin and ferritin levels were also measured at inclusion, and at the end of the treatment and follow-up periods. The different criteria were assessed during the six on site visits.

Safety criteria: all adverse events (AE) and severe AE were collected throughout the study and coded using the medical dictionary for regulatory activities (MEDDRA) and graded according to the common terminology criteria for AE (CTCAE) classification. A safety committee reviewed all the adverse events collected and their relationship to the study treatment.

### Sample size calculation

We hypothesized that 60% of patients would show improvement in the treatment group against 15% in the placebo group. Twenty-seven patients included in each group would make it possible to attain a power of 90% according to a Fisher’s exact test, leading to 54 patients overall.

Considering early withdrawal and patients who may be lost to follow-up, we decided to include 30 patients in each group, leading to a total of 60 patients.

### Statistical methods

Baseline characteristics were summarized as number of patients (%) for categorical variables and as median (Q1–Q3) and mean (SD) for continuous variables. All analyses were carried out on the ITT population, only the main analysis on the primary endpoint was also performed on the PP population.

Between-group differences were tested using the Mann–Whitney test for quantitative outcomes and the Fisher test for qualitative outcomes (both non-parametric tests). A two-sided *p*-value of less than 0.05 was considered statistically significant. No adjustment for multiple testing was performed. Two-sided 95% confidence intervals were used.

For patients with fewer than 14 days (inclusive) missing on epistaxis grids, the mean monthly duration was computed on the data observed (from the 8-week, 56-day period evaluated). For patients with more than 14 days missing on the grids in P3, the result for categorical data was imputed as a failure in the nintedanib group and success in the placebo group.

All statistical analyses were performed using R software version 4.1.1.

## Results

### Patients baseline characteristics (*n* = 60)

Sixty patients were randomized at eight out of the ten participating centers from June 2020 to September 2022—30 in the nintedanib group and 30 in the placebo group, constituting the ITT population. The baseline characteristics are summarized in Table 1.

**Table 1:** Baseline characteristics—ITT population

		Nintedanib *N* = 30	Placebo *N* = 30
Sex	Female *n* (%)	15 (50)	16 (53)
Age (years)	Median [Q1–Q3] Mean (SD)	59.0 [50.3–62.0] 57.1 (12.1)	57.0 [53.0–61.0] 56.1 (8.3)
Gene involved	Alk1 *n* (%) Eng *n* (%) Not identified *n* (%)	24 (80) 4 (13.3) 2 (6.7)	27 (90) 3 (10) 0 (0)
Nasal surgery	*N* (%)	10 (33.3)	10 (33.3)
Septal perforation	*N* (%)	3 (11.5)	2 (7.1)
Epistaxis monthly duration (min)	Median [Q1–Q3] Mean (SD)	264.0 [138.9–436.0] 314.0 (227.4)	258.4 [147.8–350.1] 274.4 (154.1)
Epistaxis monthly frequency	Median [Q1–Q3] Mean (SD)	40.3 [18.5–55.9] 39.6 (24.8)	29.3 [16.9–42.7] 32.75 (19.7)
Epistaxis severity score	Median [Q1–Q3] Mean (SD)	5.3 [5.1–6.0] 5.5 (0.7)	5.4 [5.1–6.1] 5.6 (1.0)
Hemoglobin (g/L)	Median [Q1–Q3] Mean (SD)	117.0 [104.3–139.0] 119.6 (22.3)	127.5 [100.8–140.8] 124.0 (23.4)

Four patients were excluded from the per protocol analysis: two because of poor compliance (one from each group) and two because of a permanent treatment discontinuation during the study (one from each group).

### Primary outcome

In the ITT population, there was no statistically significant difference between the two groups regarding the proportion of patients who experienced a reduction of at least 50% in the mean monthly duration of epistaxis during the last 8 weeks of treatment (P2) as compared to the 8 weeks before treatment (P1). For the nintedanib and the placebo groups respectively this represents 13 patients (43.3%) and 8 patients (26.7%) (*p* = 0.28). Comparable results were observed for the per-protocol analysis (with 12 patients (42.9%) in the nintedanib group *vs* 8 patients (28.6%) in the placebo group, *p* = 0.40).

### Secondary outcomes

#### Epistaxis

**Fig. 2:**
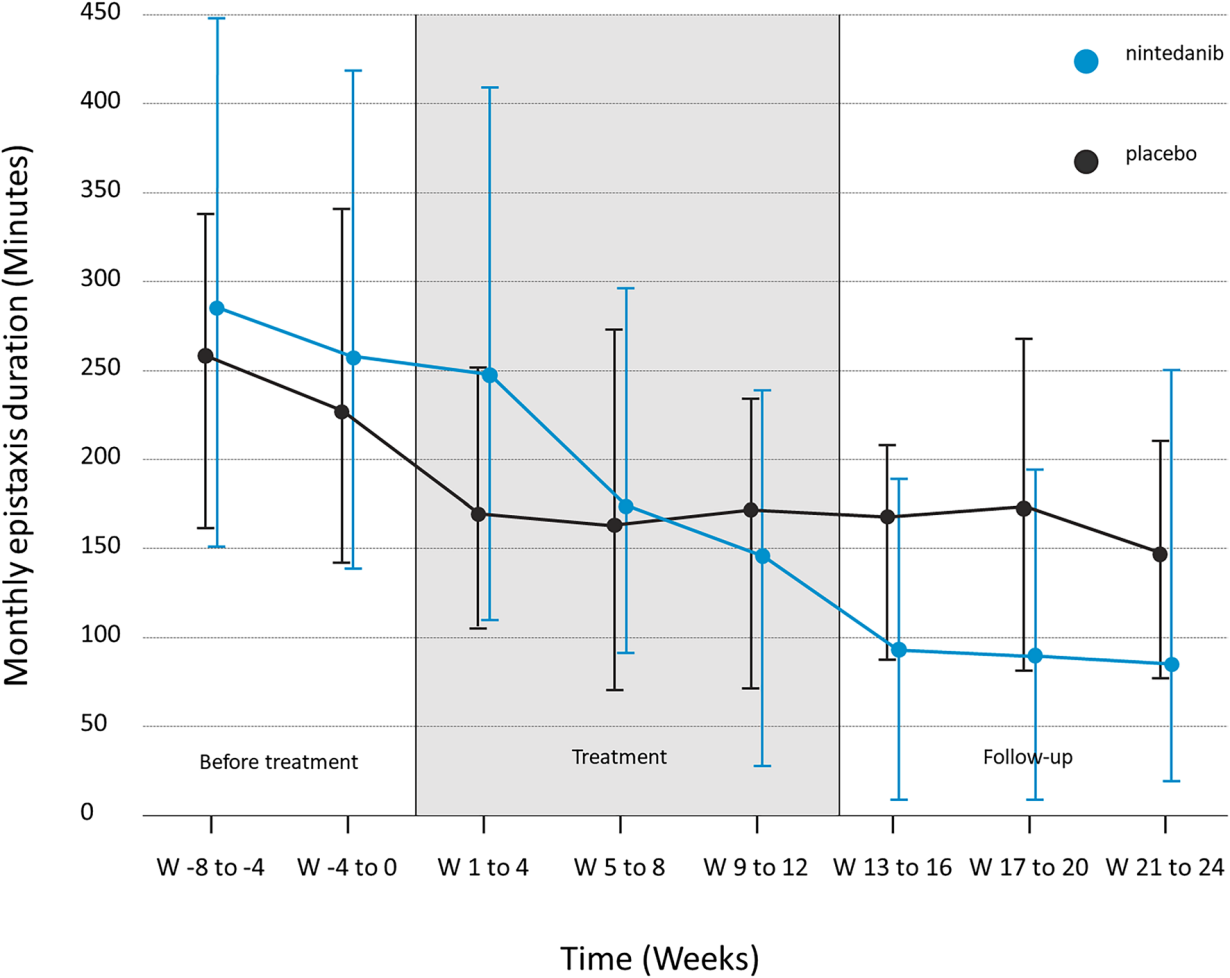
Evolution of monthly epistaxis duration before during and after treatment. Median, 1st and 3rd quartile of epistaxis monthly duration before, during and after treatment in the nintedanib (blue) and placebo (dark grey) groups considering that 1 month is equal to 4 weeks i.e. 28 days. ITT population

We observed a significant decrease in the median duration of epistaxis between P1 and P3, with a 57% reduction the nintedanib group compared to 24% in the placebo group (*p* = 0.013). Similarly, there was a decrease in the monthly frequency of epistaxis of 35% in the nintedanib group compared to 12% in the placebo group (*p* = 0.018). Detailed results regarding the monthly duration and frequency of epistaxis for the nintedanib and placebo groups respectively are shown in Table 2. The evolution in monthly duration of epistaxis during the study is illustrated in Fig. 2.

**Table 2:** Secondary outcomes of monthly epistaxis duration and frequency, hemoglobin, and ferritin levels in patients under nintedanib versus placebo—ITT population

			Nintedanib *N* = 30	Placebo *N* = 30	Significant (*p*)	Relative changes between
Epistaxis monthly duration (minutes)	Median [Q1–Q3] Mean (SD)	P1	264 [138–436] 314 (227)	258 [147–350] 274 (154)	NS	P1 and P2
		P2	162 [73–246] 206 (204)	170 [87–252] 180 (127)	**0.013**	P1 and P3
		P3	82 [13–219] 155 (214)	156 [100–201] 172 (105)	**0.002**	P2 and P3
Epistaxis monthly frequency	Median [Q1–Q3] Mean (SD)	P1	40 [19–56] 40 (25)	29 [17–43] 33 [20]	NS	P1 and P2
		P2	18 [9–47] 30 (29)	20 [14–31] 26 (20)	**0.018**	P1 and P3
		P3	11 [6–45] 26 (27)	19 [14–37] 27 (20)	**0.003**	P2 and P3
Hemoglobin level (g/L)	Median [Q1–Q3] Mean (SD)	End of P1	117 [104–139] 120 (22)	127 [101–141] 124 (23)	**0.019**	End of P1 and End of P2
		End of P2	135 [118–143] 131 (20)	126 [105–136] 122 (20)	NS	End of P1 and End of P3
		End of P3	135 [110–145] 128 (23)	129 [118–137] 126 (20)	NS	End of P2 and End of P3
Ferritin level (µ/L)	Median [Q1–Q3] Mean (SD)	End of P1	36 [9–61] 70 (113)	27 [19–44] 42 ( 55)	NS	End of P1 and End of P2
		End of P2	50 [30–93] 101 (139)	37 [24–60] 80 (157)	NS	End of P1 and End of P3
		End of P3	59 [32–185] 140 (174)	32 [24–55] 56 (82)	NS	End of P2 and End of P3

Significant values are highlighted in bold

P1: 8 weeks before treatment; P2: last 8 weeks of treatment; P3: last 8 weeks of follow-up. The 2 two rightmost columns show the significance of the relative changes between different periods

*NS* not significant

There was no statistically significant difference in the number of patients experiencing a reduction of at least 50% in monthly epistaxis duration between the two groups when comparing Period 1 to Period 3 (17 patients (57%) in the nintedanib group and 11 (37%) in the placebo group, *p* = 0.19) or Period 2 to Period 3 (11 patients (37%) in the nintedanib group and 4 (13%) in the placebo group, *p* = 0.07).

There were no statistically significant changes in the epistaxis severity score between the nintedanib and placebo groups at any timepoint during the study. Median ESS [Q1–Q3] for the nintedanib and placebo groups were respectively 5.29 [5.07–5.98] vs 5.38 [5.09–6.08] at the end of P1, 3.38 [2.53–5.12] vs 4.30 [2.96–5.84] at the end of P2, and 3.38 [2.18–5.22] vs 4.06 [3.33–5.33] at the end of P3.

### Quality of life

No statistically significant differences in any of the 8 subscores nor the 2 summary scores of the SF-36 questionnaire between the nintedanib and placebo groups were observed across the periods assessed (Supplementary data).

### Red blood cell units transfused and iron

During P1, P2, and P3, the number of patients with a least one RBC unit transfusion was similar for the nintedanib and placebo groups (2, 1, 2 vs 2, 2, 3 respectively). Additionally, the number of injections during the same periods were 9, 6, 10 and 10, 9, 8 for the nintedanib and placebo groups, respectively.

There was a statistically significant increase in median hemoglobin levels in the nintedanib group from 117 to 135 versus from 127 to 126 g/L in the placebo group, between inclusion and the end of treatment (+ 7% vs − 1%, *p* = 0.02). There was no statistically significant difference in ferritin levels between the two groups across the same periods assessed. Detailed results regarding hemoglobin and ferritin levels are shown in Table 2.

### Adverse effects

Details regarding adverse events are shown in Table 3.

**Table 3:** Adverse events—IIT population

		Nintedanib *N* = 30	Placebo *N* = 30	*p*
Patients with at least one AE	*N* (%)	26 (87%)	19 (63%)	0.072
Number of AE	Events (*n*)	127	63	
Patients with at least one AE related to treatment	*N* (%)	25 (83%)	10 (33%)	< 0.01
Number of AE related to treatment	Events (*n*)	85	23	
Patients with at least 1 serious AE	*N* (%)	2 (7%)	4 (13%)	0.671
Number of serious AE	Events (*n*)	3	7	

Of the 60 patients included and treated, 45 experienced adverse effects (AEs) totaling 190 events, including 10 serious AEs in 6 patients (3 in the nintedanib group and 7 in the placebo group).

Of all the AEs, 108 were considered to be related to the trial treatment (85 in the nintedanib group versus 23 in the placebo group, in 25 patients (83%) vs 10 patients (33%) respectively). The most common AEs observed in the nintedanib group were diarrhea (20 AEs in 11 patients), nausea (9 AEs in 9 patients), abdominal pain (8 AEs in 7 patients) and headaches (8 AEs in 6 patients).

Two adverse events led to a definitive treatment interruption. One because of life-threatening gastrointestinal bleeding in the placebo group, and one because of facial edema in the nintedanib group.

Three patients from the nintedanib group had a dose reduction as per protocol design from 150 mg twice daily to 100 mg twice daily due to vomiting, diarrhea, and skin rash. Three other patients from the nintedanib group had a temporary treatment interruption of less than 7 days due to diarrhea, nausea, or abdominal pain. Two patients had a temporary treatment interruption of more than 7 days. One due to an injury in the placebo group and the other due to persistent diarrhea in the nintedanib group.

## Discussion

The EPICURE trial is the first structured, randomized, clinical trial of nintedanib in HHT patients. Nintedanib did not lead to a reduction of at least 50% in epistaxis duration *vs* placebo; thus, our primary outcome was not met. However, the results did show a continuous decrease in epistaxis duration in HHT patients receiving nintedanib during treatment and the months following the end of the treatment. On the contrary, epistaxis duration in the placebo group improved before treatment but was stable during and after treatment. Thus, the high variability in epistaxis duration over time in a same patient and the improvement in epistaxis duration in the placebo group before any treatment makes it very difficult to use this symptom as a criterion for judgment in HHT, as already reported in other studies [14, 15]. However, these results are encouraging and supported by the significant improvement in hemoglobin levels during treatment in the nintedanib group.

Furthermore, these results concord with other data on the use of TKI in HHT. In the literature, a prospective open label study on 7 HHT patients [9] and a retrospective study evaluating 13 HHT patients with RBC transfusion [16], highlighted the potential improvement in bleeding thanks to pazopanib in HHT patients. Pazopanib is mainly used in the treatment of advanced renal cell carcinoma and soft tissue carcinoma [17]. Pazopanib and nintedanib could have similar efficacy in HHT, as both are multi-tyrosine kinase inhibitors and inhibit a number of growth factors, such as vascular endothelial growth factor receptors (VEGFR-1, -2, and -3), platelet-derived growth factor receptors (PDGFR-ɑ and -β), stem cell factor receptor (c-Kit), and (FGFR-1 and -3). Two phase II studies evaluating pazopanib on bleeding in HHT are ongoing in North America (NCT02204371 and NCT03850964).

While treatment-related adverse events were frequent in the nintedanib group, only two patients in this group experienced severe adverse events, leading to treatment interruption in one case. The three patients who required a treatment dose reduction from 300 to 200 mg did not need to discontinue treatment, as the dose reduction decreased the intensity of the adverse events. A previous study involving over 600 patients treated with nintedanib for idiopathic pulmonary fibrosis has already demonstrated manageable safety and tolerability, as well as a low impact of dose reduction on the disease [18]. Furthermore, contrary to pazopanib, nintedanib does not seem to induce high blood pressure. To reduce potential adverse effects, periodic treatment could be interesting as nintedanib seems to have a persistent effect on bleedings. Quality of life assessed using the SF36 questionnaire did not improve. Although the SF-36 questionnaire is a widely used instrument for measuring health-related quality of life, it is a generic tool not specific to any particular disease. As such, it might not have been sensitive enough to detect changes in quality of life in the context of this study. We recently validated a dedicated tool which unfortunately was not accessible when we started this study [19, 20]. In future clinical trials, the SF-36 questionnaire will be replaced by the QoL-HHT questionnaire, which provides a more precise assessment of quality of life in patients with HHT. Similarly, the epistaxis severity score (ESS) was evaluated. This tool is used to assess the severity of nosebleeds in individuals with HHT [21]. This score can help healthcare professionals to assess the severity of epistaxis and monitor changes over time, but is subjective and a recent evaluation of this score demonstrated low internal consistency [22], which is in accordance with our results.

There is strong evidence over the past 15 years regarding the efficacy of bevacizumab in patients with HHT, particularly those with severe liver involvement and significant bleeding [7, 23, 24]. As this is the first randomized clinical trial on nintedanib in HHT, a direct comparison between the two treatments is not yet feasible. Given the established scientific evidence and the fewer side effects associated with bevacizumab, it should remain the first-line anti-angiogenic treatment for HHT. However, nintedanib may serve as a valuable alternative for certain patient populations—specifically those for whom bevacizumab is contraindicated, poorly tolerated, or ineffective. This study has many limitations. As we noticed in past clinical trials in HHT, there is a lack of standardized outcome measures in this disease, and developing appropriate endpoints tailored to HHT is challenging. Epistaxis in HHT is highly variable from one patient to another, but more importantly within a same patient, and epistaxis is very difficult to measure. Other criteria, such as the epistaxis severity score, are highly subjective and can be significantly influenced by the study, particularly if drug side effects compromise double-blinding. Variability in symptoms, disease progression, and severity among patients, is particularly crucial in rare diseases due to the small population. This heterogeneity can complicate the design of clinical trials, as it may be challenging to define homogeneous patient populations for study inclusion criteria. This makes it difficult to recruit enough participants for traditional clinical trials, leading to slower recruitment timelines and potentially limiting the statistical power of the study. Although we excluded patients with overt gastrointestinal bleeding, no systematic gastroscopy or video capsule endoscopy were performed before inclusion to check for occult gastrointestinal bleeding. Therefore, it is not possible to determine whether the increase in hemoglobin is solely due to a reduction in nosebleeds or if occult gastrointestinal bleeding was also reduced. In conclusion, and although we did not achieve our primary outcome, we observed a significant reduction in epistaxis duration and a significant increase in hemoglobin level in HHT patients treated with nintedanib vs placebo. This result supports the efficacy of nintedanib and further studies are needed.

## Supplementary Information

Below is the link to the electronic supplementary material.

Supplementary file1 (PDF 146 KB)

Supplementary file2 (PDF 6045 KB)

Supplementary file3 (PDF 1728 KB)

Supplementary file4 (PDF 1059 KB)

## Data Availability

The following documents have been made available by the authors for reviewers at submission: study protocol, protocol amendments, and statistical analysis plan. Supplementary data include detailed results on quality of life. Additional data can be made available upon email request to the corresponding author.
