# Supplementary material for: Effect of oral nintedanib vs placebo on epistaxis in hereditary hemorrhagic telangiectasia: the EPICURE multicenter randomized double-blind trial
Source: Angiogenesis. 2024 Dec 24;28(1):9. doi: 10.1007/s10456-024-09962-4 (PMC11668894; doi:10.1007/s10456-024-09962-4)
Supplement: Supplementary file 2 — Supplementary file2 (PDF 6045 KB) [file 10456_2024_9962_MOESM2_ESM.pdf]

Efficacy of Nintedanib per os as a treatment for epistaxis in HHT disease
A national, randomized, multicenter phase II study

EPICURE

Cat. 1 protocol involving human individuals and concerning a drug

Version 04 dated 24/03/2022

Sponsor: Hospices Civils de Lyon
BP 2251
3 quai des Célestins,
69229 LYON Cedex 02

Principal investigator :
Docteur Sophie DUPUIS-GIROD
Department of genetics
HFME A1
59 bd Pinel
69677 BRON cedex
Phone: 04 27 85 65 25 Fax: 04 27 85 67 98
Email: sophie.dupuis-girod@chu-lyon.fr

Sponsor code: 69HCL19_0003
Clinicaltrials.gov registration number: *NCT03954782*
Favorable opinion from EC Ouest I on: 05/11/2019
ANSM authorization on: 31/10/2019

SUMMARY

**TITLE**	EPICURE<br>Efficacy of nintedanib per os as a treatment for epistaxis in HHT disease. A national, randomized, multicenter phase II study
**SPONSOR**	Hospices Civils de Lyon<br>BP 2251 - 3 quai des Célestins - 69229 LYON Cedex 02
**PRINCIPAL INVESTIGATOR**	Dr Sophie DUPUIS-GIROD<br>Department of genetics<br>HFME A1 - 59 bd Pinel - 69677 BRON cedex<br>Phone: 04 27 85 65 25 Fax: 04 27 85 67 98<br>Email: sophie.dupuis-girod@chu-lyon.fr
**PROTOCOL VERSION**	V4 dated 24/03/2022
**JUSTIFICATION/ CONTEXT**	HHT is a rare but ubiquitous hereditary vascular disease, with estimated prevalence of 1/6000. It is related to disequilibrium in the angiogenic balance, resulting from abnormal homeostasis between the factors involved in the activation phase and those involved in the maturation phase of angiogenesis. *ENG* (Endoglin) and *ACVRL1* encoding ALK1 (Activine receptor like kinase 1) genes are responsible for at least 92% of cases of HHT. These genes both intervene in the signaling pathway of the Transforming growth factor beta (TGFbeta) family in endothelial cells.<br>The recognized manifestations of HHT are all due to abnormalities in vascular structure. Lesions may be small (telangiectases) or large arteriovenous malformations (AVMs). Telangiectases and AVMs vary widely between individuals and even within the same family. Epistaxis are spontaneous, very variable, may occur as often as several times every day, and are recurrent in 90% of patients and associated with chronic and severe anemia in 2-10%. They also significantly reduce quality of life.<br>Blood transfusions are sometimes required in 10-30% of patients, but are regularly required (every 2 or 3 weeks) in 2-5% of patients. The absence of proved efficient treatment has inspired a new search for treatments which would greatly diminish daily iron loss. According to physiopathological mechanisms implicated in HHT, anti-angiogenic treatments appeared to be very promising drugs.<br>Previous studies showed that anti-VEGF treatment (bevacizumab) administered intravenously was efficient on epistaxis and dramatically reduced nosebleeds, but its use is currently limited to severe forms of the disease due to the administration route, price and no market authorization.<br>Tyrosine kinase inhibitors are anti-angiogenic molecules which are available orally and could therefore overcome the difficulties encountered with bevacizumab. The tyrosine kinase inhibitor nintedanib targets growth factor receptors involved in angiogenesis. Most importantly it inhibits the platelet-derived growth factor receptor (PDGFR), fibroblast growth factor receptor (FGFR) and vascular endothelial growth factor receptor (VEGFR). Therefore, we hypothesize that nintedanib, acts by indirect inhibition of the VEGF receptor and should allow a reduction of epistaxis in HHT patient.<br>Nintedanib has been used in one HHT patient following the diagnosis of Insterstitial Pulmonary Fibrosis (published case report in 2017, Kovacs et al). His Epistaxis Score Severity dramatically decreased from a moderate score of 5.5 to a mild value of 0.5.<br>Pazopanib, another tyrosine kinase inhibitor, has been tested at a dose of 50 mg/day in HHT by Faughnan et al. and showed promising results in treatment of HHT related bleeding (NCT02204371).<br>These results encouraged us to conduct a clinical study to evaluate efficacy of nintedanib for the treatment of epistaxis in HHT patients. Moreover, nintedanib is a suitable candidate because of its administration route (oral) and its safety profile (side effects with nintedanib can be managed in most patients).
**OBJECTIVES**	- **Primary objective:**<br>The primary objective is to evaluate efficacy, at the end of the treatment period, on epistaxis duration of nintedanib treatment per os (300 mg/day for 12 weeks) versus placebo in HHT patients with moderate to severe epistaxis.<br>**- Secondary objective:**<br>1. To evaluate nintedanib safety in HHT patients.<br>2. To evaluate efficacy of nintedanib treatment on epistaxis (duration, frequency and severity).

	3. To evaluate efficacy of nintedanib treatment on other clinical criteria: Quality of life: SF36, number of red blood cell transfusions and number of iron infusions.<br>4. To evaluate efficacy of nintedanib treatment on biological criteria: hemoglobin and ferritin levels.
**METHODOLOGY/DIAGRAM OF THE STUDY**	Multicenter, randomized versus placebo (ratio 1:1) study carried out in a double blind setting.<br>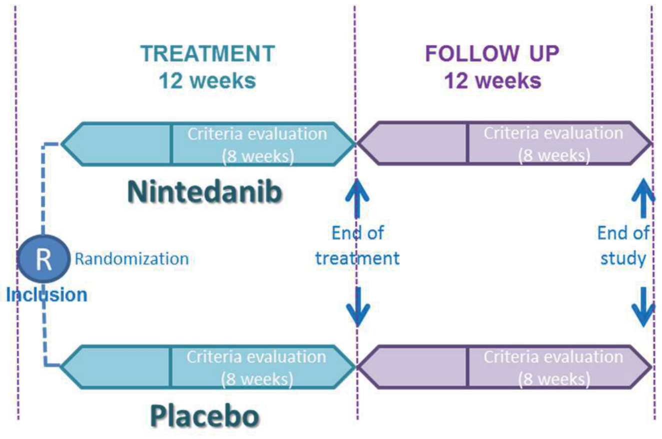
**ENDPOINTS**	- **Primary endpoint**<br>Proportion of participants reporting a response at the end of treatment. A response is defined as a reduction of at least 50% on epistaxis monthly mean duration during the last 8 weeks of treatment as compared to the 8 weeks before treatment. This criterion will be assessed on the basis of monitoring of epistaxis grids filled in by the patients (collected at each visit of filled in online).<br>- **Secondary endpoints**<br>1. All adverse events and severe adverse events observed during the study will be collected.<br>2. Epistaxis will be assessed through<br>– Proportion of patients reporting a response at the end of follow-up. A response is defined by a reduction of at least 50% on epistaxis monthly mean duration during the last 8 weeks of follow-up as compared to the 8 weeks before treatment. Assessment by epistaxis grids filled in by patients (collected at each visit or filled in online).<br>– Nosebleeds monthly mean duration (continuous variable) will be computed during the 8 weeks before treatment, during the last 8 weeks of the treatment period and during the last 8 weeks of the follow-up period. Differences from baseline will be assessed. Assessment by epistaxis grids filled in by patients (collected at each visit or filled in online).<br>– Nosebleeds monthly mean duration (continuous variable) will be computed all over the study period using 4 weeks periods. Assessment by epistaxis grids completed by patients (collected at each visit or filled in online).<br>– Nosebleeds frequency (considered as continuous variable) will be computed during the 8 weeks before treatment, during the last 8 weeks of the treatment period and during the last 8 weeks of the follow-up. Differences from baseline will be assessed. Assessment by epistaxis grids filled in by patients (collected at each visit or completed online).<br>– Epistaxis score = ESS (continuous variable) will be calculated from questionnaire filled in by patients at inclusion visit, at the end of the treatment period and at the end of the follow-up.<br>3. Other clinical criteria :<br>– Quality of life = SF36 score based on questionnaire filled in by patients at inclusion visit (baseline), at the end of the treatment and end of the follow up will be calculated.<br>– Number of red blood cell transfusions (discrete variable) is collected for 8 weeks before treatment, during the last 8 weeks of the treatment period and during the last 8 weeks of the follow-up period.<br>– Number of iron infusions (discrete variable) is collected for 8 weeks before treatment, during the last 8 weeks of the treatment period and during the last 8 weeks of the follow-up period.<br>4. Biological criteria:<br>– Hemoglobin level (continuous variable) will be measured at inclusion, at the end of the treatment visit and at the end of follow-up visit.

	– Ferritin level (continuous variable) will be measured at inclusion visit, at the end of the treatment visit and at the end of follow-up visit.
**TARGET POPULATION**	This study will involve adult patients suffering from moderate to severe epistaxis related to HHT, which responsible for severe alterations in social functioning and quality of life. This parameter is evaluated with an Epistaxis Severity Score. It has been established that patients with epistaxis from moderate to severe, that is to say a score ESS> 4, have a poor quality of life.
**INCLUSION CRITERIA**	– Age > 18 years old<br>– Patients who have given their free informed and signed consent<br>– Patients affiliated to a social security scheme or similar<br>– Patients monitored for clinically confirmed HHT and/or with molecular biology confirmation<br>– Patient with an Epistaxis Severity Score (ESS) > 4
**NON-INCLUSION CRITERIA**	– Pregnant woman or woman of child bearing potential not using two effective methods of birth control (one barrier and one highly effective non-barrier) for at least 1 month prior to trial and/or committing to using it until 3 months after the end of treatment.<br>– Woman who are breast feeding<br>– Patient who are protected adults under the terms of the law (French Public Health Code)<br>– Participation in another interventional clinical trial which may interfere with the proposed trial (judgment of the investigator)<br>– Clinical evidence of active infection<br>– AST, ALT > 1,5 fold upper limit of normal (ULN) and/or Bilirubin > 1,5 fold upper limit of normal (ULN)<br>– Severe renal impairment (Creat Clearance <30 mL/min) estimated by the Cockcroft-Gault equation<br>– Presence of non-treated pulmonary arteriovenous malformations accessible to a treatment on CT scan within 5 years.<br>– Patients with haemoptysis or haematuria within 12 weeks prior to inclusion<br>– Patients with active gastro-intestinal (GI) bleeding or GI ulcers within 12 months prior to inclusion<br>– Presence of cerebral arteriovenous malformation on MRI done within 5 years prior inclusion.<br>– Patients who require full-dose therapeutic anticoagulation (e.g. vitamin K antagonist or heparin, dabigatran) or high dose antiplatelet therapy, patients under anticoagulation with rivaroxaban, apixaban and epixaban<br>– Patients with P-glycoprotein (P-gp) substrates/inducers/inhibitors (e.g.: ketoconazole, erythromycin, cyclosporine, rifampicin, carbamazepine, phenytoin, and St. John's Wort).<br>– Patients with known coronary artery disease or with a recent history of myocardial infarction (within 1 year)<br>– Known inherited predisposition to thrombosis or thrombotic events (including stroke and transient ischemic attack, excluded superficial venous thrombosis) within 12 months prior to inclusion<br>– Patients with QTc prolongation (on ECG less than 3 months)<br>– Hypersensitivity to nintedanib, peanut or soya, or to any of the excipients.<br>– Patient who incompletely filled in epistaxis grids within 8 weeks prior to inclusion<br>– Patient who have received intravenous bevacizumab within 6 months prior to inclusion<br>– Patient who had surgery (including ENT surgery) within 12 weeks prior to inclusion<br>– Unhealed wound<br>– Planned major surgery within the next 3 months, including liver transplantation, major abdominal or intestinal surgery.
**CRITERIA FOR WITHDRAWAL FROM THE STUDY**	- **Criteria for premature termination**<br>If a patient is included and randomized and did not took any treatment he(she) will be withdrawn from the study and replaced.<br>If the treatment is prematurely or temporary stopped by the patient, due to complications, adverse events, adverse reaction, introduction of a forbidden associated treatment or logistical reasons, the patient will not be replaced and will not be excluded from the study but will be followed-up for the 24 weeks after the beginning of the treatment as planned for the study.<br>In case of intercurrent events, the treatment can be prematurely or temporary stopped or delayed. It is up to the investigator to make the decision in the interest of the patient, on the basis of the balance between the risk of a new event and the risk of aggravation of the disease.

<table>
<tr>
<td></td>
<td>
- Rules for a temporary or permanent discontinuation
  - Discontinuation of the participation of a subject in the study:
The subjects can withdraw their consent and request to leave the study at any time and for any reason. The investigator may temporarily or definitively interrupt a subject's participation in the study for any reason which would best serve the interests of the subject, particularly in the event of a serious adverse event. In the event of a withdrawal of consent, the data collected up to the date of withdrawal will be analyzed
  - Termination of part or all of the study:
The study can be prematurely suspended in the event of unexpected serious adverse events requiring a review of the characteristics of the strategy. Likewise, unexpected events or new information concerning the method of investigation, in view of which the objectives of the study are not likely to be met, may lead the sponsor to prematurely suspend the study. The Hospices Civils de Lyon reserves the right to suspend the study at any moment if it is determined that the inclusion objectives are not reached.
</td>
</tr>
<tr>
<td>PROCEDURES</td>
<td>
The treatment tested is nintedanib, manufactured by Boehringer Ingelheim International GmbH and commercialized as OFEV®, 100 mg and 150 mg soft capsules.
Treatment of comparison is placebo: identical soft capsules containing a suspension of titanium dioxide as drug substance substitute.
Posology: 300 mg nintedanib or placebo per day, 150 mg twice daily administered approximately 12 hours apparts, for 12 weeks. There will be 12 weeks follow-up after the end of the treatment.

In case of poor tolerance posology could be reduced to 200 mg per day (100 mg twice daily).

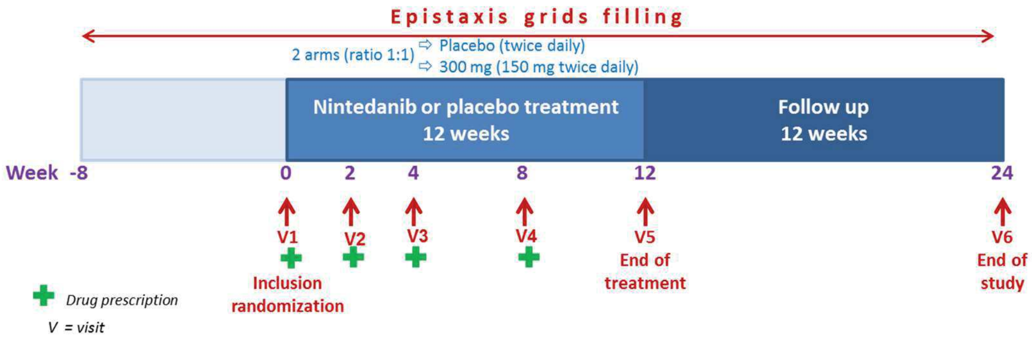

</td>
</tr>
<tr>
<td>BENEFIT-RISK RATIO</td>
<td>
Side effects have been studied in oncology and pulmonary fibrosis. We thus evaluated the risks of such a treatment in HHT.

The most frequently reported adverse reactions associated with the use of nintedanib included diarrhea, nausea and vomiting, abdominal pain, decreased appetite, weight decreased and hepatic enzyme increased.

Protection against study risks: Patients will be informed about the risks. All adverse reaction will be carefully monitored regularly throughout the study. In case of apparition, the treatment could be interrupted according to investigator's brochure recommendations. Gastrointestinal disorders will be treated (diarrhea will be treated with adequate hydratation and anti-diarrheal medicinal product). A leaflet will be provided with the treatment, and it will include the behavior to have in case of adverse event. A participation card will be provided as well with emergency phone number. Patients with a high hemorrhage risk (cerebral AVM, not treated pulmonary AVM, hemoptysis, hematuria, active gastrointestinal bleeding or GI ulcers) will not be included. To prevent arterial thromboembolic events, patients with known coronary artery disease or with a recent history (<1 year) of myocardial infarction or stroke will not be included.

Potential Benefits: The expected benefits are: to decrease epistaxis duration, to improve clinical condition of the patient with moderate and severe nosebleeds, to improve quality of life, to decrease the number of hospitalizations for red blood cells transfusions and/or iron infusions, and to increase in hemoglobin and serum ferritin levels.

The benefit risk balance appears to be very favorable.
</td>
</tr>
<tr>
<td>NUMBER OF SUBJECTS</td>
<td>60 patients (30 in each group)</td>
</tr>
</table>

**DURATION OF THE STUDY**	Duration of the inclusion period: 30 months<br>Length of the participation of each subject: 24 weeks (about 6 months)<br>Total duration of the study: 36 months
**LOCATION OF THE STUDY**	Patients will be included in the study at the reference center for HHT in Lyon (principal investigator and coordinator) and at 9 skill centers distributed over French territory.
**EXPECTED IMPACTS**	The expected impact is enormous. Indeed, the most apparent expression of the disorder is the occurrence of spontaneous, repeated epistaxis. These epistaxis can be severe and disabling; they are often the cause of chronic anemia, and can require continuous management including repeated hospitalizations for iron supplementation and blood transfusions. Furthermore, epistaxis are responsible for major social consequences in HHT patients. Currently no treatment decrease significantly nosebleeds. Surgical treatments are aggressive for the nasal mucosa and risk perforating the nasal septum. No local drug treatment could show efficacy. Bevacizumab use is currently limited to severe forms of the disease due to the administration route, price and absence of market authorization.<br>An oral treatment of nintedanib, if well tolerated, could be an interesting therapeutic option.

LIST OF ABBREVIATIONS

**ACVRL1**	Activin receptor- like type 1
**AE**	Adverse event
**ALK1**	Activin receptor-like-kinase-type 1
**ALT**	Alanine Aminotransferase
**ANSM**	French National Agency for Medicines and Health Products Safety (Agence Nationale de Sécurité des Médicaments et des produits de santé)
**AR**	Adverse Reaction
**AST**	Aspartate Aminotransferase
**AVF**	ArterioVenous Fistula
**AVM**	ArterioVenous Malformation
**B-HCG**	B-Human Chorionoc Gonadotropin
**BMP**	Body Morphogenetic Protein
**BRV**	Biomedical Research Volunteers
**CHU**	Centre Hospitalier Universitaire (University teaching Hospital)
**CNIL**	French national commission for data protection and freedom of information (Commission Nationale Informatique et Liberté)
**CRA**	Clinical Research Associate
**CRF - eCRF**	Case Report Form – electronic Case Report Form
**CST**	Clinical Study Technician
**CTCAE**	Common Terminology Criteria for Adverse Events
**CT scan**	Computerized Tomography scanner
**EC**	Ethics committee (Comité de Protection des Personnes)
**ECG**	Electrocardiogram
**ENG**	Endogline
**ENT**	Ear, Nose and Throat Specialist
**ESS**	Epistaxis Severity Score
**DSMB**	Data and Safety Monitoring Board
**FGFR**	Fibroblast Growth Factor Receptor
**GCP**	Good Clinical Practice
**GGT**	Gamma Glutamyl-Transpeptidase
**GMP**	Good Manufacturing Practice
**GI**	Gastrointestinal
**Hb**	Hemoglobin
**HCL**	Hospices Civils de Lyon
**HDPE**	High Density Poly Ethylene
**HHT**	Hereditary Hemorrhagic Telangiectasia
**HP**	Hospital pharmacy
**ICH**	International Conference on Harmonization
**IMER**	Information Médicale Evaluation Recherche (Medical Information Research Assessment)
**IPF**	Idiopathic Pulmonary Fibrosis
**IWRS**	Interactive Web Response System
**KO**	Knock Out

**MA**	Marketing Authorization
**NFS**	Numératin Formule Sanguine (CBC = Complete Blood Count)
**PAL**	Alkaline Phosphatase
**PAVM**	Pulmonary ArterioVenous Malformation
**PDGFR**	Platelet-derived growth factor receptor
**PNDS**	Protocole National de Diagnostic et de Soin (French National Protocol for Diagnoses and Healthcare)
**RM**	Reference Methodology
**SAE**	Serious Adverse Event
**SAS**	Statistical Analysis System
**SF36**	Short Form 36
**SMAD4**	Mothers Against Dpp homologue 4
**SPC**	Summary of Product Characteristics
**SUSAR**	Suspected Unexpected Serious Adverse Reaction
**TGF-β**	Transforming Growth Factor-β
**ULN**	Upper Limit of Normal
**VEGF**	Vascular Endothelial Growth Factor
**VEGFR**	Vascular Endothelial Growth Factor Receptor

TABLE OF CONTENTS

LIST OF TABLE 11
1 GENERAL INFORMATION 12
1.1. Title 12
1.2. Project identifiers and history of previous updates 12
1.3. Sponsor 12
1.4. Investigators 14
1.4.1. Principal Investigator 14
1.4.2. Associated investigators 14
1.5. Methodologist-Biostatistician 15
1.6. Pharmacist 15
1.7. Committees 15
1.7.1. Scientific committee 15
1.7.2. Independent monitoring committee 15
1.7.3. Adverse events assessment committee 15
2 SCIENTIFIC JUSTIFICATION 15
2.1 Current state of knowledge - rational 15
2.1.1 Hereditary Hemorrhagic Telangiectasia (HHT) 15
2.1.2 Bleedings in HHT (Nosebleeds) 17
2.1.3 Angiogenesis and its regulation 18
2.1.4 Angiogenesis in HHT 19
2.1.5 Anti-angiogenic treatments in HHT 21
2.2 Hypothesis of the study 23
2.3 Justification of the methodological choices 24
2.4 Target population 24
2.5 Benefit-risk ratio 25
2.6 Expected impacts 28
3 STUDY OBJECTIVES 29
3.1 Primary objective 29
3.2 Secondary objective 29
4 STUDY DESIGN 29
4.1 Type of study 29
4.2 Randomization method 29
4.3 Endpoints 29
4.3.1 Primary endpoint 29
4.3.2 Secondary endpoints 30
5 ELIGIBILITY CRITERIA 31
5.1 Inclusion criteria 31
5.2 Non-inclusion criteria 31
5.3 Criteria for premature termination 32
5.4 Recruitment methods and feasibility 33
6 EXPERIMENTAL TREATMENTS 33
6.1 Study treatment 33
6.2 Treatment of comparison 34
6.3 Product circuits 34
6.4 Blinding 35
6.5 Authorized and forbidden associated treatments 35
7 GENERAL ORGANIZATION 36
7.1 Study calendar 36
7.2 General diagram and summary table 36
7.3 Conduct of the study 37
7.3.1 Screening –Pre-inclusion 37

7.3.2 Inclusion visit/Randomization (day 0) 37
7.3.3 Follow-up visits 38
7.3.4 End of study visit at 24 weeks, V6 (Day 168 from D158 to D178) 40
7.4 Rules for treatment discontinuation and dose adjustment 40
7.5 Rules for a temporary or permanent discontinuation 41
8 SAFETY ASSESSMENT 41
8.1 Definitions 41
8.1.1 Adverse event 41
8.1.2 Adverse reaction 41
8.1.3 Unexpected adverse reaction 41
8.1.4 Serious Adverse Events (SAE) 41
8.1.5 New issue 42
8.2 Investigator's responsibilities 42
8.2.1 Procedures for detection and reporting of the adverse events 42
8.2.2 Serious adverse event reporting 42
8.2.3 Serious adverse events that do not require prompt notification to the sponsor (no SAE form to send but SAE to collect only in the CRF) 43
8.2.4 Adverse Events with specific interest (AESI) 43
8.2.5 In utero exposition 44
8.2.6 Causality assessment 44
8.2.7 Reporting time frames of SAE without delay to the sponsor by the investigator and procedures for monitoring serious adverse events 44
8.3 Specific management of complications 44
8.3.1 Gastrointestinal disorders 44
8.3.2 Hepatic function 45
8.3.3 Arterial thromboembolic events 46
8.3.4 Surgery 46
8.3.5 Hemorrhages 46
8.4 Responsibility of the sponsor 47
8.4.1 Declaration to the competent authorities 47
8.4.2 Safety reference information for the assessment of the expectedness / unexpectedness ... 47
8.5 Data Safety Monitoring Board (DSMB) or Data Monitoring Committee (DMC) 47
9 STATISTICAL CONSIDERATIONS 48
9.1 Number of subjects required 48
9.2 Description of the statistical methods 48
9.2.1 Study populations 48
9.2.2 Protocol deviations 48
9.2.3 Baseline characteristics 48
9.2.4 General considerations 48
9.2.5 Main judgment criterion analysis (intention-to-treat population) 48
9.2.6 Secondary judgment criterion analysis 48
9.3 Method of accounting for missing data 49
9.4 Management of modifications brought to the analysis plan 50
9.5 Person responsible for the analyses and the software used 50
10 MONITORING THE STUDY 50
10.1 Scientific committee 50
10.2 Independent monitoring committee 50
10.3 Adverse events assessment committee 50
11 RIGHT OF ACCESS TO THE DATA AND SOURCE DOCUMENTS 51
11.1 Access to the data 51
11.2 Source documents 51
11.3 Data confidentiality 51
12 QUALITY CONTROL AND ASSURANCE 51

13 Ethical considerations 52
13.1 Competent authorities 52
13.2 Substantial modifications 52
13.3 Patient information and written consent form 52
13.4 Declaration of conformity 52
13.5 Exclusion period 52
13.6 Compensation for the subjects and registration in the national register of people participating in a cat. 1 interventional study on human individuals 53
14 MANAGEMENT AND STORAGE OF DATA 53
14.1 Electronic Case report form 53
14.2 Electronic Data management 53
14.3 CNIL 53
14.4 Archiving 53
15 FUNDING AND INSURANCE 54
15.1 Study budget 54
15.2 Insurance 54
16 RULES RELATING TO THE PUBLICATION 54
17 REFERENCES 55

LIST OF FIGURES

Figure 1: Telangiectasia in HHT 16
Figure 2: Angiogenesis results from an activation phase and a maturation phase (from David L. [28]) 18
Figure 3: BMP9/ALK1/endoglin pathway (taken from S. Bailly[30]) 19
Figure 4: Evolution of a Cutaneous Telangiectasis in HHT (from Guttmacher et al[34]) 20
Figure 5: Angiogenic balance in HHT (taken from S. Bailly[30]) 20
Figure 6: Significant decrease in nosebleeds after six IV infusions of bevacizumab 21
Figure 7: Angiogenesis and potential targets for treatments 22
Figure 8: Case report, Hemoglobin (g/dL) trend over time (months) 23
Figure 9: Study design 24
Figure 10: ESS score and Target population 25
Figure 11: incidence of diarrhea and other adverse events of interest from Noth et al[54] 27
Figure 12: Primary endpoint evaluation 30
Figure 13: Secondary endpoints evaluations 31
Figure 14: General diagram of the study 36
Figure 15: Adverse Event Flow Chart 44
Figure 16: Recommendations for the management of diarrhea adverse events in the study 45
Figure 17: Recommendations for the management of hepatic enzyme elevations in the study 46

LIST OF TABLE

Table 1: Adverse events in a pooled analysis of data from patients treated with nintedanib 150 mg twice daily or placebo in the TOMORROW and INPULSIS® trials (mean exposure 10.2 months for nintedanib and 10.8 months for placebo)[55]. 26
Table 2: synopsis of the study per patient 37

1 GENERAL INFORMATION

1.1. Title

EPICURE: Efficacy of Nintedanib per os as a treatment for epistaxis in HHT disease. A national, randomized, multicenter phase II study.

1.2. Project identifiers and history of previous updates

Sponsor code: 69HCL19_0003
Clinicaltrials.gov registration number: *NCT03954782*
Favorable opinion from EC Ouest I on: 05/11/2019
ANSM authorization on: 31/10/2019

History of previous versions		
**Version**	**Date**	**Reason for the update**
1	29/08/2019	Initial version
2	16/10/2019	Following ANSM questions
3	17/07/2020	SAE declaration<br>Epistaxis grids<br>Statistical points<br>AE
4	24/03/2022	Increase of the inclusion period

1.3. Sponsor

- *Identity:*

Hospices Civils de Lyon
BP 2251
3 Quai des Célestins
69229 LYON Cedex 02

- *Signatory of the protocol on behalf of the sponsor:*

Alexandre PACHOT, Director of Clinical Research and Innovation
Hospices Civils de Lyon, Direction de la Recherche en Santé, Siège Administratif, BP 2251, 3 Quai des Célestins, 69229 LYON Cedex 02
Phone: 0033 (0)4 72 40 68 52 Fax: 0033 (0)4 72 40 68 69

- *Head of study for the sponsor:*

Valérie PLATTNER, primary physician
Hospices Civils de Lyon, Direction de la Recherche en Santé, Siège Administratif, BP 2251, 3 Quai des Célestins, 69229 LYON Cedex 02
Phone: 0033 (0)4 72 40 68 40 Fax: 0033 (0)4 72 11 51 90

Thierry HEREMBERT
Hospices Civils de Lyon, Direction de la Recherche en Santé, Siège Administratif, BP 2251, 3 Quai des Célestins, 69229 LYON Cedex 02
Tél : 04 72 40 70 87, Fax : 04 72 11 51 90

- *Head of clinical trials 'vigilance for the sponsor:*

Marina NGUON, primary pharmacist
Hospices Civils de Lyon, Direction de la Recherche en Santé, Siège Administratif, BP 2251, 3 Quai des Célestins, 69229 LYON Cedex 02

Phone: 0033 (0)4 72 40 68 26 Fax: 0033 (0)4 72 11 51 90

1.4. Investigators

1.4.1. Principal Investigator

Docteur Sophie DUPUIS-GIROD
Department of genetics
HFME A1 - 59 bd Pinel - 69677 BRON cedex
Phone: 04 27 85 65 25 - Fax: 04 27 85 67 98
Email : sophie.dupuis-girod@chu-lyon.fr

1.4.2. Associated investigators

Investigateurs	Adresse du Centre	Téléphone, E Mail
Dr Frédéric FAURE<br>Dr Ruben HERMANN	Service ORL<br>Hôpital Edouard Herriot<br>5, place d'Arsonval<br>69437 LYON cedex 03	04 72 11 05 40<br>frederic.faure@chu-lyon.fr<br>ruben.hermann@chu-lyon.fr
Dr S. RIVIERE	CHU de Montpellier<br>Hôpital St Eloi<br>Service de médecine Interne A<br>Av. Augustin Fliche<br>34295 MONTPELLIER Cedex	04 67 33 73 37<br>s-riviere@chu-montpellier.fr
Pr B. GILBERT-DUSSARDIER	CHRU - Hôpital J.Bernard<br>Service de génétique<br>BP.577<br>86021 POITIERS Cedex	05 49 44 39 32<br>brigitte.gilbert-dussardier@chu-poitiers.fr
Dr H. MAILLARD	Hôpital Jeanne de Flandre<br>Service de médecine interne<br>2, av. Oscar Lambret<br>59037 LILLE Cedex	03 20 44 42 95<br>helene.maillard@chru-lille.fr
Dr M. KERJOUAN	CHU de Rennes<br>Hôpital Pontchaillou<br>Service de Pneumologie<br>35033 RENNES cedex	02 99 28 37 38<br>mallorie.kerjouan@chu-rennes.fr
Pr T. CHINET	Hôpital Ambroise Paré<br>Service de radiologie et de pneumologie interventionnelle<br>9 av Charles de Gaulle<br>92104 BOULOGNE BILLANCOURT	01.49.09.55.49<br>thierry.chinet@aphp.fr
Dr J SEGUIER	CHU de Marseille<br>Service de médecine interne<br>Hôpital la conception<br>13385 MARSEILLE	04.91.38.35.01<br>julie.seguier@ap-hm.de
Pr A PARROT	Hôpital Tenon<br>Service d'imagerie et de radiologie interventionnelle<br>4, rue de la Chine<br>75790 PARIS Cedex 20	01 56 01 66 70<br>antoine-parrot@tnn.aphp.fr
Dr C. LAVIGNE	CHU d'Angers<br>Service de médecine interne<br>4, rue Larrey<br>49933 ANGERS cedex 09	02 41 35 77 00<br>chlavigne@chu-angers.fr
Dr V GROBOST	CHU Clermont Ferrand<br>Service de médecine interne<br>Hôpital Estaing<br>63100 CLERMONT-FERRAND	04 73 75 00 85<br>vincent.grobost@gmail.com

1.5. Methodologist-Biostatistician

Dr Evelyne DECULLIER
Unité de Recherche Clinique, Pôle Santé Publique
162, Avenue Lacassagne - 69424 Lyon Cedex 03
04 72 11 57 06 / evelyne.decullier@chu-lyon.fr

1.6. Pharmacist

Dr Caroline GERVAISE
Service pharmaceutique – Secteur Essais cliniques
Groupement Hospitalier Est
59, Bd Pinel
69677 BRON
04 72 35 75 50
caroline.gervaise@chu-lyon.fr

1.7. Committees

1.7.1. Scientific committee

The Scientific Committee for the study, presided over by the study's main investigator, will be composed of investigators (one ENT specialist and one geneticist), the methodologist and the sponsor of the study. The committee will be responsible for validating the definitive version of the protocol, supervising the implementation and running of the study, and for writing the reports and publications resulting from the study.

1.7.2. Independent monitoring committee

Monitoring the safety of administration of the product, motivated by the iatrogenic risks, justifies the setting up of a specific independent monitoring and safety committee (Data Safety Monitoring Board = DSMB).

This committee will meet at least once a year and in case of occurrence of serious adverse events evoking a toxicity of the treatment under study. It gives recommendations regarding protection of the safety of the study participants. It will be composed of people not involved in the study:

- a specialist of the disease,
- a pharmacist,
- a statistician specializing in the methodology of clinical trials.

1.7.3. Adverse events assessment committee

This committee will be composed of investigators involved in the study, HHT specialists, in each of the centers in charge of patients in the study, and the methodologist. A representative of safety department from the sponsor will be invited.

They will meet regularly, about twice a year according to the study advancement. All adverse events collected in the eCRF will be evaluated and graduated.

2 SCIENTIFIC JUSTIFICATION

2.1 Current state of knowledge - rational

2.1.1 Hereditary Hemorrhagic Telangiectasia (HHT)

Hereditary Hemorrhagic Telangiectasia (HHT) is a rare but ubiquitous hereditary vascular disease with a prevalence 1/6,000.

The French HHT National reference center received its label in 2004. With 16 skill centers, it has a cohort of more than 2,000 patients managed both clinically and genetically.

2.1.1.1 Clinical picture

The severity, age of onset and locations of the vascular lesions are extremely variable from one individual to another.

The diagnosis of HHT is based on several criteria, known as the Curaçao criteria [1]:

1. The hereditary element: Transmission is dominant autosomal (50% to each child).

2. Telangiectasias. These are lesions that are characteristic of the disease and are cutaneous (lips, fingers, face, hands and feet) and mucosal (inside of the lips, tongue, palate, nasal and digestive mucosae). (Figure 1)

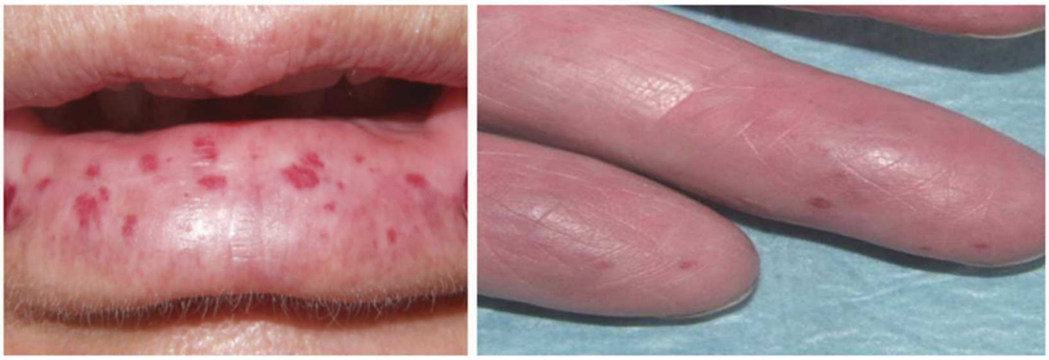

Figure 1: Telangiectasia in HHT

3. Epistaxis (nosebleeds): These are the main form of expression of these telangiectasias both in terms of their frequency and the handicap that they provoke. Nosebleeds affect more than 95% of patients. They are spontaneous, repeated, irregular, diurnal and nocturnal; they lead to anemia and are both disabling and socially embarrassing. Chronic disabling anemia is a predominant consequence in these patients.

4. Visceral arteriovenous malformations (AVMs): These are vascular lesions whose impact is always aggravated in cases of anemia. Visceral involvement can replace one of the three main external signs in a positive diagnosis. The localization of these AVF can be:

- pulmonary (6 to 60% of patients[2-5]). The treatment has now been codified and is based on radiological vaso-occlusion. Preventing hemorrhagic rupture, cerebral complications (abscess, stroke and transitory ischemic attacks) and pulmonary signs (dyspnea, cyanosis and polyglobulitis, consequences of hypoxia) are the primary goals of treatment.

- neurological, cerebral or medullary[6]. Prevention of these lesions is problematic during the asymptomatic phase. Scanning for these lesions is controversial [7-9] and their presence is evaluated at between 5 and 23% of patients with neurologically asymptomatic HHT[1,10,11].

- hepatic [12,13]. Their definition is based on several radiological criteria. Their progress leads certain patients to a liver transplant[14-17].

The clinical diagnosis is
- certain if at least 3 of the criteria are present,
- suspected or possible if 2 of the criteria are present,
- unlikely if only 1 of the criteria is present.

Typical visceral complication can form one of the three criteria required for the diagnosis, thus replacing the nosebleeds, telangiectasias or the hereditary nature [1]. Some patients can have 4 or 5 signs of the disease, with several forms of visceral involvement.

Molecular diagnosis is available for the *ALK1, ENG*, and *SMAD4* genes and mutations are found in around 95% of patients with a definite clinical diagnosis[18]. Molecular diagnosis is currently used in order to screen asymptomatic patients and to avoid previously described complications (cerebral abscess, stroke, hemorrhage…).

2.1.1.2 Genetics and physiopathology

There are three genes known to be responsible for HHT:

- Endogline (*ENG*), responsible for the HHT1 phenotype of the disease.
- Activin-receptor-Like-Kinase-type 1 (*ALK-1*), responsible for the HHT2 phenotype of the disease.
- *SMAD 4*, responsible for a rarer phenotype that associates HHT and chronic juvenile polyposis.

ENG and *ALK1* are responsible for at least 92% of HHT cases. These genes both intervene in the signaling pathway of the TGFβ family in endothelial cells.

2.1.2 Bleedings in HHT (Nosebleeds)

Nosebleeds are the main expression of HHT and are often the most disabling complication of the disease in terms of quality of life and morbidity.

They are the motivation for repeated periods of sick leave and even on occasion a classification of disability[19]. The duration of the nosebleeds may be greater than 24 hours per month in patients, and may require repeated blood transfusions and hospitalizations. As a result, the anemia may be severe, and the repeated ENT treatments have a risk of complication (perforation of the nasal septum, infections after packing).
Their objective assessment is achieved by means of a grid where the number of nosebleeds per month and the duration of the bleeding are recorded (Appendix 1: Nosebleed monitoring grid).
Furthermore, a validated scoring index, the Epistaxis Severity Score (ESS), was developed as a standardized measurement of patient-reported epistaxis severity[20]. This score summarizes the severity of epistaxis symptoms along multiple domains, including frequency and intensity of nosebleeds, duration of epistaxis episodes, and medical consequences of recurrent epistaxis such as anemia and dependence on blood transfusions. An increase in the ESS was significantly correlated to a decline in health related quality of life, as measured by the Medical Outcomes Study 36-Item Short Form (SF-36).[21]

- Nosebleed treatment

Efficient and authorized product does not exist for HHT treatment today.
Treatment can include:

1. Local treatment: Epistaxis prevention using regular (several times a day) humidification of the nasal mucosa by the patient by means of an association of pomades and physiological serum makes it possible to partially improve the nosebleeds. Epistaxis treatment by compressing techniques, use of wicks made out of spongy and resorbable material or expansive, resorbable gels and selective
2. Surgery: bilateral embolization or surgical ligature of the vessels [22]. Depending on the severity and disability caused, different ENT techniques may be used: laser [23], sclerosing drugs, vascular embolism of red spots on the nose, septal dermoplast. There is no current surgical treatment that makes it possible to cure the nosebleeds definitively. The repetition of these treatments is often the source of significant iatrogenic conditions, including the perforation of the nasal septum, resulting in a worsening of the nosebleeds.
3. Medical: Tranexamic acid, an anti-fibrinolytic drug, has been used in the treatment of HHT-related epistaxis [24-26] and is prescribed in European countries and Canada but its efficacy is moderate [27].
   Treatment by estrogens and progestogens has been tested with some success, but the treatment is somewhat problematic in men and the indication for these drugs in menopausal women over 50 is debatable with recent observations showing their adverse carcinogenic and cardiovascular effects.

No studies with a "high level of proof" have shown the efficacy of any medical or surgical treatments.
In addition to the treatments already mentioned,

- Anemia

Iron supplementation is recommended for all patients with repeated bleeding resulting in long term iron deficiency anemia. Patients with poor tolerance of oral iron supplementation may benefit from intravenous iron injections every 3 weeks. Blood transfusions are carried out in accordance with the recommendations of the ANSM (French National Agency for Medicines and Health Products).
Management of anemia is still a key element and iron replacement therapy is advised for any patient with repeated epistaxis leading to long-term iron deficiency anemia. Patients who are intolerant of oral iron can be treated with an intravenous preparation.
All these treatments are only symptomatic.

2.1.3 Angiogenesis and its regulation

Angiogenesis is the formation of new blood vessels from an existing vascular network. Angiogenesis is the *de novo* formation of vessels from the pre-existent vascular tree, in response to a stimulus. This biological process is controlled by pro-angiogenic factors that promote vascular growth and angiostatic factors that induce vascular regression. Physiological angiogenesis occurs during development and in healthy individuals, in wound injury and repair, menstruation, pregnancy. There are two phases in angiogenesis: an activation phase in which the extracellular matrix is degraded and the endothelial cells migrate and proliferate (*Figure 2*). VEGF is one of the key factors in this activation phase. This phase is followed by a maturation phase in which the endothelial cells stop migrating and proliferating, the cellular matrix is reconstituted and there is recruitment of mesenchymatous cells which differentiate into pericytes or smooth muscle cells, depending on the type of vessel. Angiogenesis is generally quiescent in adults.

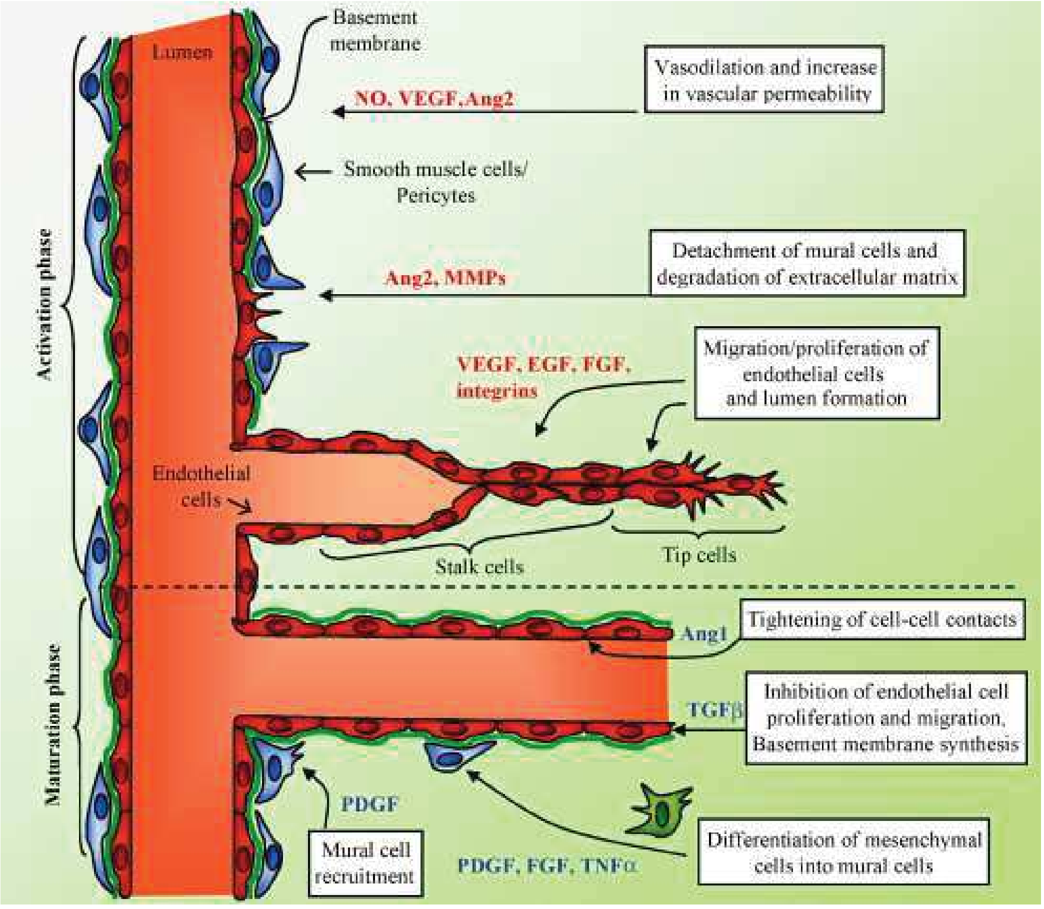

Figure 2: Angiogenesis results from an activation phase and a maturation phase (from David L. [28])

(NO: nitric oxide, VEGF: vascular endothelial growth factor, Ang2: angiopoietin 2, Ang1: angiopoietin 1, MMPs: matrix metalloproteinases, EGF: epidermal growth factor, FGF: fibroblast growth factor, TGFb: transforming growth factor beta, PDGF: plateletderived growth factor and TNFa: tumour necrosis factor alpha.)

The angiogenic balance, results from homeostasis between the factors involved in the activation phase and those involved in the maturation phase of angiogenesis. The receptor ALK1 plays a key role in inhibiting the

proliferation, migration and budding of endothelial cells in vitro, as well as neo-angiogenesis in vivo. The ligand for ALK1, BMP9 may thus be a key factor in the maturation phase of angiogenesis (Figure 3) and its presence in the blood suggests it plays a role in maintaining vascular quiescence in adults[29]. When the BMP9/ALK1/endogline pathway is disturbed, the quiescence is decreased, resulting in the dysregulation of this angiogenic balance, and thus a neo-activation of angiogenesis.

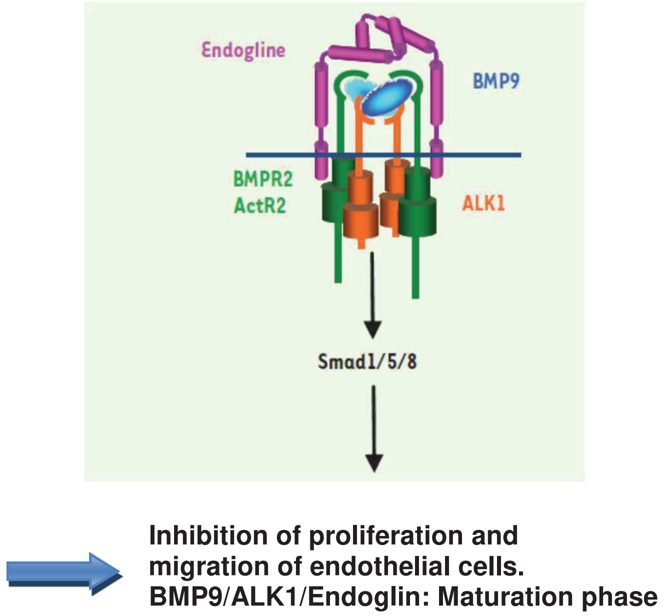

Figure 3: BMP9/ALK1/endoglin pathway (taken from S. Bailly[30])

BMP9 binds on to a heterocomplex composed of two ALK1 receptors and two type 2 receptors (BMPR2 or ActR2). The type 2 phosphoryl ALK1 receptor in turn phosphorylates the Smad1/5/8 transcription factors. Adding BMP9 results in the inhibition of the migration and proliferation of the endothelial cells, which suggests a role for this signaling pathway in the maturation phase of angiogenesis.

2.1.4 Angiogenesis in HHT

In HHT, the mechanisms leading to predisposition and formation of AVMs, the direct connections between arteries and veins, are yet to be determined. One proposed mechanism is defective arteriovenous differentiation, observed in *Eng* and *Alk1* null embryos that develop AVMs [31] but absent in the endothelial-targeted *Eng* (*Eng*-iKOe) and *Alk1* inducible knockout (*Alk1*-iKOe) mice [32]. Moreover, focal regression of capillaries leading to formation of AVMs has also been postulated in HHT, however, supporting data for this model are still lacking. Recently, it was shown that wound injury was necessary for development of AVMs in adult *Alk1*-iKOe [33] and *Eng*1-iKOe mice. In addition, intracerebral injection of an adenovirus expressing VEGF contributed to pathogenesis of cerebral AVMs in several transgenic *Eng* KO mice.

The recognized manifestations of HHT are all due to abnormalities of vascular structure. Lesions may be small (telangiectases) or large arteriovenous malformations (AVMs) (Figure 4).

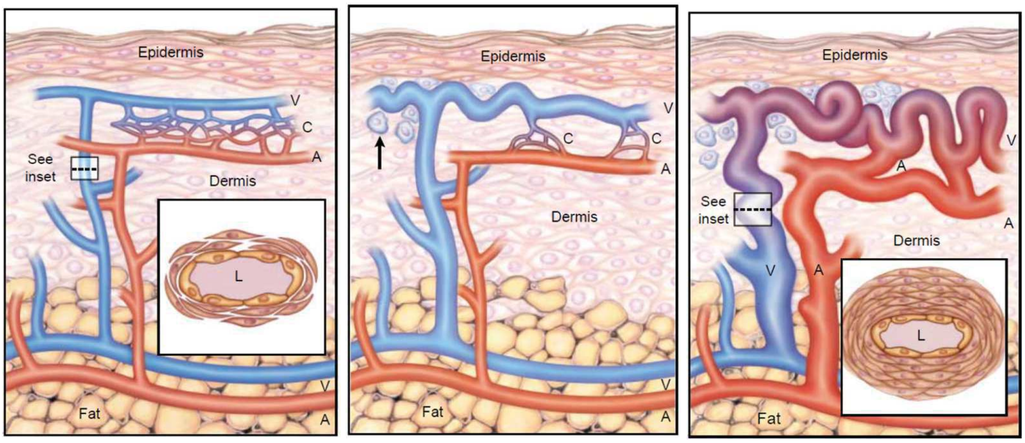

Figure 4: Evolution of a Cutaneous Telangiectasis in HHT (from Guttmacher et al[34])

In normal skin (left panel), arterioles (A) in the papillary dermis are connected to venules (V) through multiple capillaries (C). These vessels arise from larger arterioles and venules at the junction of the dermis and fat. The ultrastructure of a normal postcapillary venule (shown in cross section in the inset) includes the lumen (L), endothelial cells, and two to three layers of surrounding pericytes. In the earliest stage of cutaneous telangiectasia (middle panel), a single venule becomes dilated, but it is still connected to an arteriole through one or more capillaries. A perivascular lymphocytic infiltrate is apparent (arrow). In a fully developed cutaneous telangiectasis (right panel), the venule and its branches have become markedly dilated, elongated, and convoluted throughout the dermis. The connecting arterioles have also become dilated and communicate directly with the venules without intervening capillaries. The perivascular infiltrate is still present. The thickened wall of the dilated descending limb (shown in cross section in the inset) contains as many as 11 layers of smooth-muscle cells.

During recent years, the role of VEGF in HHT has emerged. Indeed, an over-activation of VEGF pathway leads to aberrant angiogenesis. Since 2006, it has been suggested by animal models and then by clinical reports that anti-VEGF therapy may be useful to treat HHT [35-37].
Figure 5 summarizes the angiogenesis disequilibrium in HHT.

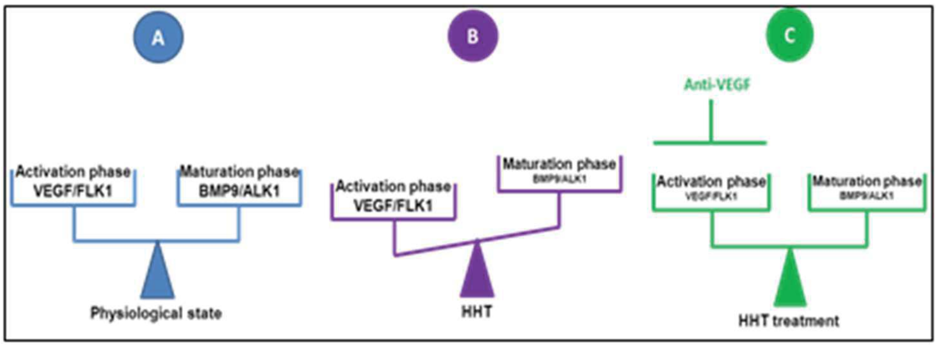

Figure 5: Angiogenic balance in HHT (taken from S. Bailly[30])

A. In healthy adults, angiogenesis is balanced between the activation and maturation phases, and the vascular network is quiescent.

B. In HHT patients, the BMP9/ALK1/ENG signaling pathway is deficient, which results in a decrease in the maturation phase and thus an angiogenic imbalance in favor of the activation phase (VEGF).

***C.* A new treatment approach in this disease** is thus to decrease the activation phase of angiogenesis using anti-VEGF drugs to restore the balance.

2.1.5 *Anti-angiogenic treatments in HHT*

2.1.5.1 Anti-VEGF treatment: bevacizumab

Based on physiopathological mechanism of an unbalanced angiogenesis in HHT, we intented to repurpose antiangiogenic treatments in HHT.

First, we evaluated bevacizumab (trade name Avastin®, Roche) efficacy and safety in HHT[37]. Bevacizumab is a humanized monoclonal antibody that binds to VEGF and therefore inhibits the stimulation of its receptors situated on the surface of the endothelial cells.
It is indicated in cancerology in association with anti-mitotic treatments for first or second line treatment for metastatic cancer (treatment of Breast Neoplasms Carcinoma, Non-Small-Cell Lung Carcinoma, Renal Cell Colorectal Neoplasms, Ovarian Neoplasms) and to treat age-related macular degeneration with intra-vitreal administration.

In HHT, 2 case reports published in 2006 and 2008 showed a spectacular improvement in HHT patients with severe liver involvement with additional cardiac involvement[35,36]. One of these patients had severe digestive bleedings which dramatically improved after treatment.

A first study using intravenous bevacizumab in HHT was conducted by our team between March 2009 and November 2012 (Metafore study) to study the efficacy of bevacizumab in the serious hepatic forms of the disease[37] (Eudract 2008-006755-44, NCT00843440). This study highlighted the efficacy of this treatment, not only on liver involvement, as shown by a decrease in the cardiac hyper flow secondary to liver vessel malformations, but also a significant decrease in the nosebleeds (Figure 6), which considerably improved the quality of life of the patients. No severe adverse events related to bevacizumab have been observed.

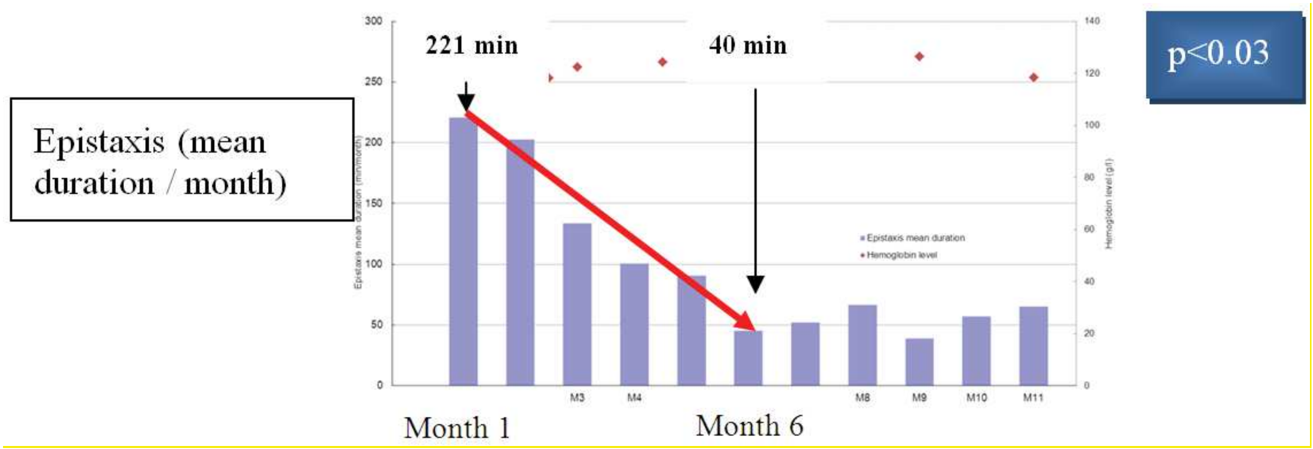

Figure 6: Significant decrease in nosebleeds after six IV infusions of bevacizumab.

Furthermore, many case reports showing dramatic improvement of HHT bleedings after bevacizumab treatment have been published[35,38-47].

The tolerance was very acceptable. High blood pressure was observed in 15% of patients with a good response to treatment and other side effects observed in HHT were asthenia, headaches, articular and muscular pain.

In HHT, we did not observe a higher risk of bleeding using bevacizumab treatment based on the mechanism of action, but on the contrary, we observed a dramatic improvement of nasal or digestive bleeding.

Orphan drug designation has been obtained in 2014 by Dr S. Dupuis-Girod for bevacizumab in HHT (EMA/3/14/1390).

A randomized multicenter phase III clinical trial to study bevacizumab efficiency and safety on severe bleedings is in progress, BABH study (NCT03227263).

Bevacizumab use is currently limited to severe forms of the disease due to the administration route, price and absence of market authorization. The company didn't plan to develop bevacizumab treatment in HHT.

For these reasons, it is of major importance to study other anti-angiogenic drugs, potentially efficient in HHT too.

2.1.5.2 Tyrosine kinase inhibitor and HHT

Tyrosine kinase inhibitors are anti-angiogenic molecules which are available orally and could therefore overcome the difficulties encountered with bevacizumab.
Tyrosine kinase inhibitors (Nintedanib and Pazopanib) are small molecules that target the intracellular kinase domain of the VEGF receptor (figure 7). The tyrosine kinase inhibitor Nintedanib targets growth factor receptors involved in angiogenesis. Most importantly it inhibits the platelet-derived growth factor receptor (PDGFR), fibroblast growth factor receptor (FGFR) and vascular endothelial growth factor receptor (VEGFR).

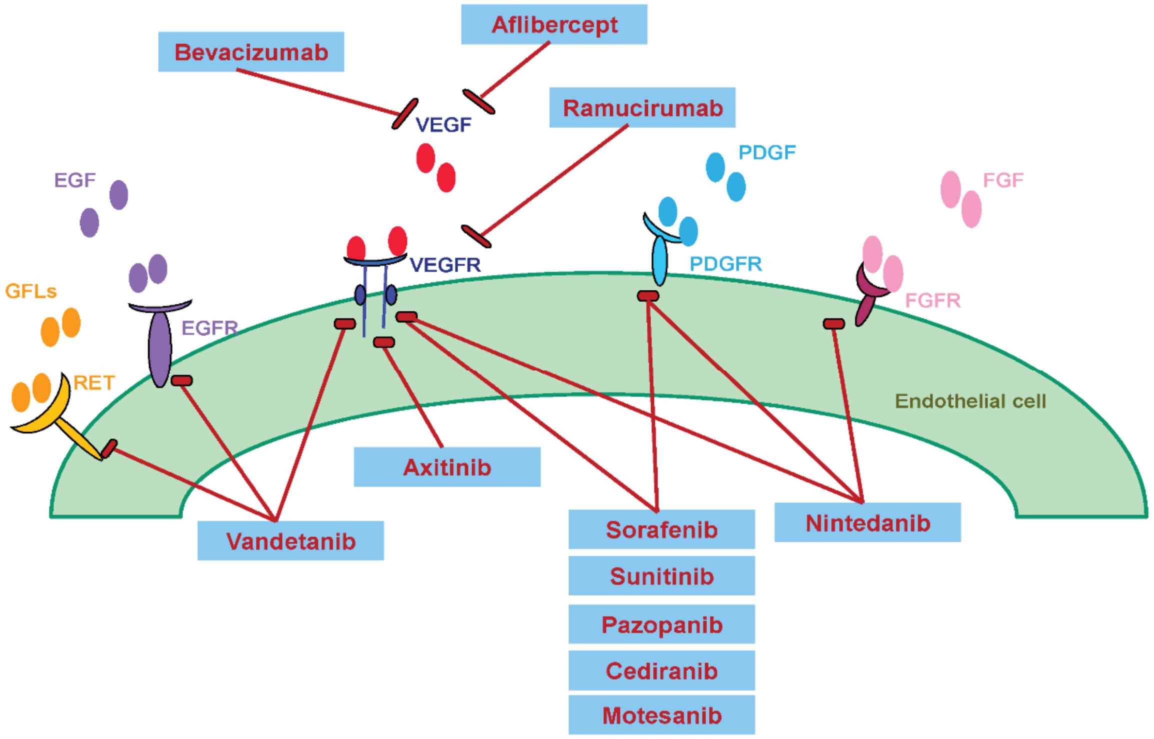

Figure 7: Angiogenesis and potential targets for treatments

The potential therapeutic effects of four antiangiogenic tyrosine-kinase inhibitors in the development of adult-onset AVMs in a murine model of HHT was evaluated [48]. They concluded that Sorafenib and a Pazopanib analog (GW771806) significantly improved hemoglobin level and gastro-intestinal bleeding whereas they were not effective in preventing wound-induced skin AVMs.

Nintedanib has been used in one HHT patient following the diagnosis of Insterstitial Pulmonary Fibrosis[49] with encouraging results. It was started at an oral dose of 150 mg two times a day, with the intent of preventing further progression of the pulmonary fibrosis. Before nintedanib treatment, this patient had experienced daily epistaxis of 6 to 15 min duration, and nosebleeds decreased to only one episode per month of <1 min. His Epistaxis Severity Score decreased from a moderate score of 5.5 to a mild value of 0.5. This improvement has been sustained while on nintedanib and there has been no worsening of his epistaxis in the last 12 months of follow-up.

Pazopanib, another tyrosine kinase inhibitor, has been tested at a dose of 50 mg/day in HHT by Faughnan et al.[50] and showed promising results in treatment of HHT related bleeding (NCT02204371). Five patients out of 7 showed more than 50% decrease in epistaxis duration and 2/7 showed more than 50% decrease in epistaxis severity, resulting in improvement of hemoglobin levels. This drug was initially developped by GlaxoSmithKline company. In 2015, GSK formed a consumer health joint venture with Novartis and unfortunately they decided to stop the study.

In a case report published in 2018[51], they describe a patient with HHT and refractory epistaxis to several endonasal procedure and bevacizumab treatment. Low doses of pazopanib were administered to the patient

to evaluate efficacy on severe epistaxis associated with anemia. First, the posology was 50 mg/day for 1 month, and it was increased to 100 mg/day afterwards. Epistaxis Severity Score decrease from 9.09 before the treatment to 5.76 after 1 month, 2.70 after 3 months and 1.41 after 1 year treatment. Hemoglobin levels over a five years period are represented in figure 8, an increase of hemoglobin rates is observed after pazopanib treatment introduction. No side effects were reported.

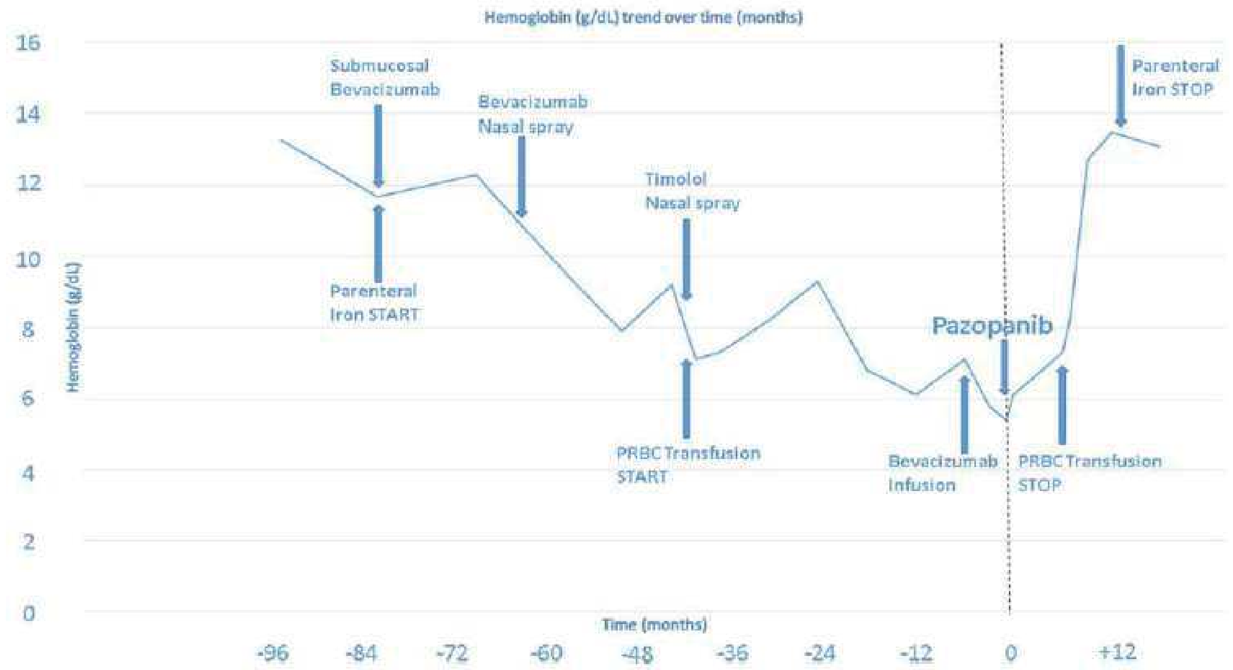

Figure 8: Case report, Hemoglobin (g/dL) trend over time (months)

2.2 Hypothesis of the study

To date, no treatment (surgical or medicated) has made it possible to significantly decrease nosebleeds – the most common complication of the disease, responsible for significant morbidity – in the medium to long term in patients with HHT. Moreover, surgical treatments are aggressive for the nasal mucosa and risk perforating the nasal septum. Bevacizumab use is currently limited to severe forms of the disease due to the administration route, price and absence of market authorization. Tyrosine kinase inhibitors are potentially interesting drugs to be use in HHT. Most of them have marketing authorization in oncology, but one, the nintedanib has obtained a market authorization in another rare disease idiopathic pulmonary fibrosis(IPF), in 2014 (OFEV®) and data on efficacy and safety are available in this rare disease. For this reason and because of published case report in HHT, we focused our attention on Nintedanib. The treatment tested is nintedanib, manufactured by Boehringer Ingelheim International GmbH and commercialized as OFEV®, 100 mg and 150 mg soft capsules.

Nintedanib targets growth factor receptors involved in angiogenesis. Most importantly it inhibits the platelet-derived growth factor receptor (PDGFR), fibroblast growth factor receptor (FGFR) and vascular endothelial growth factor receptor (VEGFR). Therefore, we hypothesize that nintedanib, acts by indirect inhibition of the VEGFR and should allow a reduction of epistaxis in HHT patient.

Moreover, nintedanib, is a suitable candidate because of its administration route (oral) and its safety profile (side effects with nintedanib can be managed in most patients).

According to case reports published, the epistaxis improvement was observed after 3 weeks of nintedanib treatment. In our experience with bevacizumab, the treatment was administered over 2.5 months (6 times 14-days apart) and improvement on epistaxis was observed 2 to 3 months after the beginning of the treatment. Based on these data, we decided to choose a similar duration and to treat patients during 12 consecutive weeks with nintedanib.

The treatment will be proposed to HHT patient with moderate to severe epistaxis. Duration of nose bleedings over the last 8 weeks of treatment will be compared to the duration over the 8 weeks before the treatment. After the end of the treatment, patients will have 12 weeks of follow up to evaluate efficacy and safety.

2.3 Justification of the methodological choices

The methodology retained for this efficacy study is a randomized trial which is the design presenting the highest level of evidence.

Criteria will be evaluated by comparing data recorded at the following visits:

- Inclusion: data collected for 8 weeks before treatment and during the inclusion visit (day -55 to day 0 included).
- End of treatment: Based on the assumption that response on angiogenesis is not immediate, the criteria evaluation period will start after 4 weeks of treatment up to the end of treatment including information recorded during the end of treatment visit (it will cover the last 8 weeks of treatment: day 29 to day 84 included).
- End of study: Based on the assumption that treatment could have a remanence effect, the criteria evaluation period will start 4 weeks after the end of treatment up to the end of study including information recorded during the end of study visit (it will cover the last 8 weeks follow up: day 113 to day 168 included).

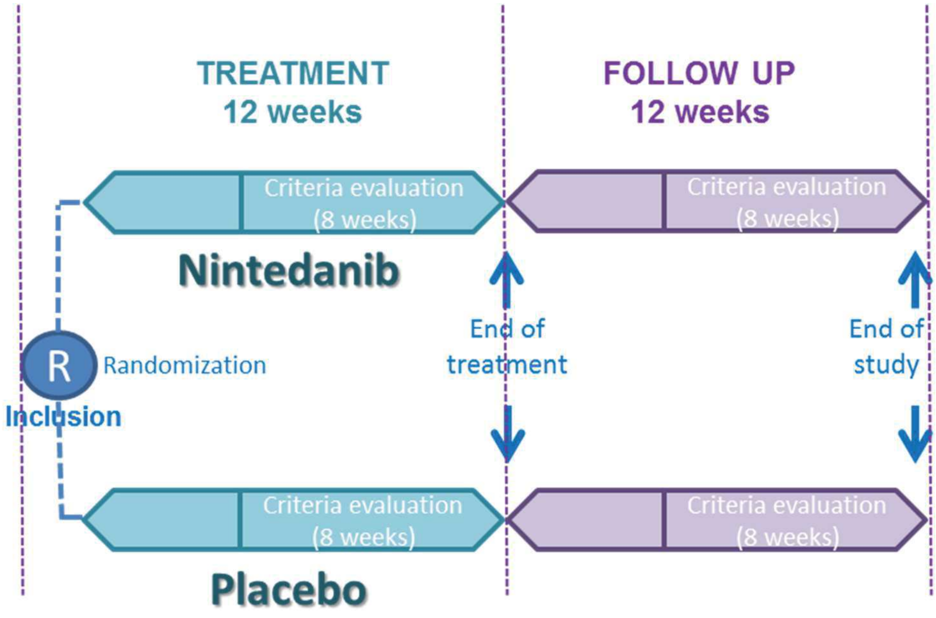

Figure 9: Study design

2.4 Target population

This study will involve adult patients suffering from HHT moderate to severe epistaxis which responsible for severe alterations in social functioning and quality of life. This parameter is evaluated with an Epistaxis Severity Score. It has been established that patients with epistaxis from moderate to severe, that is to say a score ESS> 4, have a poor quality of life[20,52].

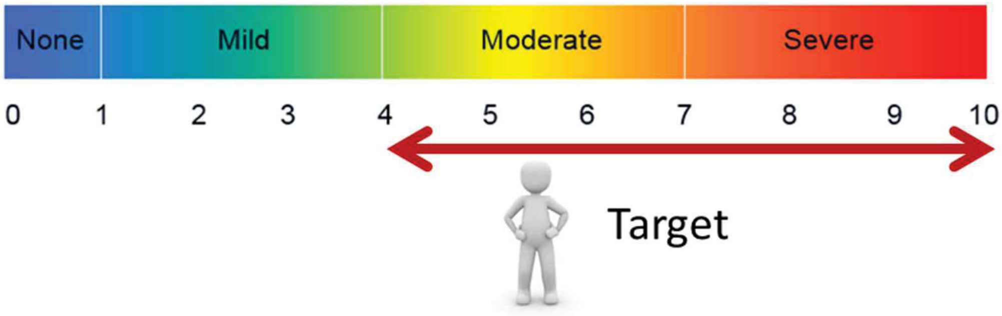

Figure 10: ESS score and Target population

2.5 Benefit-risk ratio

Side effects have been studied in previous studies. In pulmonary fibrosis (IPF), we summarized side effects and their frequency in recent studies[53-55]:

- Potential Risks:

The most frequently reported adverse reactions associated with the use of nintedanib included diarrhea, nausea and vomiting, abdominal pain, decreased appetite, weight decreased and hepatic enzyme increased. The safety of OFEV® was evaluated in over 1000 IPF patients with over 200 patients exposed to nintedanib for more than 2 years in clinical trials. Nintedanib was studied in three randomized, double-blind, placebo-controlled, 52-week trials. In the phase 2 and phase 3 trials, 723 patients with IPF received nintedanib 150 mg twice daily and 508 patients received placebo. The median duration of exposure was 10 months for patients treated with nintedanib and 11 months for patients treated with placebo. Subjects ranged in age from 42 to 89 years (median age of 67 years). Most patients were male (79%) and Caucasian (60%).

Adverse events led to premature treatment discontinuation in 20.6% of patients in the nintedanib group versus 15.0% of patients in the placebo group. The most frequently reported adverse event in patients treated with nintedanib was diarrhea, which was reported in 61.5% of patients in the nintedanib group versus 17.9% of patients in the placebo group and led to premature treatment discontinuation in 5.3% versus 0.2% of patients in these groups, respectively. The proportion of patients who had 1 serious adverse event was comparable between the nintedanib and placebo groups (30.0% and 30.1%, respectively).

Adverse events: Pooled data from the TOMORROW and INPULSIS® trials.

N (%)	Nintedanib 150 mg bid (n = 723)	Placebo (n = 508)
Any adverse event(s)	689 (95.3)	456 (89.8)
Most frequent adverse events[a]		
Diarrhoea	445 (61.5)	91 (17.9)
Nausea	176 (24.3)	36 (7.1)
Nasopharyngitis	93 (12.9)	79 (15.6)
Cough	93 (12.9)	75 (14.8)
Vomiting	85 (11.8)	15 (3.0)
Decreased appetite	81 (11.2)	24 (4.7)
Bronchitis	76 (10.5)	56 (11.0)
Progression of IPF[b]	68 (9.4)	72 (14.2)
Upper respiratory tract infection	65 (9.0)	55 (10.8)
Dyspnoea	55 (7.6)	59 (11.6)
Severe adverse event(s)	193 (26.7)	119 (23.4)
Serious adverse event(s)	217 (30.0)	153 (30.1)
Fatal adverse event(s)	38 (5.3)	43 (8.5)
Adverse event(s) leading to treatment discontinuation[c]	149 (20.6)	76 (15.0)
Diarrhoea	38 (5.3)	1 (0.2)
Nausea	17 (2.4)	0 (0.0)
Progression of IPF[a]	15 (2.1)	27 (5.3)
Decreased appetite	11 (1.5)	1 (0.2)
Weight decreased	8 (1.1)	1 (0.2)
Abdominal pain	7 (1.0)	1 (0.2)
Vomiting	7 (1.0)	1 (0.2)
Pneumonia	6 (0.8)	5 (1.0)

Treated set (patients treated with ≥1 dose of trial drug).
[a] Adverse events reported by >10% of patients in either treatment group.
[b] Corresponds to the MedDRA term 'IPF', which included disease worsening and IPF exacerbations.
[c] Adverse events leading to treatment discontinuation in ≥1% of patients in either treatment group by MedDRA preferred term.

Table 1: Adverse events in a pooled analysis of data from patients treated with nintedanib 150 mg twice daily or placebo in the TOMORROW and INPULSIS® trials (mean exposure 10.2 months for nintedanib and 10.8 months for placebo)[55].

- Protection against study risks:

  Patients will be informed about the risks. All adverse reaction will be carefully monitored regularly throughout the study. In case of apparition, the treatment could be interrupted according to investigator's brochure recommendations. Gastrointestinal disorders will be treated (diarrhea will be treated with adequate hydratation and anti-diarrheal medicinal product). A leaflet will be provided with the treatment, and it will include the behavior to have in case of adverse event. A participation card will be provided as well with emergency phone number.

Haemorrhage risk:

In the present study, our aim is to improve bleedings in HHT patients. Haemorrhage risks are detailed in Noth I et al [54]. They compared post-marketing surveillance data came from 6758 patients with IPF treated with nintedanib, two phase III INPULSIS trials, and epidemiological date from general population.

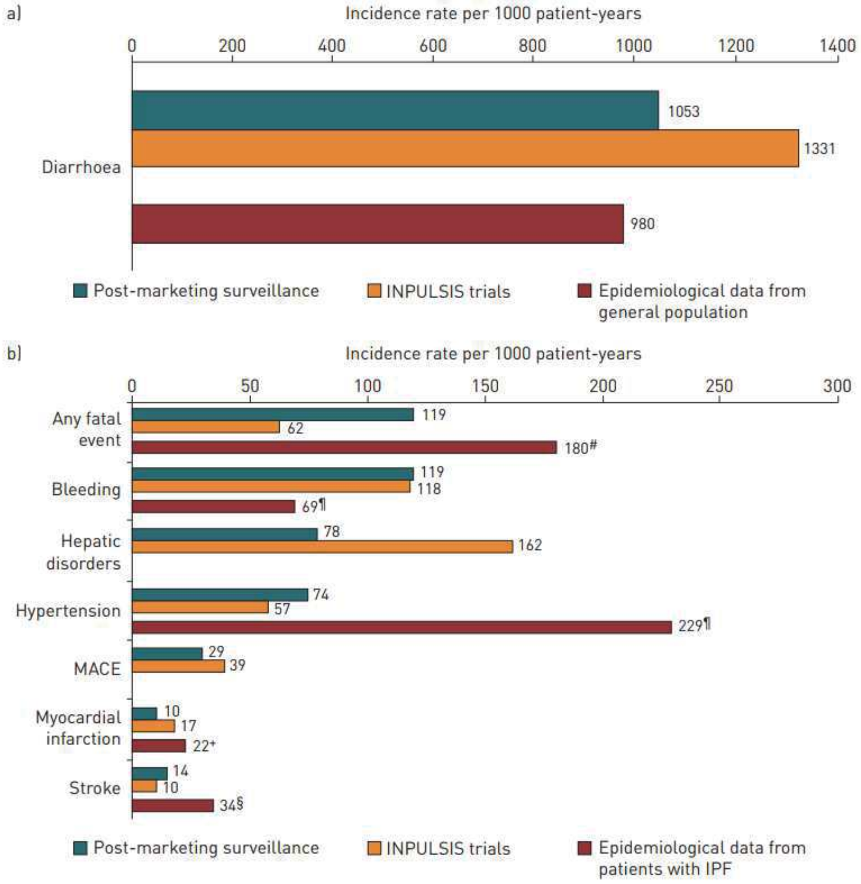

Figure 11: incidence of diarrhea and other adverse events of interest from Noth et al[54]

a) Incidence rates of diarrhea across populations.
b) Incidence rates of other adverse events of interest across populations. No data from epidemiological studies on hepatic disorders or major adverse cardiovascular events (MACE) were available from the published literature.

Based on its inhibition of the vascular endothelial growth factor receptor, nintedanib may increase the risk of bleeding. Patients at known risk of bleeding, including those treated with full-dose anticoagulants or high-dose antiplatelet therapy were excluded from clinical trials, and the US product label specifies that patients at known risk of bleeding should receive nintedanib only if the anticipated benefit outweighs the potential risk. The incidence of bleeding events in the post-marketing surveillance data was similar to that in INPULSIS (119 versus 118 per 1000 patient-years). Most bleeding events were not serious; the most frequent were epistaxis, contusion and rectal hemorrhage. The rate of bleeding events in patients with IPF based on healthcare claim data was lower than in the post-marketing surveillance data (figure 1b). This was not unexpected, as bleeding events that are not severe enough for a patient to seek medical attention (e.g. epistaxis) are generally not captured in claim-based data. Not surprisingly, known use of anticoagulants was higher in patients with bleeding than non-bleeding adverse events. Of 324 cases of bleeding in the post-marketing surveillance data, concomitant use of anticoagulants was reported in 37.7% and was unknown in 34.3% of cases. Of 4739 adverse events that were not bleeding, concomitant use of anticoagulants was reported in 25.4% and was unknown in 46.3% of cases.

They concluded that, in Nintedanib trials for Idiopathic Pulmonary Fibrosis [56] [57] reassuringly there was no increased signal regarding cardiovascular or bleeding complications with nintedanib therapy[58].

With bevacizumab, the hemorrhagic risk was similar and the available data are reassuring. A recent meta-analysis[59] revealed that bevacizumab does not significantly increase the risk of intracranial hemorrhage in solid tumor patients with brain metastases, and it has provided evidence indicating that brain metastases patients with a low incidence of intracranial hemorrhage, such as those with advanced/metastatic breast cancer, non-

squamous non-small cell lung cancer, renal and colorectal cancer, may not be generally excluded from bevacizumab therapy or trials.

In our previous study using bevacizumab in HHT[37], the drug was administered intravenously (5 mg/kg) in patients with severe liver arterio-venous malformations and we did not observe a higher risk of bleeding but on the contrary a dramatic improvement of nasal and/or digestive bleeding was observed. Only patients with cerebral AVMs were excluded from this study.

In order to conduct the study as safe as possible regarding hemorrhage risks, we decided:

1. To exclude patients with cerebral arteriovenous malformation (about 10% of patients). All patients will have a cerebral MRI before inclusion, if not done within 5 years.
2. To exclude patients with active gastro-intestinal bleeding or GI ulcers within 12 months prior to inclusion.
3. To exclude patients with hemoptysis or hematuria within 12 weeks prior to inclusion.
4. Not to exclude patients without PAVMs on a CT scan (<5 years) or patients with treated PAVMs (vascular treatment according to recommendations) or patients with small PAVM (<3mm) not accessible to a vascular treatment on CT scan within 1 year.
5. Not to exclude patients with hepatic AVM. The presence of telangiectasia or hepatic AVM is frequent (>80%) and in more than 80% of cases patients are asymptomatic; only 5 to 10% of patients have a severe liver involvement leading to a high-output cardiac failure, but without hemorrhagic risk.

Arterial thromboembolic events:

Due to physiological mechanism caution should be used when treating patients at higher cardiovascular risk including known coronary artery disease. Patients with known coronary artery disease or with a recent history of myocardial infarction or stroke will not be included.

In INPULSIS-1 study myocardial infarction was reported in 5 patients in the nintedanib group (1.6%) and 1 patient in the placebo group (0.5%) and in INPULSIS-2 study, myocardial infarction was reported in 5 patients in the nintedanib group (1.5%) and 1 patient in the placebo group (0.5%).

In these two studies, no venous thrombosis or other arterial thrombosis have been reported.

- Potential Benefits:

  The expected benefits are: to decrease epistaxis duration, to improve clinical condition of the patient with moderate and severe nosebleeds, to improve quality of life, to decrease the number of hospitalizations for red blood cells transfusions and/or iron infusions, and to increase in hemoglobin and serum ferritin levels.

The benefit risk balance appears to be favorable.

2.6 Expected impacts

The expected impact is enormous. Indeed, the most apparent expression of the disorder is the occurrence of spontaneous, repeated epistaxis. These epistaxis can be severe and disabling; they are often the cause of chronic anemia, and can require continuous management including repeated hospitalizations for iron supplementation and blood transfusions. Furthermore, epistaxis are responsible for major social consequences in HHT patients. Currently no treatment decrease significantly nosebleeds. Surgical treatments are aggressive for the nasal mucosa and risk perforating the nasal septum. No local drug treatment could show efficacy. Bevacizumab use is currently limited to severe forms of the disease due to the administration route, price and absence of market authorization.

An oral treatment of nintedanib, if well tolerated, could be a therapeutic option.

3 STUDY OBJECTIVES

3.1 Primary objective

The primary objective is to evaluate efficacy, at the end of the treatment period, on epistaxis duration of nintedanib treatment per os (300 mg/day for 12 weeks) versus placebo in patients with HHT complicated by moderate to severe epistaxis.

3.2 Secondary objective

Secondary objectives are:

1. To evaluate nintedanib safety in HHT patients.
2. To evaluate efficacy of nintedanib treatment on epistaxis (duration, frequency and severity).
3. To evaluate efficacy of nintedanib treatment on other clinical criteria: Quality of life: SF36, number of red blood cell transfusions and number of iron infusions.
4. To evaluate efficacy of nintedanib treatment on biological criteria: hemoglobin and ferritin levels.

4 STUDY DESIGN

4.1 Type of study

This is a multicenter, randomized versus placebo (ratio 1:1) study carried out in a double blind setting.

4.2 Randomization method

The randomization process will be centralized. Allocation of a randomization arm to a patient included will be made by IWRS (Interactive Web Response System) on the basis of a unique randomization list for all investigation centers.

The list of randomization will be pre-established with neutral code (1 and 2), by the Pôle de Santé publique at the Hospices Civils de Lyon – Clinical Research Unit.

The software Ennov clinical will be used for the data management of this study.
During the inclusion visit, after verification of inclusion criteria, the investigator connects to the platform to include the patients in an eCRF that will assign an identification number. When inclusion is validated, the patient is randomized and a therapeutic unit is allocated. The treatment is then dispensed by pharmacy of the Hospital Center.

This will be a double blind study in which neither the patient nor the investigator will be aware of the nature of the treatment administered so as to annul any bias in the follow-up and measurements.

Unblinding request is made through the Anti-Poison Center in Lyon 24h/24h. The written detailed procedures for lifting the blind will be given to the investigators and the Anti-Poison Center in Lyon.
All requests for unblinding must be justified. Most often, study drug discontinuation and knowledge of the possible treatment assignments are sufficient to treat a study patient who presents with an emergency condition. When the investigator asks for unblinding, he/she must provide patient identifying information, date and reason for unblinding. The investigator will be informed on the details of the drug treatment. The monitor will be informed too.
Study drug must be discontinued after an unblinding procedure.

4.3 Endpoints

4.3.1 Primary endpoint

Proportion of participants reporting a response at the end of the treatment. A response is defined by a reduction of at least 50% on epistaxis monthly mean duration during the last 8 weeks of treatment (day 29 to day 84

included) as compared to the 8 weeks before treatment (day -55 to day 0 included). Monthly mean duration is defined as total duration recorded X (28/number of day available for the reporting period). This criterion will be assessed on the basis of monitoring of epistaxis grids filled in by the patients (collected at each visit or filled in online).

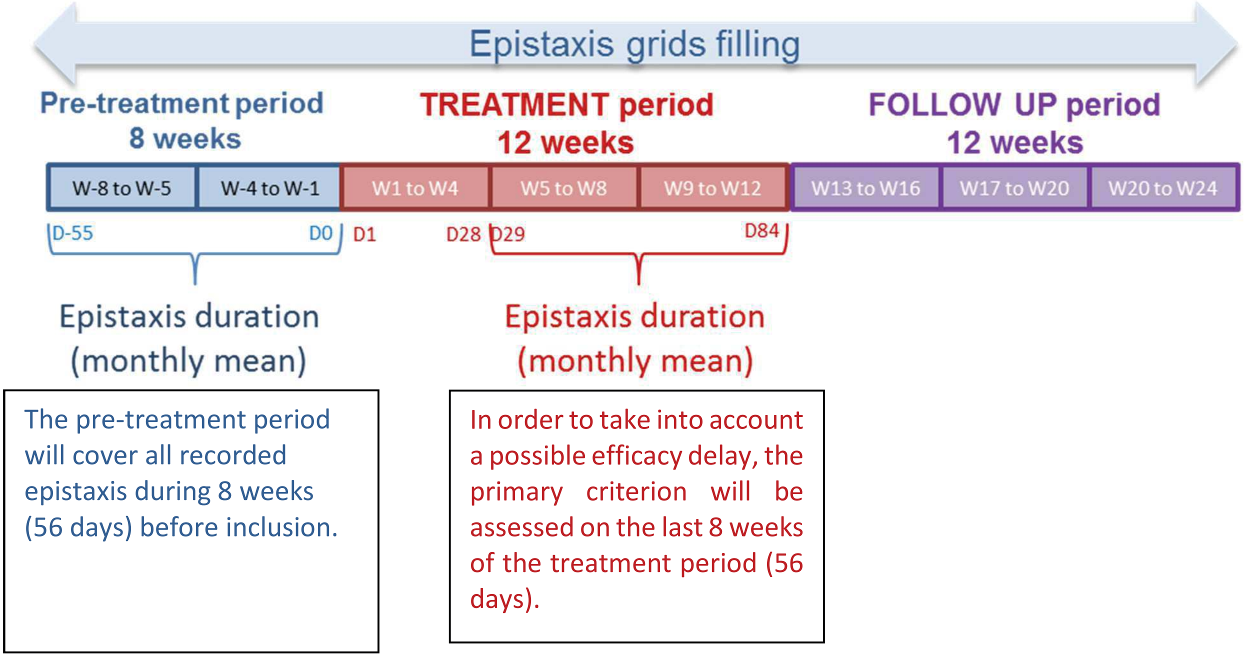

Figure 12: Primary endpoint evaluation

4.3.2 Secondary endpoints

1. All adverse events and severe adverse events observed during the study will be collected.
2. Epistaxis will be assessed through

– Proportion of patients reporting a response at the end of follow-up. A response is defined by a reduction of at least 50% on epistaxis monthly mean duration during the last 8 weeks of follow-up (day 113 to day 168 included) as compared to the 8 weeks before treatment (day-55 to day 0 included). Assessment by epistaxis grids filled in by patients (collected at each visit or filled in online).

– Nosebleeds monthly mean duration (continuous variable) will be computed during the 8 weeks before treatment (day -55 to day 0 included), during the last 8 weeks of the treatment period (day 29 to day 84 included) and during the last 8 weeks of the follow-up period (day 113 to day 168 included). Differences from baseline will be assessed. Assessment by epistaxis grids filled in by patients (collected at each visit or completed online).

– Nosebleeds monthly mean duration (continuous variable) will be computed all over the study period using 4 weeks periods. Assessment by epistaxis grids completed by patients (collected at each visit or filled in online).

– Nosebleeds frequency (considered as continuous variable) will be computed during the 8 weeks before treatment (day -55 to day 0 included), during the last 8 weeks of the treatment period (day 29 to day 84 included) and during the last 8 weeks of the follow-up (day 113 to day 168 included). Differences from baseline will be assessed. Assessment by epistaxis grids filled in by patients (collected at each visit or completed online).

– Epistaxis score = ESS (continuous variable) will be calculated from questionnaire filled by patients at inclusion visit, at the end of the treatment period and at the end of the follow-up.

3. Other clinical criteria :

– Quality of life = SF36 score based on questionnaire filled in by patients at inclusion visit (baseline), at the end of the treatment and end of the follow up will be calculated.

- Number of red blood cell transfusions (discrete variable) is collected for 8 weeks before treatment (day -55 to day 0 included), during the last 8 weeks of the treatment period (day 29 to day 84 included) and during the last 8 weeks of the follow-up period (day 113 to day 168 included).
- Number of iron infusions (discrete variable) is collected for 8 weeks before treatment (day -55 to day 0 included), during the last 8 weeks of the treatment period (day 29 to day 84 included) and during the last 8 weeks of the follow-up period (day 113 to day 168 included).

4. Biological criteria:

- Hemoglobin level (continuous variable) will be measured at inclusion, at the end of the treatment visit and at the end of follow-up visit.
- Ferritin level (continuous variable) will be measured at inclusion visit, at the end of the treatment visit and at the end of follow-up visit.

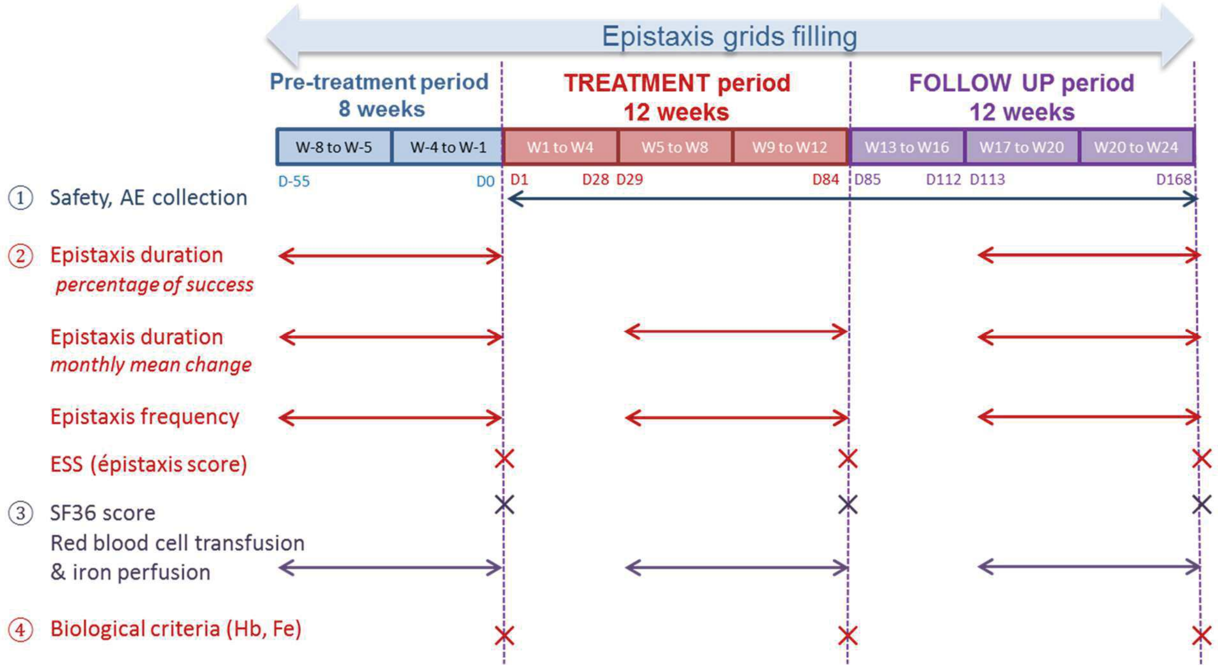

Figure 13: Secondary endpoints evaluations

5 ELIGIBILITY CRITERIA

5.1 Inclusion criteria

- Age > 18 years old
- Patients who have given their free informed and signed consent
- Patients affiliated to a social security scheme or similar
- Patients monitored for clinically confirmed HHT and/or with molecular biology confirmation
- Patient with an Epistaxis Severity Score (ESS) > 4 (target population of patients with important consequences on quality of life)

5.2 Non-inclusion criteria

- Pregnant woman or woman of child bearing potential not using two effective methods of birth control (one barrier and one highly effective non-barrier) for at least 1 month prior to trial and/or committing to using it until 3 months after the end of treatment.
- Woman who are breast feeding.
- Patient who are protected adults under the terms of the law (French Public Health Code).
- Participation in another interventional clinical trial which may interfere with the proposed trial (judgment of the investigator).
- Clinical evidence of active infection.
- (AST, ALT > 1,5 fold upper limit of normal (ULN) and/or Bilirubin > 1,5 fold upper limit of normal (ULN).
- Severe renal impairment (Creat Clearance <30 mL/min) estimated by the Cockcroft-Gault equation.
- Presence of non-treated pulmonary arteriovenous malformations accessible to a treatment on CT scan within 5 years.
- Patients with hemoptysis or hematuria within 12 weeks prior to inclusion.
- Patients with active gastro-intestinal (GI) bleeding or GI ulcers within 12 months prior to inclusion.
- Presence of cerebral arteriovenous malformation on MRI done within 5 years prior inclusion.
- Patients who require full-dose therapeutic anticoagulation (e.g. vitamin K antagonist or heparin, dabigatran) or high dose antiplatelet therapy, patients under anticoagulation with rivaroxaban, apixaban and epixaban.
- Patients with P-glycoprotein (P-gp) substrates/inducers/inhibitors (e.g.: ketoconazole, erythromycin, cyclosporine, rifampicin, carbamazepine, phenytoin, and St. John's Wort).
- Patients with known coronary artery disease or recent history of myocardial infarction (within 1 year).
- Known inherited predisposition to thrombosis or thrombotic events (including stroke and transient ischemic attack, excluded superficial venous thrombosis) within 12 months prior to inclusion.
- Patients with QTc prolongation (on ECG, less than 3 months).
- Hypersensitivity to nintedanib, peanut or soya, or to any of the excipients.
- Patient who incompletely filled in epistaxis grids within 8 weeks prior to inclusion.
- Patient who have received intravenous bevacizumab within 6 months prior to inclusion.
- Patient who had surgery (including ENT surgery) within 12 weeks prior to inclusion.
- Unhealed wound.
- Planned major surgery within the next 3 months, including liver transplantation, major abdominal or intestinal surgery.

5.3 *Criteria for premature termination*

If a patient is included and randomized and has not taken any treatment he(she) will be withdrawn from the study and replaced.

If the treatment is prematurely or temporary stopped by the patient, due to complications, adverse events, adverse reaction, introduction of a forbidden associated treatment or logistical reasons, the patient will not be replaced and will not be excluded from the study but will be followed-up for the 24 weeks after the beginning of the treatment as planned for the study.

In case of intercurrent events, the treatment can be prematurely or temporary stopped or delayed. It is up to the investigator to make the decision in the interest of the patient, on the basis of the balance between the risk of a new event and the risk of aggravation of the disease.

5.4 Recruitment methods and feasibility

Aspects to insure the recruitment for the feasibility of the project

1. Network coordinated by the reference center (over 2000 patients followed regularly). The cohort of patients followed for HHT at the Lyon reference center is around 1,000 patients. At the national level, more than 2,000 patient files are currently included in the database. Of these patients, at least 25% present with nosebleeds that justify ENT management.
2. Previous studies conducted by the Reference center, including Metafore published in 2012, Ellipse published in 2014, Alegori published in 2016, Tempo (NCT02484716) on going publication. And ongoing studies: TACRO (NCT 03152019) and BABH (NCT 03227263).
3. The support of the patients association (AMRO) and the high expectations of patients.

6 EXPERIMENTAL TREATMENTS

6.1 Study treatment

- **Identification**

The treatment tested is nintedanib, manufactured by Boehringer Ingelheim International GmbH and commercialized as OFEV®, 100 mg and 150 mg soft capsules, containing respectively 100 mg and 150 mg of nintedanib as esilate.

Ofev Marketing authorization numbers are EU/1/14/979/001, EU/1/14/979/002, EU/1/14/979/003, EU/1/14/979/004. Date of first authorization: 15 January 2015

Nintedanib is provided as soft gelatin capsules (150 mg: lilac, 100 mg: peach) containing a suspension of milled active as salt. The capsule fill is composed of medium chain triglycerides, hard fat and lecithin (soya) (E322) in addition to the drug substance. The capsule shell is composed with gelatin, glycerol (85%), titanium dioxide (E171), iron oxide red (E172) and iron oxide yellow (E172).

- **Administration**

According to marketed authorization of Ofev for the treatment of Idiopathic Pulmonary Fibrosis, the recommended dose is administered for the study, i.e. 150 mg nintedanib twice daily administered approximately 12 hours apparts.

If a dose is missed, administration should resume at the next scheduled time at the recommended dose. If a dose is missed the patient should not take an additional dose. The recommended maximum daily dose of 300 mg should not be exceeded.

- **Dose adjustment**

In addition to symptomatic treatment if applicable, the management of adverse reactions could include dose reduction and temporary interruption until the specific adverse reaction has resolved to level that allow continuation of therapy. Treatment may be resumed at full dose (150 mg twice daily) or at reduced dose (100 mg twice daily) according to investigator judgment. In case of reduced dose prescription, it will be maintained until the end of the study treatment (without dose re-escalation). If a patient does not tolerated 100 mg twice daily, treatment should be discontinued.

In case of interruptions due to aspartate aminotransferase (AST) or alanine aminotransferase (ALT) elevations >3x upper limit of normal (ULN), once transaminases have returned to baseline values, treatment may be reintroduced at a reduced dose (100 mg twice daily) which subsequently may be increased to the full dose (150 mg twice daily).

- **Precautions for use**

The precautions of the treatment are described in the Summary of Product Characteristics (SPC) of OFEV.
The capsules should be taken with food, swallowed whole with water, and should not be chewed or crushed.

- **Overdose**

There is no specific antidote or treatment for nintedanib overdose. In case of overdose, treatment should be interrupted and general supportive measures initiated as appropriate.

6.2 Treatment of comparison

Placebo is provided as soft gelatin capsules (150 mg placebo: lilac, 100 mg placebo: peach) containing a suspension of titanium dioxide as drug substance substitute. The capsule fill is composed of medium chain triglycerides, hard fat and lecithin (soya) (E322) in addition to the titanium dioxide. The capsule shell is composed with gelatin, glycerol (85%), titanium dioxide (E171), iron oxide red (E172) and iron oxide yellow (E172).
Placebo soft capsules are manufactured by Boehringer Ingelheim International GmbH, the final product is strictly identical as the study treatment.

6.3 Product circuits

- **Manufacturing**

Manufacturing of capsules (150 mg and its placebo + 100 mg and its placebo) will be performed by Boehringer Ingelheim in conformity with current GMP guidelines. The order will be sent by the principal investigator as soon as he receives authorization from competent authorities (ANSM) and favorable opinion from ethic committee.

- **Packaging and labelling**

For the study, capsules will be packaged in child resistant high density polyethylene (HDPE) bottles, with an appropriate labeling. These operations will be performed by Boehringer Ingelheim in conformity with current clinical trial regulation..
Each bottle will contain 30 capsules, that is to say 15 days of treatment. For each patient the treatment kit is composed of 6 bottles.

- **Management of the products**

Boehringer Ingelheim will deliver the amount needed of products to the coordinator Pharmacy at the Hospices Civils de Lyon. Products are stored in coordinator Pharmacy premises who will supply the pharmacies in the centers participating in the study.

- **Dispensing the products**

The doctor investigator prescribes nintedanib or placebo on the specific prescription sheet for the study. The information provided by the doctor will be:

- Date of the prescription
- Surname, forenames and date of birth of the patient using a patient sticker
- The patient’s identification number
- The randomization code
- The treatment code allocated

The original of the prescription will be taken to the hospital pharmacy of the Hospital Center. The product allocated is then dispensed. One bottle of 30 capsules (the two first visits) or two bottles of 30 capsules (i.e. 60 capsules the following visits) are delivered to each patient; a specific leaflet is included with recommendations.

All traceability of the treatments used will be consigned to the prescription register specific to the trial at the Hospital Pharmacy at the Hospital Center.

- **Storage**

The shelf life of the product is 36 months. Bottles should be stored at room temperature, not above 30°C.

- **Return and destruction of unused products**

Bottles containing unused capsules are brought back by the patient and will be returned to the pharmacy at the hospital center. Remaining capsules will be counted and conserved for the monitoring visits and destroyed in accordance with the procedures in force after sponsor authorization.

6.4 Blinding

- **Organization**

The randomization process will be centralized. Allocation of a randomization arm to a patient included will be made by IWRS (Interactive Web Response System), on the basis of a unique randomization list for all investigation centers.

The list of randomization will be pre-established, by the "Pôle de Santé publique" at the Hospices Civils de Lyon – Clinical Research Unit.

Clinsight (Ennov clinical) software will be used to manage this study.
During the inclusion visit, after verifying the inclusion criteria, the investigator connects to the platform to create the patients in the eCRF. This will assign an identification number. When inclusion is validated, the patient is randomized and a treatment code is allocated by the system. The treatment is then dispensed by the pharmacy at the Hospital Center.
This will be a double blind study in which neither the patient nor the investigator will be aware of the nature of the treatment administered so as to annul any bias in the follow-up and measurements.

- **Unblinding**

Should a serious adverse event occur, the main investigator, the co-investigators or the sponsor may request that the Anti-Poison Center in Lyon lift the blind 24h/24h (the phone number is written on the clinical trial participation card). The written detailed procedures for lifting the blind will be given to the investigators and the Anti-Poison Center in Lyon.

All requests for unblinding must be justified. Most often, study drug discontinuation and knowledge of the possible treatment assignments are enough to treat a study patient who presents with an emergency condition. When the investigator asks for unblinding, he/she must provide patient identifying information, the date, and the reason for unblinding. The investigator will be informed of the details of the drug treatment.

At the end of the study, unblinding is carried out by the data management when the database is locked.

6.5 Authorized and forbidden associated treatments

Patients receiving full-dose therapeutic anticoagulation (e.g. vitamin K antagonist or heparin, dabigatran) or high dose antiplatelet therapy or patients under anticoagulation with rivaroxaban, apixaban and epixaban will not be included in this trial.Patients with P-glycoprotein (P-gp) substrates/inducers/inhibitors (e.g.: ketoconazole, erythromycin, cyclosporine, rifampicin, carbamazepine, phenytoin, and St. John's Wort) will not be included in this study.

Indeed, nintedanib is a substrate of P-gp. Co-administration with the potent P-gp inhibitor ketoconazole increased exposure to nintedanib 1.61-fold based on AUC and 1.83-fold based on Cmax in a dedicated drug-

drug interaction study. In a drug-drug interaction study with the potent P-gp inducer rifampicin, exposure to nintedanib decreased to 50.3% based on AUC and to 60.3% based on Cmax upon co-administration with rifampicin compared to administration of nintedanib alone.

If coadministered with nintedanib, potent P-gp inhibitors (e.g. ketoconazole, erythromycin or cyclosporine) may increase exposure to nintedanib.

Potent P-gp inducers (e.g. rifampicin, carbamazepine, phenytoin, and St. John's Wort) may decrease exposure to nintedanib.

If one of these treatments should be introduced during the patient's participation in the study, the treatment under study will be stopped.

7 GENERAL ORGANIZATION

7.1 Study calendar

Duration of the inclusion period: 30 months
Length of the participation of each patient: 24 weeks, *i.e. about 6 months* (from 158 to 178 days).
Total duration of the study: 36 months
Start of inclusions: june 2020

As soon as the first inclusion, the sponsor must inform the ethics committee and competent authorities without delay, of the effective start date of the study (effective start date = date of the signature of consent by the first person participating in study).
The end date of the study will be sent by the sponsor to the ethics committee and the ANSM for cat. 1 studies within a delay of 90 days. The end date of the study corresponds to the end of the participation of the last person participating in the study, or if applicable, to the end defined in the protocol.

7.2 General diagram and summary table

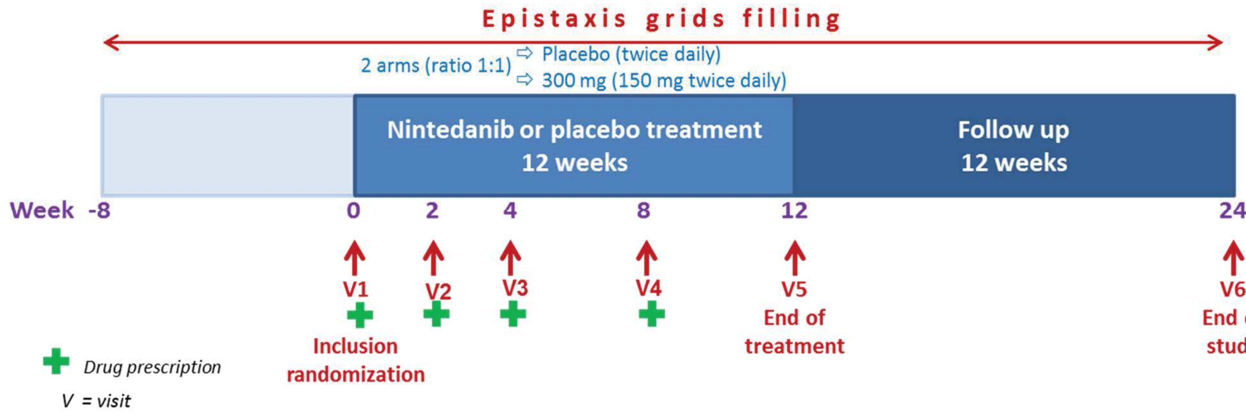

Figure 14: General diagram of the study

Day (D) - Week (W)	D-84 to D-56 (=W-12 to W-8)	D0	D14 (=2W) +/- 1 day	D28 (=4W) +/- 1 day	D56 (=8W) +/- 2 days	D84 (=12W) (D84 to D94)	D168 (=24W) (D158 to D178)
Visit	Standard follow-up	V1 Inclusion Randomisation	V2 Consultation	V3 Consultation	V4 consultation	V5 consultation end of treatment	V6 consultation end of study
Information	X	X					
Informed Consent		X					
inclusion/exclusion criteria	X	X					
Randomization		X					
Dispensing treatments		X	X	X	X		
Treatment (Day 1 to day 84)		↔	↔	↔	↔		
Dose adjustment			X	X	X		
Compliance			X	X	X	X	
Blood transfusions collection		X	X	X	X	X	X
Iron injection collection		X	X	X	X	X	X
Concomitants treatments	X	X	X	X	X	X	X
AE & SAE collection		X	X	X	X	X	X
Epistaxis grids (2)	↔	↔	↔	↔	↔	↔	↔
ENT examination		X				X	
Clinical examination		X	X	X	X	X	X
Blood pressure / heart rate		X	X	X	X	X	X
Pregnancy Test (βHCG dosage)		X	X	X	X	X*	X
SF36 QoL questionnaire		X				X	X
ESS questionnaire		X				X	X
Biology (NFS, hepatic function, complete iono, ferritin)		X	X	X	X	X	X

* delivery of two urine pregnancy tests for monthly check (16w & 20w)

Table 2: synopsis of the study per patient

7.3 *Conduct of the study*

7.3.1 *Screening –Pre-inclusion*

The patients likely to participate in the study will be identified from a standard consultation in the reference center or skill center for HHT or during an ENT consultation.

The physician will inform the patient about the study. All explanations that are necessary for the clear understanding of the study will be given to the patient, as well as an information letter explaining the objectives and the conduct of the study. The patient will be given the required period of reflection to decide on their participation in the study.

The doctor will ask for a daily accounting of epistaxis duration, if it is not done usually. Epistaxis grids will be given to the patient, they have to be completed for at least 8 weeks before inclusion.

The patient will be contacted afterwards in order to check epistaxis grids filling. If the patient agrees to participate, a date is scheduled for the first visit.

7.3.2 *Inclusion visit/Randomization (day 0)*

- The patient will be seen in HHT center for a medical consultation.
- The investigator will once inform the patient about the study and answer the questions.
- The ESS questionnaire (Appendix 2) will be filled in by patient.
- The inclusion and exclusion criteria will be verified. If the patient agrees to participate, the consent form will be signed, the volunteer and the investigator sign (name and surname clearly visible) and date two examples of the consent form. One is conserved by the patient; the other is conserved in the study site master file. The investigator connects to the Ennov's platform, include the patient, an identification number is attributed.
- The patient will have a clinical examination, including blood pressure and heart rate; an ECG (if not done in the previous 3 months); body weight and size.
- Any concomitant treatments will be noted, included iron infusions (refer to §6.5 for precautions to be used for certain associated treatments).
- The nose bleeds grids filled in by the patient for at least the last 8 weeks will be collected.

- The examination will be completed by a set of biological blood samples (3 x 5 mL tubes) for NFS (including hemoglobin), complete ionogram (including creatinin), serum ferritin and hepatic function (including liver enzymes, GGT, ALT & AST, PAL, bilirubin).
- For female patients of child-bearing age, a supplementary blood tube is sampled, for βHCG dosage. The investigator will inform these patients that there is no data available in pregnant women and they should use two effective methods of birth control: reliable non-barrier contraception (hormonal contraceptive, implant, intra-uterine device) combined with a barrier contraceptive (condom) during the treatment and for at least 3 months after the end of the treatment. For male, investigator will inform patient about the necessity to use contraceptive (condom) during the study.
- If all the criteria are validated, **the patient will be randomized**. The investigator connects to Ennov's platform, a randomization code will be assigned to the patient and the treatment number will be allocated.
- The number of transfusions (red blood cell units) for the last 8 weeks will be collected.
- An ENT consultation will be organized with a nasal examination to evaluate nasal lesions before treatment.
- A SF36 quality of life questionnaire (Appendix 3) will be filled in by the patient (online or on paper sheet).
- The specific prescription sheet will be filled in for drug dispensation (150 mg capsules) by the pharmacist at the hospital. A sufficient amount will be dispensed for 2 weeks of treatments (+1 day), i.e. 30 capsules (1 bottle). A leaflet will be given to patient with the drug; it includes precautions for use and recommendations in case of adverse events. It will be clear to the patient that she/he should keep all the packages and bring them back to the next visit. The treatment will be start by the patient the next morning at day 1. The patient must tick each drug intake in the table provided for this purpose on the epistaxis grids.
- A clinical trial participation card will be given to the patient. It will include phone numbers if needed and in case of emergency.
- A calendar will be given to the patient with dates for next visits.
- Epistaxis grids will be given to the patient to record all episodes of nose bleed and their duration if not completed on line.

Note: a personal identification number will be given to the patient to connect to Ennov Clinical platform. Questionnaires ESS and SF36, and epistaxis grids can be then filled in, the day of the visit, on line if convenient, otherwise specific paper sheet will be used.

If the criteria are not validated, the patient may be asked to return at a later date if the inclusion criteria can be normalized.

7.3.3 Follow-up visits

- **Visit at 2 weeks, V2 (D14 +/- 1 day)**

The patients will be seen in consultation, in the Reference center or skill center for HHT to monitor liver enzyme and evaluate bleedings.

The following examinations/consultations will take place:

- Clinical examination including blood pressure and heart rate.
- Collection of the nosebleed grids filled in by the patients during the first 2 weeks of treatment (Appendix 1: Nosebleed monitoring grid), if not filled in on line. These grids include monitoring of studied treatment intake.
- The number of red blood cell transfusions received by the patient in the last 2 weeks will be noted.

- A set of biological blood samples (3 x 5 mL tubes) is collected: NFS, complete ionogram, serum ferritin and hepatic function.
- For female patients of child-bearing age, a supplementary blood tube is sampled, for βHCG dosage.
- Monitoring and collection of any adverse events
- Concomitant treatments will be noted, included iron infusions
- The investigator will connect to Ennov's platform and new treatment number will be allocated. The specific prescription sheet will be filled in for drug dispensation (150 mg capsules or 100 mg capsules according to tolerance) by the pharmacist at the hospital. A sufficient amount will be dispensed for 2 weeks of treatments (+1 day), i.e. 30 capsules (1 bottle). The patient must tick each drug intake in the table provided for this purpose on the epistaxis grids.
- Epistaxis grids will be given to the patient to record all episodes of nose bleed and their duration if not completed on line.

- **Visit at 4 and 8 weeks, V3 (D28 +/- 1 day) and V4 (D56 +/- 2 days).**

The patients will be seen in consultation, in the Reference center or skill center for HHT.

The following examinations/consultations will take place:

- Clinical examination including blood pressure and heart rate.
- Collection of the nosebleed grids filled in by the patients during the last 4 weeks of treatment (Appendix 1: Nosebleed monitoring grid), if not filled in on line. These grids include monitoring of treatment intake.
- New nosebleed grids will be given to the patients for ENT monitoring during the next 4 weeks (Appendix 1: Nosebleed monitoring grid), if not filled in online.
- The number of red blood cell transfusions received by the patient in the last 4 weeks will be noted.
- Patients will return the drug: unused capsules and empty packaging, it will be transmitted to the hospital pharmacy.
- A set of biological blood samples (3 x 5 mL tubes) is collected: NFS, complete ionogram, serum ferritin and hepatic function.
- For female patients of child-bearing age, a supplementary blood tube is sampled, for βHCG dosage.
- Monitoring and collection of any adverse events
- Concomitant treatments will be noted, included iron infusions
- The investigator will connect to Ennov's platform and new treatments numbers will be allocated. The specific prescription sheet will be filled in for drug dispensation (150 mg capsules or 100 mg capsules according to tolerance) by the pharmacist at the hospital. A sufficient amount will be dispensed for 4 weeks of treatments (+2 days), i.e. 60 capsules (2 bottles). The patient must tick each drug intake in the table provided for this purpose on the epistaxis grids.
- Epistaxis grids will be given to the patient to record all episodes of nose bleed and their duration if not completed on line.

- **Visit at the end of the treatment at 12 weeks, V5 (D84 up to 94)**

The patients will be seen in consultation, in the Reference center or skill center for HHT.

The following examinations/consultations will take place:

- Clinical examination including blood pressure and heart rate.
- Collection of the nosebleed grids filled in by the patients during the last 4 weeks of treatment (Appendix 1: Nosebleed monitoring grid), if not filled in on line. These grids include monitoring of treatment administrations.

- New nosebleed grids will be given to the patients for ENT monitoring during the next 12 weeks (Appendix 1: Nosebleed monitoring grid), if not filled in online.
- The number of red blood cell transfusions received by the patient in the last 4 weeks will be noted.
- Patients will return the drug: unused capsules and empty packaging, it will be transmitted to the hospital pharmacy.
- A set of biological blood samples (3 x 5 mL tubes) is collected: NFS, complete ionogram, serum ferritin and hepatic function.
- For female patients of child-bearing age, a supplementary blood tube is sampled, for βHCG dosage. Provide the patient with two urinary pregnancy tests in order to perform monthly check.
- Monitoring and collection of any adverse events
- Concomitant treatments will be noted, included iron infusions
- An ENT consultation will be organized with a nasal examination
- The ESS questionnaire will be filled in by patient (online or on paper sheet).
- A SF36 quality of life questionnaire will be filled in by the patient (online or on paper sheet).
- Epistaxis grids will be given to the patient to record all episodes of nose bleed and their duration if not completed on line.

7.3.4 End of study visit at 24 weeks, V6 (Day 168 from D158 to D178)

The patients will be seen in consultation, in the Reference center or skill center for HHT.

The following examinations/consultations will take place:

- Clinical examination including blood pressure and heart rate.
- Collection of the nosebleed grids filled in by the patients during the 12 weeks after the treatment (Appendix 1: Nosebleed monitoring grid), if not filled in on line.
- The number of red blood cell transfusions received by the patient in the last 12 weeks will be noted.
- A set of biological blood samples (3 x 5 mL tubes) is collected: NFS, complete ionogram, serum ferritin and hepatic function.
- For female patients of child-bearing age, a supplementary blood tube is sampled, for βHCG dosage
- Monitoring and collection of any adverse events
- Concomitant treatments will be noted, included iron infusions
- The ESS questionnaire will be filled in by patient.
- A SF36 quality of life questionnaire will be filled in by the patient.

7.4 Rules for treatment discontinuation and dose adjustment

In case of adverse reaction the treatment can be temporary interrupted until the specific reaction has resolved to levels that allow continuation of therapy (refer to § 8.3). Outside planned visits the patient will be seen by her/his usual doctor or an emergency service, who will implement a suitable treatment.
The treatment under study may be resumed as prescribed at the previous visit: at the full dose (150 mg twice daily) or a reduced dose (100 mg twice daily).
As soon as a patient has a reduced dose prescription at 200 mg per day, this dose will be maintained until the end of the treatment (without attempt to increase at the initial dose).
If a patient does not tolerate 100 mg twice daily, treatment should be discontinued (refer to §6.1).
A dose reduction cannot be implemented outside the visits provided for in the protocol.

7.5 Rules for a temporary or permanent discontinuation

- *Discontinuation of the participation of a subject in the study:*

The subjects can withdraw their consent and request to leave the study at any time and for any reason. In case of premature termination, the investigator must document the reasons as thoroughly as possible.
The investigator may temporarily or definitively interrupt a subject's participation in the study for any reason which would best serve the interests of the subject, particularly in the event of a serious adverse event.
In the event a subject is lost to follow-up, the investigator will make every effort to re-establish contact with the person.
In the event of a withdrawal of consent, the data collected up to the date of withdrawal will be analyzed.

- *Termination of part or all of the study:*

The study can be prematurely suspended in the event of unexpected serious adverse events requiring a review of the characteristics of the strategy. Likewise, unexpected events or new information concerning the method of investigation, in view of which the objectives of the study are not likely to be met, may lead the sponsor to prematurely suspend the study.
The Hospices Civils de Lyon reserves the right to suspend the study at any moment if it is determined that the inclusion objectives are not reached.
In case of the premature termination of the study for safety reasons, the sponsor will send the information to the ANSM and the EC within 15 days.

8 SAFETY ASSESSMENT

8.1 *Definitions*

According to article R1123-46 of Public Health Code

8.1.1 Adverse event

An adverse event (AE) is any untoward medical occurrence in a patient or subject which does not necessarily have a causal relationship with the investigational medicinal product at any dose.

8.1.2 Adverse reaction

An adverse reaction is any noxious and untoward medical occurrence with a reasonable causality with the investigational medicinal product at any dose.

8.1.3 Unexpected adverse reaction

Any adverse reaction which nature, severity, frequency or evolution is not consistent with safety reference information mentioned in the SmPC or in the Investigator Brochure when the product is not authorized for market.

8.1.4 Serious Adverse Events (SAE)

A **serious adverse event** (SAE) means any untoward medical event that:

- results in **death** ; or
- is **life-threatening** for any person who participates in the clinical trial; or
- requires **in patient hospitalization or prolongation of existing hospitalization**; or
- results in **persistent or significant disability / incapacity**; or
- results in a **congenital anomaly / birth defect at any dose ;** or,
- is an important medical event that does not meet the criteria listed above:
  - o an event that may be considered as "potentially serious", including certain biological abnormalities
  - o a medically relevant event according to the investigator's judgment

- an event requiring medical intervention to prevent the evolution towards one of the aforementioned condition

For instance, these events could be intensive treatment in hospital emergency rooms or at the patient's home for allergic bronchospasm, convulsive seizure or coagulation disorders. The term "life-threatening" is reserved for an immediate threat to life, at the time of the adverse event, regardless of the consequences of any corrective or palliative therapy. Certain circumstances requiring hospitalization do not fall under the criterion of severity: "hospitalization / prolongation of hospitalization". Refer to paragraph 8.2.3 for serious adverse events that do not require prompt notification to the sponsor.

8.1.5 New issue

Any new data which can lead to a re-assessment of benefit-risk ratio of the research or the investigational drug, or to a modification of product use, on the research management, or to change of research documents, or to the suspension or interruption of the research or similar research.

8.2 ***<u>Investigator`s responsibilities</u>***

8.2.1 Procedures for detection and reporting of the adverse events

All adverse events have to be investigated, reported and recorded, treated and evaluated from the first visit (inclusion D0) until the end of study and their resolution. All adverse events must be recorded in the Adverse Event Reporting Forms of the Case Report Form (CRF). Each observed adverse event will be recorded individually. The intensity of the event will be graded according to the following classification:

- **Mild (grade 1):** No disruption of normal daily activity
- **Moderate (grade 2):** Discomfort sufficient to reduce or affect normal daily activity
- **Severe (grade 3):** Incapacity and inability to work or perform normal daily activity
- **Life-threatening (grade 4)**
- **Death (grade 5)**

All adverse events should be graded. **All adverse events of severe intensity, life-threatening grade, and death (grade 3 or above) shall be considered as SERIOUS** and must be notified to the sponsor without delay *unless they are described in paragraph 8.2.3 as not to be notified without delay to the sponsor.*

Specificity of adverse event collection in this study:

Considering the studied pathology, **epistaxis and related anemia** will not be recorded as an adverse event, nevertheless it will be collected in the CRF (epistaxis grids and biological analysis).

In case of bleeding the localization have to be specified.

8.2.2 Serious adverse event reporting

The investigator evaluates each adverse event in terms of severity.
The investigator shall notify to the sponsor <u>all serious adverse events</u> occurring during the trial, without delay and no later than 24 hours from the day on which the investigator becomes aware of it, with the exception of those identified in the protocol as not requiring notification without delay.
This initial notification shall be the subject of a written report and shall be followed by one or more additional detailed written report(s) within the **8 days** following the first notification.
The investigator validates dates and sends the SAE form, by email via eCRF to drci.eig-vigilance@chu-lyon.fr, with at least these 4 criteria which are mandatory to submit the SAE:

- An investigator
- A subject
- An experimental product (if applicable)
- An adverse event

The investigator must document the event as well as possible (by means of copies of laboratory results or reports of examinations or hospitalizations, including relevant negative results, **ensuring documents are anonymized** and entering the patient's number and code), **medical diagnosis** and establish a **causal relationship** between the serious adverse event and the drug(s).

The patient who has experienced an SAE must be followed until complete resolution, stabilization at an acceptable threshold according to the investigator or recovery to his previous state, even if the patient has been withdrawn from the trial. The investigator has to inform the sponsor by completing the eCRF, then email to via eCRF to drci.eig-vigilance@chu-lyon.fr (fulfill the part corresponding to follow-up (FU) with the name, the date and the signature in eCRF)

If the eCRF is unavailable, the investigator can scan the SAE form signed and dated and send it by email to drci.eig-vigilance@chu-lyon.fr or by fax on 04 72 11 51 90. It must specify in the subject line of the email "STUDY NAME-Severity criterion * -N° patient center".
* to be filled according to the severity criterion selected in the EIG notification form

8.2.3 Serious adverse events that do not require prompt notification to the sponsor (no SAE form to send but SAE to collect only in the CRF)

The following events do not require immediate notifications of SAE, indeed they are not relevant to the research and they do not imply a safety concern for the patient.

- Admission for social or administrative reasons;
- Hospitalization scheduled in the protocol;
- Hospitalization for medical or surgical treatment scheduled before the research;
- Transition to a day hospital scheduled for the follow-up of the studied condition or for an intercurrent disease already known at inclusion;
- Admission to emergencies lasting less than 24 hours (not related to the treatment)
- Nose bleeding and related transfusions without life threatening.
- Anemia, grade 1 to 3 (CTCAE).

Nevertheless these adverse events are to be collected in the Case Report Form (CRF).

8.2.4 Adverse Events with specific interest (AESI)

Some events require special monitoring and will be notified as a SAE:

- Any adverse event of any grade which required a treatment interruption exceeding 7 consecutive days.
- Gastrointestinal perforation
- Hepatic Injury

Definition of Hepatic injury:
Signs of hepatic injury are defined as:

- *ALT and/or AST ≥8 fold ULN*
- *ALT and/or AST ≥3 fold ULN and total bilirubin ≥ 2 fold ULN**
- *ALT and/or AST ≥3 fold ULN and unexplained INR > 1.5**
- *ALT and/or AST ≥3 fold ULN and unexplained eosinophilia (>5%)**
- *ALT and/or AST ≥3 fold ULN and appearance of fatigue, nausea, vomiting, right upper abdominal quadrant pain or tenderness, fever and/or rash.*

** in the same blood draw sample.*

8.2.5 In utero exposition

If a woman becomes pregnant during the research or if the partner of a male patient becomes pregnant, pregnancy must be reported to the sponsor.
The investigator informs the sponsor (by phone, fax or email) who will send to the investigator a pregnancy form. This form must include the expected date of delivery if the pregnancy is still ongoing.
The pregnancy should be followed up by the investigator until delivery or its interruption, who also has to notify the outcome to the sponsor.
If the outcome of pregnancy meets the criteria of a serious adverse event (spontaneous abortion with hospitalization, fetal death, congenital anomaly...) the investigator must follow the procedure for SAE reporting.

8.2.6 Causality assessment

The investigator must assess the causality of adverse events with the experimental drug(s) and with the procedures / acts added by the research. He must also assess the causality of adverse events with the other concomitant treatments taken by the patient and provide the results of this evaluation to the sponsor: related (certain, probable, possible, unlikely) or not related.

8.2.7 Reporting time frames of SAE without delay to the sponsor by the investigator and procedures for monitoring serious adverse events

The investigator must notify to the sponsor without delay all serious adverse events:

- from the INCLUSION OF THE PATIENT (date of signature of the 1st consent)
- until THE END OF PARTICIPATION OF THE PATIENT - With no time limit for serious adverse events related to the research (for instance: cancers, congenital malformations occurring in the long term after exposure to the experimental drug...).

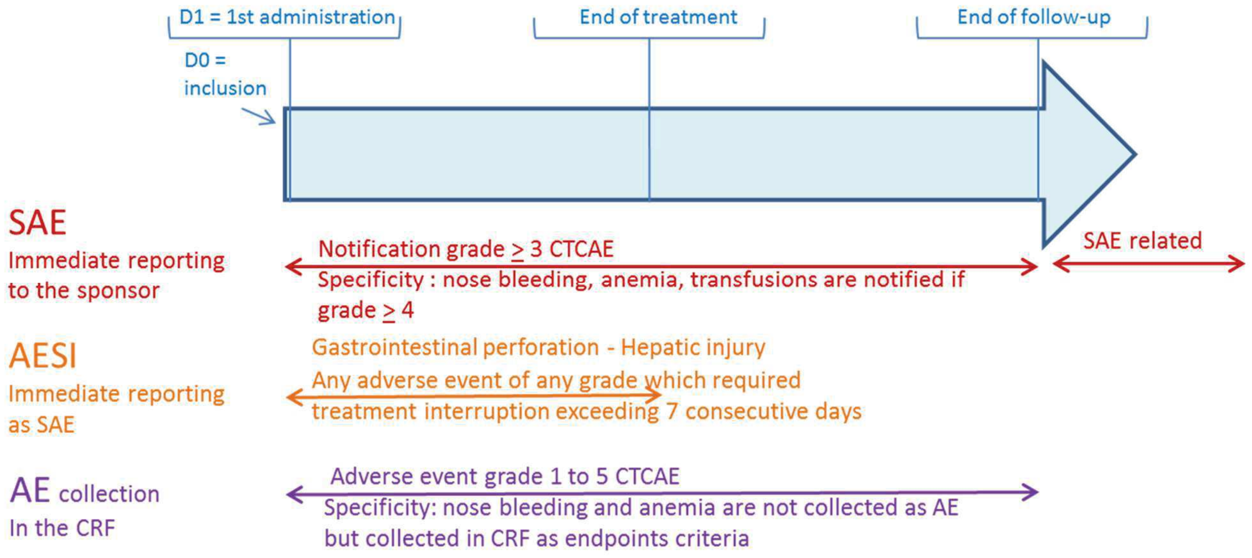

Figure 15: Adverse Event Flow Chart

8.3 *Specific management of complications*

8.3.1 Gastrointestinal disorders

Diarrhea

Diarrhea should be treated at first signs with adequate hydration and anti-diarrheal medicinal products, e.g. loperamide, and may require treatment interruption or dose reduction (100 mg twice daily, according to the following algorithm (Figure 15)

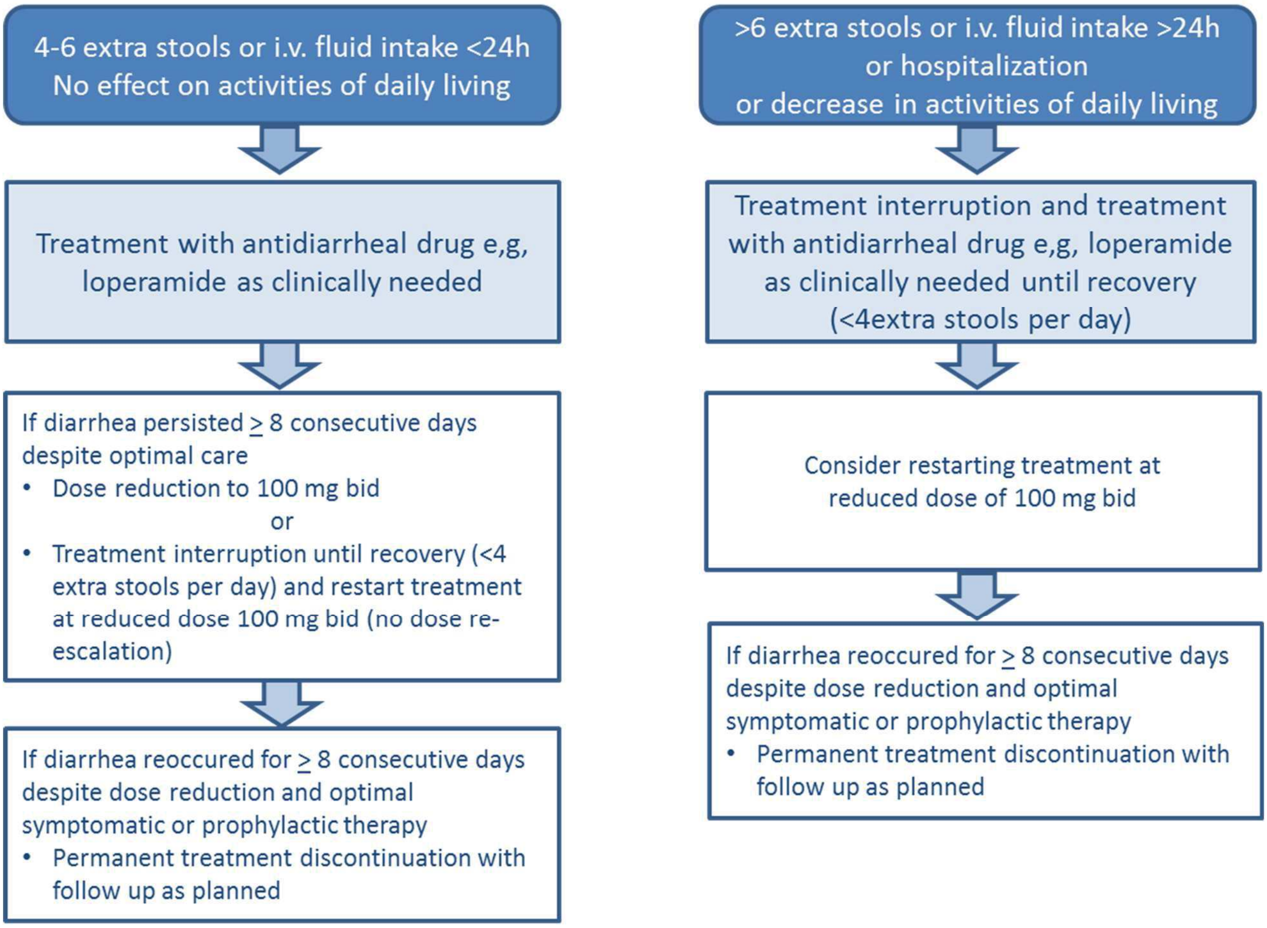

Figure 16: Recommendations for the management of diarrhea adverse events in the study

Nausea and vomiting

Nausea and vomiting were frequently reported adverse events. In most patients with nausea and vomiting, the event was of mild to moderate intensity. Nausea led to discontinuation of nintedanib in 2.0% of patients. Vomiting led to discontinuation in 0.8% of the patients.

If symptoms persist despite appropriate supportive care (including anti-emetic therapy), dose reduction or treatment interruption may be required. The treatment may be resumed at a reduced dose (100 mg twice daily). In case of persisting severe symptoms therapy with nintedanib should be discontinued.

Gastrointestinal perforations

The treatment should be permanently discontinued in patients who develop gastrointestinal perforation.

8.3.2 Hepatic function

If transaminase (AST or ALT) elevations > 3x upper limit normal (ULN) are measured, dose reduction (100 mg daily) or interruption of the therapy with nintedanib is recommended and the patient should be monitored closely according to the following algorithm (figure 16).

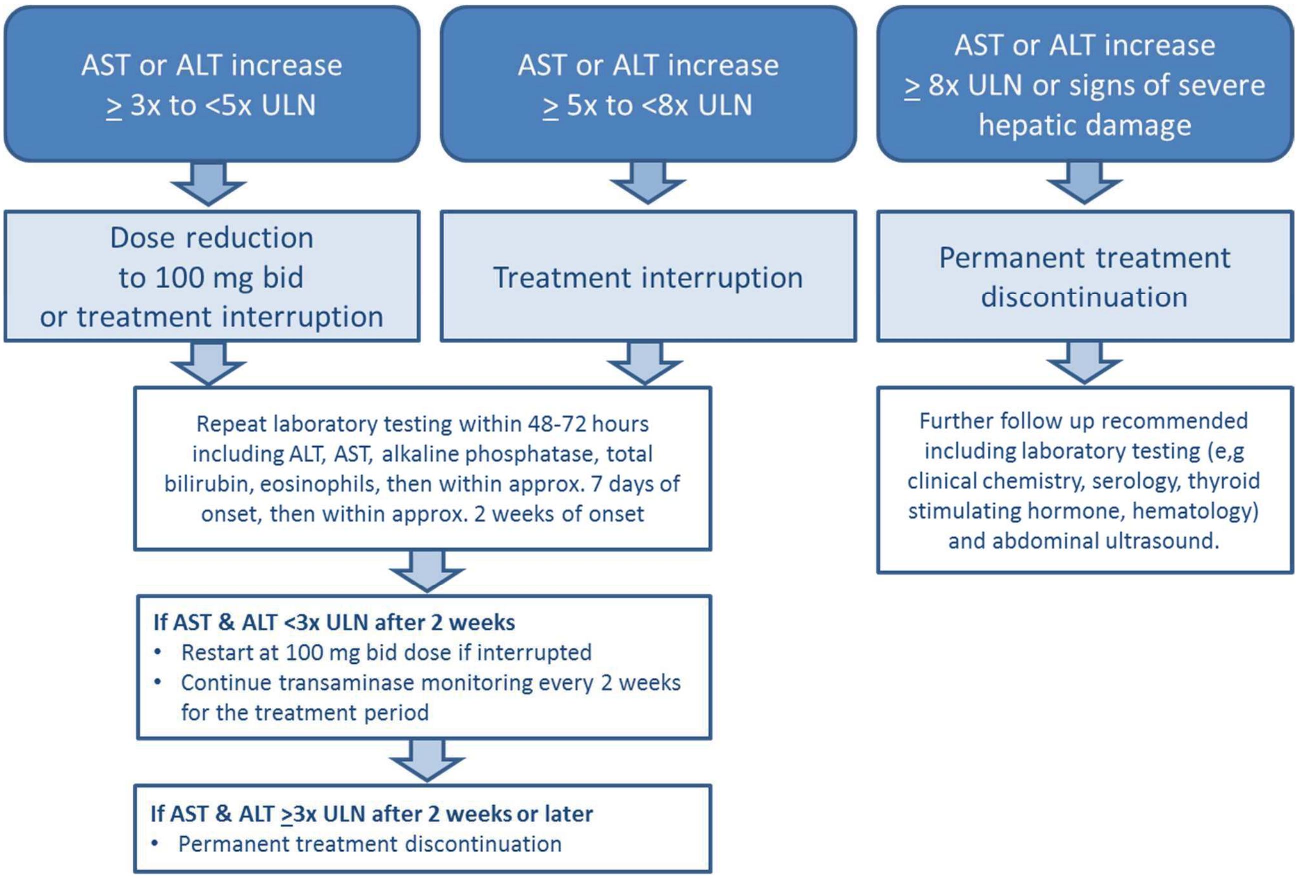

Figure 17: Recommendations for the management of hepatic enzyme elevations in the study

8.3.3 Arterial thromboembolic events

Treatment interruption should be considered in patients who develop signs or symptoms of acute myocardial ischemia.

8.3.4 Surgery

Based on the mechanism of action nintedanib may impair wound healing. Patient who have surgery have to stop experimental treatment.

8.3.5 Hemorrhages

8.3.5.1 Nosebleeds

Patients will have access to the standard management techniques for nosebleeds in HHT. ENT monitoring will be ensured before the start of administration and after 12 weeks of treatment to note any modification to the nasal mucosa during the treatment.
The modalities for the management of nosebleeds will be given to the patients, as described in the "PNDS" (Protocole National de Diagnostic et de Soins), so that they may inform any doctor of the treatment modalities for the nosebleeds specific to this pathology. Emergency document accessible on the internet http://www.favamulti.fr/wp-content/uploads/2016/05/37_RO-Epistaxis.pdf, is given to the patients.

8.3.5.2 Hemoptysis

The treatment will be stopped and an adapted medical care will be set up.

8.3.5.3 Gastrointestinal bleeding

The treatment will be stopped and a digestive endoscopy will be performed.

8.4 Responsibility of the sponsor

8.4.1 Declaration to the competent authorities

According to article R1123-54 of the Public Health Code, the sponsor shall report:
- to ANSM and Eudravigilance, any suspected unexpected serious adverse reaction (SUSAR) occurring in France and outside the national territory within the following time frames:
    - in case of life threatening or death : without delay from the day on which the sponsor becomes aware of it, and the relevant additional information to be submitted as a follow-up report to ANSM within the 8 days following the initial report.
    - for all other unexpected serious adverse reactions: no later than 15 days from the day on which the sponsor becomes aware of it, and the relevant additional information to be submitted as a follow-up report to ANSM within another 8 days following the initial report.
- to ANSM and to CPP, any new safety issue and, when appropriate, the measures taken without delay from the day on which the sponsor is aware of them and the relevant additional information to be submitted in the report form to ANSM within the 8 days following the initial report.

The sponsor will also prepare a Development Safety Update Report (DSUR) which will be forwarded to ANSM and CPP within the 60 days following the birth date of the study (authorization's date of ANSM).

8.4.2 Safety reference information for the assessment of the expectedness / unexpectedness

The expectedness or unexpectedness of a suspected serious adverse reaction is assessed from:
- Investigational Drug Reference Document n°1: Summary of Product Characteristics (SmPC) of OFEV® (Nintedanib)
- Investigators' brochure, doc. N° c01783972-17 current version. Any updates, transmitted by BI, will be communicated to all investigators.

8.5 *Data Safety Monitoring Board (DSMB)*

The Data Safety Monitoring Board (DSMB) is an advisory committee responsible to help the sponsor to proactively monitor and gauge patient safety and risk in the clinical trial. Therefore, the DSMB reviews the data and issues that may occur during the trial, especially the ones which are scientific, ethical and tolerance, which may change the benefit / risk ratio. Following this review, the DSMB shall provide its recommendations by writing to the sponsor. These recommendations may concern in particular the continuation, modification or termination of the study.
The sponsor remains responsible for the decision of the measures to be implemented, based on the recommendations of the DSMB.
The modalities of organization of this DSMB are described in a charter signed by the members of the DSMB at the beginning of the research. The DSMB includes one clinical experts, a methodologist/biostastistician and a pharmacist.

9 STATISTICAL CONSIDERATIONS

9.1 Number of subjects required

We hypothesize that 60% of patients will be improved in the treatment group against 15% in the placebo group. It is therefore necessary to include 27 patients in each group to reach a 90% power, leading to 54 patients overall.

Taking into account early withdrawal and patients who may be lost to follow-up, we are going to include 30 patients in each group, that is to say, a total of 60 patients.

9.2 Description of the statistical methods

9.2.1 Study populations

The intention-to-treat population is defined as all included patients having started the treatment. The per protocol population is defined as all included patient who have received at least 80% of the total treatment, that is to say at least 134 capsules (from day 1 to day 84) and without permanent discontinuation prescribed by the investigator. In case of wrong treatment allocation: the patient will be excluded from PP population if he was in placebo group and he received nintedanib treatment; if he was in nintedanib group and received placebo, he could be included in the PP population as long as he received at least 80% of the total treatment. A flow chart will present the population.

9.2.2 Protocol deviations

Any protocol deviation that could affect the results will be listed.

9.2.3 Baseline characteristics

All baseline characteristics will be summarized using descriptive statistics (number, mean, standard deviations, median, minimum and maximum for quantitative variables; and number and percentages for qualitative variables).

9.2.4 General considerations

As the total sample size is expected to be low, all proposed tests will be non-parametric. A pvalue <0.05 will be considered as significant.

9.2.5 Main judgment criterion analysis (intention-to-treat population)

The proportion of patients experiencing improvement in their nosebleeds (reduction of at least 50% on epistaxis monthly mean duration) will be computed in each group.
The pre-treatment period will cover all recorded epistaxis during 8 weeks (56 days) before inclusion. The treatment period will start after 4 weeks of treatment and will last 56 days (8 weeks). A mean over 4 weeks (28 days) will be computed. For each period, the total duration will be computed and will be normalized (total duration X (28/56 days)) to obtain a monthly (4 weeks) mean.

The proportion of the two groups will be compared using a Fisher exact test.

The analysis will then be performed on the per protocol population to check for robustness.

9.2.6 Secondary judgment criterion analysis

1. **Safety**

– All adverse events and severe adverse events observed during the study will be collected and coded with MedDRA. The International Common Terminology Criteria for Adverse Events (CTCAE) current version will be used for adverse event reporting.

– Nintedanib safety in HHT patients will be assessed in comparing occurrence of adverse events in both arms (Nintedanib vs placebo).

2. **Efficacy on epistaxis :**

- Proportion of participants reporting a response at the end of follow-up (at least 50% reduction on epistaxis monthly mean duration) will be compared between groups with the Fisher exact test. The monthly mean duration over the last 8 weeks of the follow-up will be computed and will be compared between groups using Mann-Whitney test.
- Absolute and relative change of epistaxis monthly mean duration from baseline will be computed for the last 8 weeks of the treatment period and for the last 8 weeks of the follow-up period and compared between both groups using Mann-Whitney test.
- Mean duration of epistaxis will be computed all over the study period using a 4-week gathering, the results will be presented graphically to show the evolution according to time and treatment.
- Nosebleeds frequencies over the last 8 weeks of the treatment period and over the last 8 weeks of the follow-up will be compared between groups as well as the corresponding absolute and relative changes from baseline. These numbers will be compared between groups using Mann-Whitney test.
- ESS scores at the end of the treatment period and at the end of the follow-up will be compared between groups as well as the corresponding absolute changes from baseline (Mann-Whitney test).

3. **Efficacy on other clinical criteria :**

- Values of SF36 score and relative change from baseline at the end of the treatment and end of the follow up will be compared between groups using a Mann-Whitney test. Values for sub-scales will be presented.
- Number of red blood cell transfusions (discrete variable): Number of red blood cells transfusions during the treatment period and during the follow-up period will be compared between groups as well as the corresponding absolute change from baseline. They will be compared between groups with a Mann-Whitney test
- Number of iron infusions (discrete variable): Number of iron infusion during the treatment period and during the follow-up period will be compared between groups with Chi-squared test and Mann-Whitney test as well as the corresponding absolute change from baseline (Mann-Whitney test).

4. **Efficacy on biological criteria:**

- Hemoglobin level (continuous): Hemoglobin levels at the end of the treatment period and at the end of the follow-up will be compared between groups as well as the corresponding relative changes from baseline.
- Ferritin level (continuous): Ferritin levels at the end of the treatment period and at the end of the follow-up will be compared between groups as well as the corresponding relative changes from baseline.

9.3 Method of accounting for missing data

Follow-up of patients will be pursued whatever the situation excepted if the patient withdraws his consent.

In the case of nasal surgery during the study period or any other treatment substituting to the studied treatment, the patient will be followed but his result will be considered as a failure.

Regarding the main judgment criterion: epistaxis criterion is based on grids filled in daily by patients. They are used to these grids and fill them in carefully. However in case of missing data the following procedure will be applied:

- If less than 14 days (included) are missing over the whole period evaluated: the monthly mean duration will be computed from the data available (from the 8 weeks, 56 days, period evaluated),
  Monthly mean duration = total duration recorded X (28/number of day available for the reporting period).
- If a patient is lost to follow-up or refused to communicate nosebleeds grids or has more than 14 days missing on grids, the result for the concerned patient will be considered as a failure.

9.4 Management of modifications brought to the analysis plan

A detailed statistical analysis plan will be written before the database is frozen. It will take into account all protocol modifications or all unexpected events occurring throughout the study and having an impact on the analyses presented here. The planned analyses may be completed in line with the study objectives.
All modifications subsequently brought to the statistical analysis must be justified and will result in a new version of the document. These deviations in the analysis plan will be reported in the final study report. All the documents will be stored in the study folder.

9.5 Person responsible for the analyses and the software used

Dr Evelyne Decullier will be the person responsible for the analysis. Clinsight (Ennov clinical) software will be used to manage this study.

10 MONITORING THE STUDY

10.1 Scientific committee

The Scientific Committee for the study, presided over by the study's main investigator, will be composed of investigators (one ENT specialist and one geneticist), the methodologist and the sponsor of the study. The committee will be responsible for validating the definitive version of the protocol, supervising the implementation and running of the study, and for writing the reports and publications resulting from the study.

10.2 Independent monitoring committee

Monitoring the safety of administration of the product, motivated by the iatrogenic risks, justifies the setting up of a specific independent monitoring and safety committee.

A charter will define the primary responsibilities of DSMB, its relationship with other trial components, its membership, and the purpose and timing of its meetings. This charter will be accepted and signed by each member.

It will be composed of specialists not involved in the study:

- a specialist of the disease,
- a pharmacist,
- a statistician specializing in the methodology of clinical trials.

This committee will meet at least once a year and in case of occurrence of serious adverse events evoking a toxicity of the treatment under study.

This committee is responsible for identifying mechanism for the completion of various tasks that will impact the safety and efficacy of all study procedures and overall conduct of EPICURE study.

DSMB recommendations will be forwarded to ANSM for information.

10.3 Adverse events assessment committee

This committee will be composed of 10 investigators, HHT specialists, in each of the 10 centers in charge of patients in the study, the methodologist. A representative of safety department from the sponsor will be invited.

They will meet regularly, about twice a year according to the study advancement, by conference call.

All adverse events collected during the period in the eCRF, and monitored, will be discussed. The relationship between the adverse event and nintedanib will be evaluated and graduated according to chronological and semiological criteria (0: not related; 1: doubtful; 2: possible; 3: likely; 4: very likely).

11 RIGHT OF ACCESS TO THE DATA AND SOURCE DOCUMENTS

11.1 Access to the data

In conformity with the GCP:

- the sponsor is responsible for obtaining the agreement of all the parties implicated in the study in order to guarantee direct access to all the sites where the study will take place, to the source data, source documents, and reports, in the interests of quality control and audits by the sponsor;

- the investigators will provide the persons responsible for the follow-up, the quality control, or the audit of the study involving human individuals, the individual documents and data that are strictly necessary for this control, in accordance with the current legal and regulatory provisions (article L1121-3 and R.5121-13 of the public health code).

11.2 Source documents

Source documents are defined as all documents or original objects allowing the existence or accuracy of data, or a fact recorded during the clinical study, to be proven. They will be kept for 25 years by the investigator or by the hospital if it is a patient's hospital file.

In this study, source documents are: medical file (paper & electronic) including biological examination results, questionnaires (ESS & SF36) filled in by patient, epistaxis grids, prescription sheets and ENT examination sheet.

11.3 Data confidentiality

In accordance with provisions concerning the confidentiality of data to which persons responsible for the quality control of a study involving human individuals have access (article L.1121-3 of the public health code), and in accordance with the provisions regarding the confidentiality of information relating, in particular, to the trial, the persons who participate, and the results obtained (article R.5121-13 of the public health code), the persons having direct access to the data will take all necessary precautions to ensure the confidentiality of the information related to the trials, to the persons participating and, in particular, with regards to their identity as well as the results obtained.

These persons, such as the investigators themselves, are subject to professional confidentiality (in accordance with the conditions defined by articles 226-13 and 226-14 of the penal code).
During the research involving human individuals or at its end, the data collected on the persons participating and sent to the sponsor by the investigators (or any other specialists) will be made anonymous.

Under no circumstances should the names or the addresses of persons concerned appear.

Only the first letter of the subjects surname and the first letter of their first name shall be recorded, accompanied by a coded number specific to the study indicating the inclusion order of the subject.

The sponsor will ensure that each person participating in the research has given their written agreement granting access to the individual data that concerns them and strictly necessary for the quality control of the study.

12 QUALITY CONTROL AND ASSURANCE

A Clinical Research Associate (CRA) mandated by the sponsor will ensure the proper conduct of the study, collection of written data, their documentation, recording and reporting, in accordance with the Standard Operating Procedures put in place within the DRCI of the Hospices Civils de Lyon and in conformity with the Good Clinical Practices as well as the current legal and regulatory provisions.

The investigator and the members of their team will accept to make themselves available during the Quality Control visits performed at regular intervals by the Clinical Research Associate. During these visits, the following elements may be reviewed depending on the level of monitoring appropriate for the study and determined in accordance with the SOPs of the sponsor:
Cat. 1 study, according to the monitoring plan

- Informed consent
- respect of the study protocol and the procedures defined therein
- quality of the data recorded in the case report form: accuracy, missing data, consistency of the data with the source documents
- Management of the experimental treatments
- declaration of serious adverse events

All visits will be the subject of a written monitoring report and a follow-up letter addressed to the investigator of the site visited and to the study coordinating structure.

Furthermore, the investigators agree to accept the quality control audits carried out by persons mandated by the sponsor as well as inspections by the competent authorities. All data and all documents and reports may be the subject of regulatory audits and inspections without the possibility of using medical secrecy as opposition.

13 Ethical considerations

13.1 Competent authorities

The protocol, the written information sheet and the consent form for the study will be submitted to ethics committee *XX for an opinion (specify the ethics committee number after notification).*
The notification of the favorable opinion from the EC will be sent to the study sponsor and the ANSM. The sponsor will also send a study authorization request to the ANSM.
The sponsor undertakes to ensure that the start of the study only occurs after the favorable opinion of the EC and the study authorization from the ANSM have been obtained.

13.2 Substantial modifications

In the event that a substantial modification is made to the protocol by the investigator, it will be approved by the sponsor. Before its implementation, the latter must obtain a favorable opinion from the EC and an authorization from the ANSM within the scope of their respective competencies. A new consent will be collected from the people already participating in the study, if necessary.

13.3 Patient information and written consent form

Patients will be fully and faithfully informed, in a comprehensive manner, of the objectives and restrictions of the study, of the potential risks, of the monitoring and security measurements required, of their right to decline participation in the study, or of the possibility to withdraw at any moment.
All this information will be presented in the information sheet and informed consent form given to the patient.
The patient's free and informed written consent will be collected by the investigator or a doctor representing them before the definitive inclusion into the study. A copy of the information notice and the consent form signed by the two parties will be given to the patient, the investigator will keep the original.

13.4 Declaration of conformity

The sponsor and the investigator undertake to ensure that the study is conducted:
- in conformity with the protocol,
- in conformity with both the French and international good clinical practices currently in force,
- in conformity with the current French and international legal and regulatory provisions.

13.5 Exclusion period

A simultaneous participation in another study can be possible if it does not interfere with the ongoing trial, the patient should inform investigator about its participation.

At the end of the study there will be no exclusion period for the patient.

13.6 Compensation for the subjects and registration in the national register of people participating in a cat. 1 interventional study on human individuals

No compensation is planned for the volunteers who accept to participate in the study. When the objective of the study is related to the pathology of the subjects, registration in the national register of Volunteers participating in a Biomedical Research (BRV) is not required.

14 MANAGEMENT AND STORAGE OF DATA

14.1 Electronic Case report form

The case report form will only include the data necessary for an analysis for a scientific publication. Other patient data necessary for their follow-up outside of this study will be collated in their medical file.

All information required by the protocol should be recorded in the case report form. Data must be collected as it is obtained and explicitly recorded in these case report forms. All missing data must be encoded.
This electronic case report form will be put in place in each center through an internet portal for recording the data. A help document for using this tool will be provided to the investigators.

The investigator is responsible for the accuracy, quality, and pertinence of all the data entered. Furthermore, during entry, these data are immediately verified thanks to coherence checks. As such, the investigator must validate any value changes in the CRF. These changes are part of an audit trail. A reason is integrated as a comment.

14.2 Electronic Data management

The Clinical Research Unit at the Pôle Santé Publique will develop an electronic Case Report Form (Ennov Clinical –Clinsight) to collect data for the study.
The study data will be computerized in conformity with the law concerning electronic data, files and civil liberties (law 78-17 of 6 January 1978 modified by law 2004-801 of 6 August 2004). Data access will be restricted.

Electronic report forms will be available for each participating center. Electronic case report forms will be filled via internet by the investigator. A user guide and an online training will be available to investigators.
The investigator is responsible for the accuracy, quality and relevance of all captured data. As such, it must validate any changes in value in the eCRF. Data changes will be drawn in an audit trail and justified.
Patients' eCRF will be printed at the end of study and will be archived by the investigator.

When entering data, system will be immediately run consistency checks.
Coherence controls will also be carried out using the SAS software.

14.3 CNIL

This study falls within the framework of the "Reference Methodology" (RM-001) under the provisions of article 54, paragraph 5 of modified law no. 78-17 from January 6 1978, related to information technology, files and liberties. This alteration has been approved by the decision from January 5 2006 and modified on July 21 2016. The Hospices Civils de Lyon, sponsor of the study, have signed a commitment of compliance to this "Reference Methodology".

14.4 Archiving

The following documents will be archived under the name of the study and under the responsibility of the coordinating investigator or associated investigators in each site for 25 years.

- Protocol and annexes, possible amendments,

- Original signed copies of the information and consent forms
- Individual data (certified copies of raw data)
- Follow-up documents and letters relating to the study

The sponsor is also responsible for organizing the storage of the statistical analyses and the final study report for the required duration of archiving.
No moving or destruction can be carried out without the agreement of the sponsor. At the end of the 25 years, the sponsor will be consulted for the destruction. All data, documents, and reports may be the subject of an audit or inspection.

15 FUNDING AND INSURANCE

15.1 Study budget

The **total budget** is **512 734 €** for 60 patients included.

Drugs will be provided by the company Boehringer Ingelheim International GmbH.

15.2 Insurance

The sponsor has subscribed to an insurance policy for the entire duration of the study, covering its own civil liability as well as that of all the doctors involved in the realization of the study. It will also insure the full compensation for harmful consequences of the research for the participating persons and their beneficiaries, except with evidence, at their responsibility, that the damage is not attributable to their mistake or to that of all consultants, without the possibility of being opposed to an act by a third party or the voluntary withdrawal of the person who had initially consented to participate in the research.
The insurance contract was signed before the start of the study with the Société Hospitalière d'Assurance Mutuelle, 18 rue Edouard Rochet, 69008 Lyon, under the number 159.077.

16 RULES RELATING TO THE PUBLICATION

Scientific communications and reports related to this study will be carried out under the responsibility of the study's principal investigator with the agreement of the associated investigators. The co-authors of the report and the publications will be the investigators and doctors involved, in proportion to their contribution to the study, as well as the biostatistician, the methodologist and the associated researchers.
The publications rules will follow international recommendations (N Engl J Med, 1997; 336:309-315).
The study will be registered on the freely accessible clinical trials register (clinicaltrials.gov) before the inclusion of the 1st patient.

17 REFERENCES

1. Shovlin CL, Guttmacher AE, Buscarini E, et al. Diagnostic criteria for hereditary hemorrhagic telangiectasia (Rendu-Osler-Weber syndrome). *Am J Med Genet.* Mar 6 2000;91(1):66-67.
2. Haitjema T, ten Berg JM, Overtoom TT, Ernst JM, Westermann CJ. Unusual complications after embolization of a pulmonary arteriovenous malformation. *Chest.* May 1996;109(5):1401-1404.
3. Kjeldsen AD, Oxhoj H, Andersen PE, Green A, Vase P. Prevalence of pulmonary arteriovenous malformations (PAVMs) and occurrence of neurological symptoms in patients with hereditary haemorrhagic telangiectasia (HHT). *J Intern Med.* Sep 2000;248(3):255-262.
4. Shovlin CL, Letarte M. Hereditary haemorrhagic telangiectasia and pulmonary arteriovenous malformations: issues in clinical management and review of pathogenic mechanisms. *Thorax.* Aug 1999;54(8):714-729.
5. Cottin V, Plauchu H, Bayle JY, Barthelet M, Revel D, Cordier JF. Pulmonary arteriovenous malformations in patients with hereditary hemorrhagic telangiectasia. *Am J Respir Crit Care Med.* May 1 2004;169(9):994-1000.
6. Krings T, Chng SM, Ozanne A, Alvarez H, Rodesch G, Lasjaunias PL. Hereditary hemorrhagic telangiectasia in children: endovascular treatment of neurovascular malformations: results in 31 patients. *Neuroradiology.* Dec 2005;47(12):946-954.
7. Mandzia JL, terBrugge KG, Faughnan ME, Hyland RH. Spinal cord arteriovenous malformations in two patients with hereditary hemorrhagic telangiectasia. *Childs Nerv Syst.* Mar 1999;15(2-3):80-83.
8. Matsubara S, Bourdeau A, terBrugge KG, Wallace C, Letarte M. Analysis of endoglin expression in normal brain tissue and in cerebral arteriovenous malformations. *Stroke.* Nov 2000;31(11):2653-2660.
9. Willemse RB, Mager JJ, Westermann CJ, Overtoom TT, Mauser H, Wolbers JG. Bleeding risk of cerebrovascular malformations in hereditary hemorrhagic telangiectasia. *J Neurosurg.* May 2000;92(5):779-784.
10. Easey AJ, Wallace GM, Hughes JM, Jackson JE, Taylor WJ, Shovlin CL. Should asymptomatic patients with hereditary haemorrhagic telangiectasia (HHT) be screened for cerebral vascular malformations? Data from 22,061 years of HHT patient life. *J Neurol Neurosurg Psychiatry.* Jun 2003;74(6):743-748.
11. Begbie ME, Wallace GM, Shovlin CL. Hereditary haemorrhagic telangiectasia (Osler-Weber-Rendu syndrome): a view from the 21st century. *Postgrad Med J.* Jan 2003;79(927):18-24.
12. Buscarini E, Danesino C, Olivieri C, et al. Doppler ultrasonographic grading of hepatic vascular malformations in hereditary hemorrhagic telangiectasia -- results of extensive screening. *Ultraschall Med.* Sep 2004;25(5):348-355.
13. Buscarini E, Plauchu H, Garcia Tsao G, et al. Liver involvement in hereditary hemorrhagic telangiectasia: consensus recommendations. *Liver Int.* Nov 2006;26(9):1040-1046.
14. Bauer T, Britton P, Lomas D, Wight DG, Friend PJ, Alexander GJ. Liver transplantation for hepatic arteriovenous malformation in hereditary haemorrhagic telangiectasia. *J Hepatol.* May 1995;22(5):586-590.
15. Azoulay D, Precetti S, Emile JF, et al. [Liver transplantation for intrahepatic Rendu-Osler-Weber's disease: the Paul Brousse hospital experience]. *Gastroenterol Clin Biol.* Oct 2002;26(10):828-834.
16. Lerut J, Orlando G, Adam R, et al. Liver transplantation for hereditary hemorrhagic telangiectasia: Report of the European liver transplant registry. *Ann Surg.* Dec 2006;244(6):854-862; discussion 862-854.
17. Dupuis-Girod S, Chesnais AL, Ginon I, et al. Long-term outcome of patients with hereditary hemorrhagic telangiectasia and severe hepatic involvement after orthotopic liver transplantation: a single-center study. *Liver Transpl.* Mar 2010;16(3):340-347.
18. Lesca G, Burnichon N, Raux G, et al. Distribution of ENG and ACVRL1 (ALK1) mutations in French HHT patients. *Hum Mutat.* Jun 2006;27(6):598.

19. Babin E, Borsik M, Braccard S, et al. [Treatments of hereditary hemorrhagic telangiectasia of the nasal mucosa]. *Rev Laryngol Otol Rhinol (Bord).* 2005;126(1):43-48.

20. Hoag JB, Terry P, Mitchell S, Reh D, Merlo CA. An epistaxis severity score for hereditary hemorrhagic telangiectasia. *Laryngoscope.* Apr 2010;120(4):838-843.

21. Yin LX, Reh DD, Hoag JB, et al. The minimal important difference of the epistaxis severity score in hereditary hemorrhagic telangiectasia. *Laryngoscope.* May 2016;126(5):1029-1032.

22. Folz BJ, Tennie J, Lippert BM, Werner JA. Natural history and control of epistaxis in a group of German patients with Rendu-Osler-Weber disease. *Rhinology.* Mar 2005;43(1):40-46.

23. Lennox PA, Harries M, Lund VJ, Howard DJ. A retrospective study of the role of the argon laser in the management of epistaxis secondary to hereditary haemorrhagic telangiectasia. *J Laryngol Otol.* Jan 1997;111(1):34-37.

24. Klepfish A, Berrebi A, Schattner A. Intranasal tranexamic acid treatment for severe epistaxis in hereditary hemorrhagic telangiectasia. *Arch Intern Med.* Mar 12 2001;161(5):767.

25. Sabba C, Gallitelli M, Palasciano G. Efficacy of unusually high doses of tranexamic acid for the treatment of epistaxis in hereditary hemorrhagic telangiectasia. *N Engl J Med.* Sep 20 2001;345(12):926.

26. Lozano M. Tranexamic acid in hereditary hemorrhagic telangiectasia. *N Engl J Med.* Feb 7 2002;346(6):457.

27. Gaillard S, Dupuis-Girod S, Boutitie F, et al. Tranexamic acid for epistaxis in hereditary hemorrhagic telangiectasia patients: a European cross-over controlled trial in a rare disease. *J Thromb Haemost.* Jul 10 2014.

28. David L, Feige JJ, Bailly S. Emerging role of bone morphogenetic proteins in angiogenesis. *Cytokine Growth Factor Rev.* Jun 2009;20(3):203-212.

29. David L, Mallet C, Keramidas M, et al. Bone morphogenetic protein-9 is a circulating vascular quiescence factor. *Circ Res.* Apr 25 2008;102(8):914-922.

30. Bailly S, Dupuis-Girod S, Plauchu H. [Rendu-Osler disease: clinical and molecular update]. *Med Sci (Paris).* Oct 2010;26(10):855-860.

31. Oh SP, Seki T, Goss KA, et al. Activin receptor-like kinase 1 modulates transforming growth factor-beta 1 signaling in the regulation of angiogenesis. *Proc Natl Acad Sci U S A.* Mar 14 2000;97(6):2626-2631.

32. Tual-Chalot S, Mahmoud M, Allinson KR, et al. Endothelial depletion of Acvrl1 in mice leads to arteriovenous malformations associated with reduced endoglin expression. *PloS one.* 2014;9(6):e98646.

33. Park SO, Wankhede M, Lee YJ, et al. Real-time imaging of de novo arteriovenous malformation in a mouse model of hereditary hemorrhagic telangiectasia. *J Clin Invest.* Nov 2009;119(11):3487-3496.

34. Guttmacher AE, Marchuk DA, White RI, Jr. Hereditary hemorrhagic telangiectasia. *N Engl J Med.* Oct 5 1995;333(14):918-924.

35. Flieger D, Hainke S, Fischbach W. Dramatic improvement in hereditary hemorrhagic telangiectasia after treatment with the vascular endothelial growth factor (VEGF) antagonist bevacizumab. *Ann Hematol.* Sep 2006;85(9):631-632.

36. Mitchell A, Adams LA, MacQuillan G, Tibballs J, vanden Driesen R, Delriviere L. Bevacizumab reverses need for liver transplantation in hereditary hemorrhagic telangiectasia. *Liver Transpl.* Feb 2008;14(2):210-213.

37. Dupuis-Girod S, Ginon I, Saurin JC, et al. Bevacizumab in patients with hereditary hemorrhagic telangiectasia and severe hepatic vascular malformations and high cardiac output. *JAMA : the journal of the American Medical Association.* Mar 7 2012;307(9):948-955.

38. Epperla N, Hocking W. Blessing for the Bleeder: Bevacizumab in Hereditary Hemorrhagic Telangiectasia. *Clin Med Res.* Mar 25 2014.

39. Fleagle JM, Bobba RK, Kardinal CG, Freter CE. Iron deficiency anemia related to hereditary hemorrhagic telangiectasia: response to treatment with bevacizumab. *Am J Med Sci.* Mar 2012;343(3):249-251.

40. Follner S, Ibe M, Schreiber J. Bevacizumab treatment in hereditary hemorrhagic teleangiectasia. *European journal of clinical pharmacology.* Dec 2012;68(12):1685-1686.

41. Lazaraki G, Akriviadis E, Pilpilidis I, Parisi I, Tzilves D, Tarpangos A. Low dose of bevacizumab is safe and effective in preventing bleeding episodes in hereditary hemorrhagic telangiectasia. *Am J Gastroenterol.* Dec 2011;106(12):2204-2206.

42. Lupu A, Stefanescu C, Treton X, Attar A, Corcos O, Bouhnik Y. Bevacizumab as rescue treatment for severe recurrent gastrointestinal bleeding in hereditary hemorrhagic telangiectasia. *J Clin Gastroenterol.* Mar 2013;47(3):256-257.

43. Oosting S, Nagengast W, de Vries E. More on bevacizumab in hereditary hemorrhagic telangiectasia. *N Engl J Med.* Aug 27 2009;361(9):931; author reply 931-932.

44. Suppressa P, Liso A, Sabba C. Low dose intravenous bevacizumab for the treatment of anaemia in hereditary haemorrhagic telangiectasia. *Br J Haematol.* Feb 2011;152(4):365.

45. Fodstad P, Dheyauldeen S, Rinde M, Bachmann-Harildstad G. Anti-VEGF with 3-week intervals is effective on anemia in a patient with severe hereditary hemorrhagic telangiectasia. *Ann Hematol.* May 2011;90(5):611-612.

46. Brinkerhoff BT, Choong NW, Treisman JS, Poetker DM. Intravenous and topical intranasal bevacizumab (Avastin) in hereditary hemorrhagic telangiectasia. *Am J Otolaryngol.* May-Jun 2012;33(3):349-351.

47. Iyer VN, Apala DR, Pannu BS, et al. Intravenous Bevacizumab for Refractory Hereditary Hemorrhagic Telangiectasia-Related Epistaxis and Gastrointestinal Bleeding. *Mayo Clin Proc.* Feb 2018;93(2):155-166.

48. Kim YH, Kim MJ, Choe SW, Sprecher D, Lee YJ, S PO. Selective effects of oral antiangiogenic tyrosine kinase inhibitors on an animal model of hereditary hemorrhagic telangiectasia. *J Thromb Haemost.* Jun 2017;15(6):1095-1102.

49. Kovacs-Sipos E, Holzmann D, Scherer T, Soyka MB. Nintedanib as a novel treatment option in hereditary haemorrhagic telangiectasia. *BMJ case reports.* Jun 26 2017;2017.

50. Faughnan ME, Gossage JR, Chakinala MM, et al. Pazopanib may reduce bleeding in hereditary hemorrhagic telangiectasia. *Angiogenesis.* Sep 6 2018.

51. Parambil JG, Woodard TD, Koc ON. Pazopanib effective for bevacizumab-unresponsive epistaxis in hereditary hemorrhagic telangiectasia. *Laryngoscope.* Feb 16 2018.

52. Merlo CA, Yin LX, Hoag JB, Mitchell SE, Reh DD. The effects of epistaxis on health-related quality of life in patients with hereditary hemorrhagic telangiectasia. *International forum of allergy & rhinology.* Nov 2014;4(11):921-925.

53. Cottin V. The safety and tolerability of nintedanib in the treatment of idiopathic pulmonary fibrosis. *Expert Opin Drug Saf.* Jul 2017;16(7):857-865.

54. Noth I, Oelberg D, Kaul M, Conoscenti CS, Raghu G. Safety and tolerability of nintedanib in patients with idiopathic pulmonary fibrosis in the USA. *Eur Respir J.* Jul 2018;52(1).

55. Richeldi L, Cottin V, du Bois RM, et al. Nintedanib in patients with idiopathic pulmonary fibrosis: Combined evidence from the TOMORROW and INPULSIS((R)) trials. *Respiratory medicine.* Apr 2016;113:74-79.

56. Richeldi L. Treatments for idiopathic pulmonary fibrosis. *N Engl J Med.* Aug 21 2014;371(8):783.

57. Richeldi L, du Bois RM, Raghu G, et al. Efficacy and safety of nintedanib in idiopathic pulmonary fibrosis. *N Engl J Med.* May 29 2014;370(22):2071-2082.

58. Hughes G, Toellner H, Morris H, Leonard C, Chaudhuri N. Real World Experiences: Pirfenidone and Nintedanib are Effective and Well Tolerated Treatments for Idiopathic Pulmonary Fibrosis. *Journal of clinical medicine.* Sep 2 2016;5(9).

59. Yang L, Chen CJ, Guo XL, et al. Bevacizumab and risk of intracranial hemorrhage in patients with brain metastases: a meta-analysis. *J Neurooncol.* Mar 2018;137(1):49-56.

LIST OF ANNEXES

Appendix 1: Nosebleed monitoring grid
Appendix 2: The Epistaxis Severity Score (ESS)
Appendix 3: Medical Outcomes Study 36-item Short Form (SF36)

Appendix 1: Nosebleed monitoring grid

FICHE DE SURVEILLANCE DES EPISTAXIS DANS LA MALADIE DE RENDU-OSLER

Nom :
Prénom : Né(e) le :

ANNEE : 20
MOIS :

Inscrire dans la colonne correspondant au jour du mois, **la durée en minutes** de chaque épistaxis.

Jour	1	2	3	4	5	6	7	8	9	10	11	12	13	14	15	16	17	18	19	20	21	22	23	24	25	26	27	28	29	30	31
Episode 1																															
Episode 2																															
Episode 3																															
Episode 4																															
Episode 5																															
Episode 6																															
Episode 7																															
Episode 8																															
Total																															

PRISE DU TRAITEMENT — FAIRE UNE CROIX TOUS LES JOURS POUR CHAQUE PRISE matin et soir

Jour	1	2	3	4	5	6	7	8	9	10	11	12	13	14	15	16	17	18	19	20	21	22	23	24	25	26	27	28	29	30	31
Matin																															
Soir																															

Jour	1	2	3	4	5	6	7	8	9	10	11	12	13	14	15	16	17	18	19	20	21	22	23	24	25	26	27	28	29	30	31
Transfusion (Nb CGR)																															
Perfusion Fer (mg)																															
Hémoglobine (g/L)																															
Ferritine (µg/L)																															

Noter la liste des évènements que vous jugez importants survenus pendant cette période, la date de début et de fin, la prise d'autres traitements :

Date :	Commentaire :

Appendix 2: The Epistaxis Severity Score (ESS)

EPICURE Visite __ Date : _ _ / _ _ / _ _ Id patient : _ _ - _ _ _

Score de Sévérité (ESS) des épistaxis dans la maladie de Rendu Osler

Le but de ces questions est de calculer un score de sévérité des épistaxis (saignements de nez) pour les patients atteints de maladie de Rendu-Osler et participant à une étude de recherche. Pour plus d'informations sur la signification de ce score, veuillez en discuter avec votre médecin.

Veuillez répondre à chacune des questions suivantes qui concernent vos saignements de nez **AU COURS DE CES 8 DERNIERES SEMAINES**. Merci de bien vouloir répondre à toutes les questions posées.

1. A quelle fréquence avez-vous eu des saignements de nez au cours des 2 derniers mois, **D'UNE MANIERE GENERALE ?**

- ☐ Moins d'une fois par mois
- ☐ Une à trois fois par mois
- ☐ Une fois par semaine
- ☐ Plusieurs fois par semaine
- ☐ Une fois par jour
- ☐ Plusieurs fois par jour

2. Combien de temps a duré chaque saignement de nez dans votre cas au cours des 2 derniers mois, **D'UNE MANIERE GENERALE ?**

- ☐ < 1 minute
- ☐ 1 à 5 minutes
- ☐ 6 à 15 minutes
- ☐ 16 à 30 minutes
- ☐ > 30 minutes

3. Comment décririez-vous l'intensité de vos saignements de nez au cours des 2 derniers mois, **D'UNE MANIERE GENERALE ?**

- ☐ Généralement pas abondants
- ☐ Généralement abondants

4. Avez-vous demandé un avis médical pour vos saignements de nez au cours des 2 dernier mois ?

- ☐ Non
- ☐ Oui

5. Êtes-vous actuellement anémié(e) (diminution des globules rouges) ?

- ☐ Non
- ☐ Oui

6. Avez-vous reçu une transfusion de globules rouges SPÉCIFIQUEMENT pour vos saignements de nez au cours des 2 derniers mois ?

- ☐ Non
- ☐ Oui

Appendix 3: Medical Outcomes Study 36-item Short Form (SF36) – 3 pages

Date : |__|__|/|__|__|/|__|__| Identification : |__|__|-|__|__|__| EPICURE - Visite : V |__|

QUESTIONNAIRE QUALITE DE VIE SF-36 v2

1.- En général, diriez-vous que votre santé est :

Excellente	Très bonne	Bonne	Passable	Mauvaise
☐1	☐2	☐3	☐4	☐5

2. –__Par comparaison à l'an dernier__, comment évaluez-vous, maintenant, votre santé générale ?

Bien meilleure maintenant que l'an dernier	Un peu meilleure maintenant que l'an dernier	À peu près la même que l'an dernier	Un peu moins bonne maintenant que l'an dernier	Bien moins bonne maintenant que l'an dernier
☐1	☐2	☐3	☐4	☐5

3-Les questions suivantes portent sur les activités que vous pourriez avoir à faire au cours d'une journée normale. __Votre état de santé actuel vous limite-t-il dans ces activités__ ? Si oui, dans quelle mesure ?

	Mon état de santé me limite beaucoup	Mon état de santé me limite un peu	Mon état de santé ne me limite pas du tout
a. Dans les activités exigeant un effort physique important comme courir, soulever des objets lourds, pratiquer des sports violents			
b. Dans les activités modérées comme déplacer une table, passer l'aspirateur, jouer aux quilles ou au golf			
c. Pour soulever ou transporter des sacs d'épicerie			
d. Pour monter plusieurs étages à pied			
e. Pour monter un seul étage à pied			
f. Pour me pencher, me mettre à genoux ou m'accroupir			
g. Pour faire plus d'un kilomètre à pied			
h. Pour faire plus de deux cents mètres à pied			
i. Pour faire cent mètres à pied			
j. Pour prendre un bain ou m'habiller			

4.-Au cours des quatre dernières semaines, combien de fois avez-vous eu l'une ou l'autre des difficultés suivantes au travail ou dans vos autres activités quotidiennes à cause de votre état de santé physique ?

	Tout le temps	La plupart du temps	Parfois	Rarement	Jamais
a. Avez-vous dû consacrer moins de temps à votre travail ou d'autres activités ?					
b. Avez-vous accompli moins de choses que vous l'auriez voulu ?					
c. Avez-vous été limité(e) dans la nature de vos tâches ou de vos autres activités ?					
d. Avez-vous eu de la difficulté à accomplir votre travail ou vos autres activités (par exemple vous a-t-il fallu fournir un effort supplémentaire) ?					

5.-Au cours des quatre dernières semaines, combien de fois avez-vous eu l'une ou l'autre des difficultés suivantes au travail ou dans vos autres activités quotidiennes à cause de l'état de votre moral (comme le fait de vous sentir déprimé(e) ou anxieux(se)) ?

	Tout le temps	La plupart du temps	Parfois	Rarement	Jamais
a. Avez-vous dû consacrer moins de temps à votre travail ou d'autres activités ?					
b. Avez-vous accompli moins de choses que vous l'auriez voulu ?					
c. Avez-vous fait votre travail ou vos autres activités avec moins de soin qu'à l'habitude ?					

6.-Au cours des quatre dernières semaines, dans quelle mesure votre état physique ou moral a- t-il nui à vos activités sociales habituelles (famille, amis, voisins ou autre groupe) ?

☐ Pas du tout ☐ un petit peu ☐ moyennement ☐ beaucoup ☐ énormément

7.- Au cours des quatre dernières semaines, avez-vous éprouvé des douleurs physiques?

Aucune douleur	Douleurs très légères	Douleurs légères	Douleurs moyennes	Douleurs intenses	Douleurs très intenses
▼	▼	▼	▼	▼	▼
☐1	☐2	☐3	☐4	☐5	☐6

8.- Au cours des quatre dernières semaines, dans quelle mesure la douleur a-t-elle nui à vos activités habituelles (au travail comme à la maison) ?

☐ Pas du tout ☐ un petit peu ☐ moyennement ☐ beaucoup ☐ énormément

9.- Ces questions portent sur les quatre dernières semaines. Pour chacune des questions suivantes, donnez la réponse qui s'approche le plus de la façon dont vous vous êtes senti(e). Au cours des quatre dernières semaines combien de fois :

	Tout le temps	La plupart du temps	Parfois	Rarement	Jamais
a. vous êtes-vous senti(e) plein(e) d'entrain ?					
b. Avez-vous été très nerveux (se) ?					
c. vous êtes-vous senti(e) si déprimée que rien ne pouvait vous remonter le moral ?					
d. vous êtes-vous senti(e) calme et serein(e) ?					
e. Avez-vous eu beaucoup d'énergie?					
f. vous êtes-vous senti(e) triste et démoralisé(e) ?					
g. vous êtes-vous senti(e) épuisé(e) et vidé(e) ?					
h. vous êtes-vous senti(e) heureux (se) ?					
i. vous êtes-vous senti(e) fatigué(e) ?					

10.-Au cours des quatre dernières semaines, combien de fois votre état physique ou moral a-t-il nui à vos activités sociales (comme visiter des amis, des parents, etc.) ?

☐ Tout le temps ☐ La plupart du temps ☐ parfois ☐ rarement ☐ jamais

11.-Dans quelle mesure chacun des énoncés suivants est-il VRAI ou FAUX dans votre cas ?

	Tout à fait vraie	Plutôt vraie	Ne sais pas	Plutôt faux	Tout à fait faux
a. il me semble que je tombe malade un peu plus facilement que les autres					
b. Je suis en aussi bonne santé que les gens que je connais					
c. je m'attends à ce que ma santé se détériore					
d. Ma santé est excellente					

MERCI DE VERIFIER QUE VOUS AVEZ BIEN FOURNI UNE REPONSE POUR CHACUNE DES QUESTIONS.
NOUS VOUS REMERCIONS DE VOTRE COLLABORATION.

HCL HOSPICES CIVILS DE LYON — LA RECHERCHE	Formulaire<br>**Page de signature du protocole de recherche**

Titre complet de l'étude : EFFICACY OF NINTEDANIB PER OS AS A TREATMENT FOR EPISTAXIS IN HHT DISEASE

Référence HCL : 69HCL19_0003

N° EUDRACT: 2019-002593-31

Version du protocole n°4 du 24/03/2022

	Nom et Fonction	Date	Signature
Promoteur<br>Hospices Civils de Lyon<br>3, quai des Célestins<br>69002 LYON	Thierry HEREMBERT,<br>Responsable secteur Promotion Interne de la Direction de la Recherche en Santé	12 avril 2022 | 15:54 CEST	DocuSigned by:<br>Thierry HEREMBERT<br>887AA87EDA4F4A4...
Investigateur Coordonnateur	**Docteur Sophie DUPUIS-GIROD**<br>RPPS : 10001448744<br>Unité de Génétique Clinique UF 34216<br>Centre de Référence des Maladies Rares UF 34217<br>Hôpital Femme Mère Enfant	12/04/2022	[signature]

Ce document devra être signé de nouveau suite aux éventuelles modifications du protocole.

EPICURE : Amendement 1

Efficacy of Nintedanib per os as a treatment for epistaxis in HHT disease
A national, randomized, multicenter phase II study
EPICURE

N° d'enregistrement : 2019-002593-31

Modifications apportées (Texte en gras sur fond coloré)

Modifications	Ancienne version Protocole<br>Version 2 du 16/10/2019<br>(Version initiale)	Nouvelle version Protocole :<br>Version 3 du 17/07/2020<br>(Amendement 1)	Justification
§ 2.5 p27	In order to conduct the study as safe as possible regarding hemorrhage risks, we decided:<br>1. To exclude patients with cerebral arteriovenous malformation (about 10% of patients). All patients will have a cerebral MRI before inclusion, if not done within 5 years.<br>2. To exclude patients with active gastro-intestinal bleeding or GI ulcers.<br>3. To exclude patients with hemoptysis or hematuria within 12 weeks prior to inclusion.<br>4. Not to exclude patients without PAVMs on a CT scan (<5 years) or patients with treated PAVMs (vascular treatment according to recommendations) or patients with small PAVM (<3mm) not	In order to conduct the study as safe as possible regarding hemorrhage risks, we decided:<br>1. To exclude patients with cerebral arteriovenous malformation (about 10% of patients). All patients will have a cerebral MRI before inclusion, if not done within 5 years.<br>2. To exclude patients with active gastro-intestinal bleeding or GI ulcers within 12 months prior to inclusion.<br>3. To exclude patients with hemoptysis or hematuria within 12 weeks prior to inclusion.<br>4. Not to exclude patients without PAVMs on a CT scan (<5 years) or patients with treated PAVMs (vascular treatment according to recommendations) or	Addition of details concerning the history of gastrointestinal ulcers

	accessible to a vascular treatment on CT scan within 1 year.<br>5. Not to exclude patients with hepatic AVM. The presence of telangiectasia or hepatic AVM is frequent (>80%) and in more than 80% of cases patients are asymptomatic; only 5 to 10% of patients have a severe liver involvement leading to a high-output cardiac failure, but without hemorrhagic risk.	patients with small PAVM (<3mm) not accessible to a vascular treatment on CT scan within 1 year.<br>5. Not to exclude patients with hepatic AVM. The presence of telangiectasia or hepatic AVM is frequent (>80%) and in more than 80% of cases patients are asymptomatic; only 5 to 10% of patients have a severe liver involvement leading to a high-output cardiac failure, but without hemorrhagic risk.	
§ 4.2 p28	The list of randomization will be pre-established with neutral code (1 and 2), by the Pôle de Santé publique at the Hospices Civils de Lyon – Clinical Research Unit. The pharmacy will assign the treatment to the neutral code.	The list of randomization will be pre-established with neutral code (1 and 2), by the Pôle de Santé publique at the Hospices Civils de Lyon – Clinical Research Unit. ~~**The pharmacy will assign the treatment to the neutral code.**~~	Assignation is made automatically by the platform
§ 5.2 p31	**Non-inclusion criteria**<br>• Patients with active gastro-intestinal (GI) bleeding or GI ulcers<br>• Known inherited predisposition to thrombosis or thrombotic events (including stroke and transient ischemic attack) within 12 months prior to inclusion.	**Non-inclusion criteria**<br>• Patients with active gastro-intestinal (GI) bleeding or GI ulcers **within 12 months prior to inclusion**<br>• Known inherited predisposition to thrombosis or thrombotic events (including stroke and transient ischemic attack, **excluded superficial venous thrombosis**) within 12 months prior to inclusion.	Change of non-inclusion criteria: addition of details concerning the history of gastrointestinal ulcers, and thrombosis events

§ 7.2 p36

Day (D) - Week (W)	D-84 to D-56 (=W-12 to W-8)	D0
Visit	V0 Inclusion	V1 Randomisation
Information	X	X
Informed Consent	X	
inclusion/exclusion criteria	X	X
Randomization		X
Dispensing treatments		X
Treatment (Day 1 to day 84)		
Dose adjustment		
Compliance		
Blood transfusions collection	X	X
Iron injection collection	X	X
Concomitants treatments	X	X
AE & SAE collection		X
Epistaxis grids (2)		
ENT examination		X
Clinical examination	X	X
Blood pressure / heart rate	X	X
Pregnancy Test (βHCG dosage)	X	X
SF36 QoL questionnaire		X
ESS questionnaire	X	X
Biology (NFS, hepatic function, complete iono, fe	X	X

* delivery of two urine pregnancy tests for monthly check (16w & 20w)

Table 2: synopsis of the study per patient

Day (D) - Week (W)	D-84 to D-56 (=W-12 to W-8)	D0
Visit	Standard follow-up	V1 Inclusion Randomisation
Information	X	X
Informed Consent		X
inclusion/exclusion criteria	X	X
Randomization		X
Dispensing treatments		X
Treatment (Day 1 to day 84)		
Dose adjustment		
Compliance		
Blood transfusions collection		X
Iron injection collection		X
Concomitants treatments	X	X
AE & SAE collection		X
Epistaxis grids (2)		
ENT examination		X
Clinical examination		X
Blood pressure / heart rate		X
Pregnancy Test (βHCG dosage)		X
SF36 QoL questionnaire		X
ESS questionnaire		X
Biology (NFS, hepatic function, complete iono, ferritin)		X

* delivery of two urine pregnancy tests for monthly check (16w & 20w)

Table 2: synopsis of the study per patient

Correction of the table 2 which doesn't match with the conduct of the study described in § 7.3

§ 7.3 p37

Inclusion visit/Randomization (day 0)

- The specific prescription sheet will be filled in for drug dispensation (150 mg capsules) by the pharmacist at the hospital. A sufficient amount will be dispensed for 2 weeks of treatments (+1 day), i.e. 30 capsules (1 bottle). A leaflet will be given to patient with the drug; it includes precautions for use and recommendations in case of adverse events. It will be clear to the patient that

Inclusion visit/Randomization (day 0)

- The specific prescription sheet will be filled in for drug dispensation (150 mg capsules) by the pharmacist at the hospital. A sufficient amount will be dispensed for 2 weeks of treatments (+1 day), i.e. 30 capsules (1 bottle). A leaflet will be given to patient with the drug; it includes precautions for use and recommendations in case of adverse events. It will be clear to the patient that

Addition of lines on the epistaxis grids to monitor medication intake at each visits.

	she/he should keep all the packages and bring them back to the next visit. The treatment will be start by the patient the next morning at day 1.<br>• A clinical trial participation card will be given to the patient. It will include phone numbers if needed and in case of emergency.<br>• A calendar will be given to the patient with dates for next visits.<br><br>*Follow-up visits*<br>- **Visit at 2 weeks, V2 (D14 +/- 1 day)**<br>• Collection of the nosebleed grids filled in by the patients during the first 2 weeks of treatment (Appendix 1: Nosebleed monitoring grid), if not filled in on line. These grids include monitoring of studied treatment administration.[…]<br>• The investigator will connect to Ennov's platform and new treatment number will be allocated. The specific prescription sheet will be filled in for drug dispensation	she/he should keep all the packages and bring them back to the next visit. The treatment will be start by the patient the next morning at day 1. **The patient must tick each drug intake in the table provided for this purpose on the epistaxis grids.**<br>• A clinical trial participation card will be given to the patient. It will include phone numbers if needed and in case of emergency.<br>• A calendar will be given to the patient with dates for next visits.<br>• **Epistaxis grids will be given to the patient to record all episodes of nose bleed and their duration if not completed on line.**<br><br>*Follow-up visits*<br>- **Visit at 2 weeks, V2 (D14 +/- 1 day)**<br>• Collection of the nosebleed grids filled in by the patients during the first 2 weeks of treatment (Appendix 1: Nosebleed monitoring grid), if not filled in on line. These grids include monitoring of studied treatment ~~administration~~ **intake.** […]<br>• The investigator will connect to Ennov's platform and new treatment number will be allocated. The specific prescription sheet will be filled in for drug dispensation (150 mg capsules or 100 mg capsules	

	(150 mg capsules or 100 mg capsules according to tolerance) by the pharmacist at the hospital. A sufficient amount will be dispensed for 2 weeks of treatments (+1 day), i.e. 30 capsules (1 bottle).<br><br>- **Visit at 4 and 8 weeks, V3 (D28 +/- 1 day) and V4 (D56 +/- 2 days).**<br>• Collection of the nosebleed grids filled in by the patients during the last 4 weeks of treatment (Appendix 1: Nosebleed monitoring grid), if not filled in on line. These grids include monitoring of treatment administration.<br>• The investigator will connect to Ennov's platform and new treatments numbers will be allocated. The specific prescription sheet will be filled in for drug dispensation (150 mg capsules or 100 mg capsules according to tolerance) by the pharmacist at the hospital. A sufficient amount will be dispensed for 4 weeks of treatments (+2 days), i.e. 60 capsules (2 bottles).	according to tolerance) by the pharmacist at the hospital. A sufficient amount will be dispensed for 2 weeks of treatments (+1 day), i.e. 30 capsules (1 bottle). **The patient must tick each drug intake in the table provided for this purpose on the epistaxis grids.**<br>• **Epistaxis grids will be given to the patient to record all episodes of nose bleed and their duration if not completed on line.**<br><br>- **Visit at 4 and 8 weeks, V3 (D28 +/- 1 day) and V4 (D56 +/- 2 days).**<br>• Collection of the nosebleed grids filled in by the patients during the last 4 weeks of treatment (Appendix 1: Nosebleed monitoring grid), if not filled in on line. These grids include monitoring of treatment ~~administration~~ **intake**.<br>• The investigator will connect to Ennov's platform and new treatments numbers will be allocated. The specific prescription sheet will be filled in for drug dispensation (150 mg capsules or 100 mg capsules according to tolerance) by the pharmacist at the hospital. A sufficient amount will be dispensed for 4 weeks of treatments (+2 days), i.e. 60 capsules (2 bottles). **The patient must tick each drug intake in the table provided for this purpose on the epistaxis grids.**	

	- **Visit at the end of the treatment at 12 weeks, V5 (D84 up to 94)**	• **Epistaxis grids will be given to the patient to record all episodes of nose bleed and their duration if not completed on line.**<br><br>- **Visit at the end of the treatment at 12 weeks, V5 (D84 up to 94)**<br><br>• **Epistaxis grids will be given to the patient to record all episodes of nose bleed and their duration if not completed on line.**	
§ 8.2.2	Serious adverse event reporting<br><br>The investigator faxes at +33 (0)4 72 11 51 90 a SAE form **dated and signed**, with at least these 4 points which are mandatory to submit the SAE:<br>• An investigator<br>• A subject<br>• An experimental product (if applicable)<br>• An adverse event<br><br>The patient who has experienced a SAE must be followed until complete resolution, stabilization at an acceptable threshold according to the investigator or recovery to the previous state, even if the patient has been withdrawn from the trial. The investigator has to inform the sponsor by fax on +33 (0)4 72 11 51 90 using the form (check the box: ☑ follow-up).	Serious adverse event reporting<br><br>**The investigator validates dates and sends the SAE form, by email via eCRF to drci.eig-vigilance@chu-lyon.fr, with at least these 4 criteria which are mandatory to submit the SAE:**<br>~~The investigator faxes at +33 (0)4 72 11 51 90 a SAE form **dated and signed**, with at least these 4 points which are mandatory to submit the SAE:~~<br>• An investigator<br>• A subject<br>• An experimental product (if applicable)<br>• An adverse event<br><br>The patient who has experienced a SAE must be followed until complete resolution, stabilization at an acceptable threshold according to the investigator or recovery to the previous state, even if the patient has	Change of procedure.<br>SAE declaration was included in the eCRF.

<table>
<tr>
<td></td>
<td></td>
<td>been withdrawn from the trial. ~~The investigator has to inform the sponsor by fax on +33 (0)4 72 11 51 90 using the form (check the box: ☑ follow-up).~~ **The investigator has to inform the sponsor by completing the eCRF, then email to via eCRF to drci.eig-vigilance@chu-lyon.fr (fulfill the part corresponding to follow-up (FU) with the name, the date and the signature in eCRF).**

If the eCRF is unavailable, the investigator can scan the SAE form signed and dated and send it by email to drci.eig-vigilance@chu-lyon.fr or by fax on 04 72 11 51 90. It must specify in the subject line of the email "STUDY NAME-Severity criterion * -N° patient center".

*** to be filled according to the severity criterion selected in the EIG notification form**</td>
<td></td>
</tr>
<tr>
<td>§ 8.2.7</td>
<td>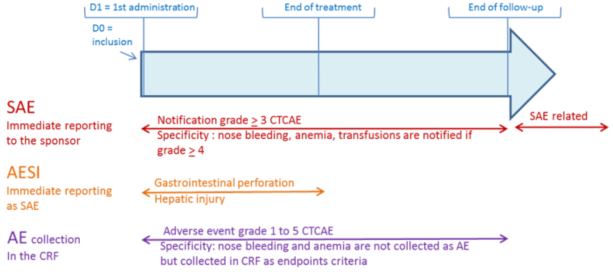

Figure 15 : Adverse Event Flow Chart</td>
<td>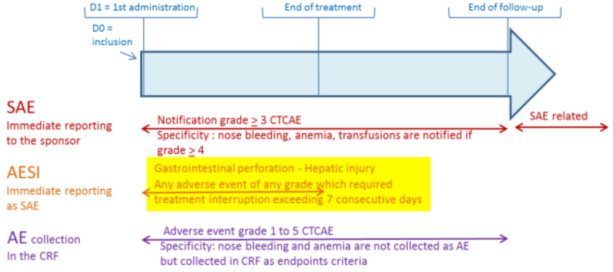

Figure 15 : Adverse Event Flow Chart</td>
<td>Flow Chart update to match the text in the protocol</td>
</tr>
<tr>
<td>8.4.2 p47</td>
<td>The expectedness or unexpectedness of a</td>
<td>The expectedness or unexpectedness of a</td>
<td>New version of IB for Ofev®,</td>
</tr>
</table>

	suspected serious adverse reaction is assessed from:<br>- Investigational Drug Reference Document n°1: Summary of Product Characteristics (SmPC) of OFEV® (Nintedanib)<br>- Investigators' brochure, doc. N° c01783972-11 version 16. Any updates, transmitted by BI, will be communicated to all investigators.	suspected serious adverse reaction is assessed from:<br>- Investigational Drug Reference Document n°1: Summary of Product Characteristics (SmPC) of OFEV® (Nintedanib)<br>- Investigators' brochure, doc. N° c01783972-15~~1~~ version 17~~6~~. Any updates, transmitted by BI, will be communicated to all investigators.	change of the RSI.
§ 9.2	***<u>Description of the statistical methods</u>***<br><br>*Study populations*<br>The intention-to-treat population is defined as all included patients having started the treatment. The per protocol population is defined as all included patient without major protocol deviation. A flow chart will present the population.	***<u>Description of the statistical methods</u>***<br><br>*Study populations*<br>The intention-to-treat population is defined as all included patients having started the treatment. The per protocol population is defined as all included patient ~~without major protocol~~ **who have received at least 80% of the total treatment, that is to say at least 134 capsules (from day 1 to day 84) and without permanent discontinuation prescribed by the investigator. In case of wrong treatment allocation: the patient will be excluded from PP population if he was in placebo group and he received nintedanib treatment; if he was in nintedanib group and received placebo, he could be included in the PP population as long as he received at least 80% of the total treatment.** A flow chart will present the population.	Addition of definition for the study population.
**Modifications**	**Ancienne version Notice d'information et consentement<br>Version 2 du 16/10/2019**	**Nouvelle version :<br>Version 3 du 17/07/2020<br>(Amendement 1)**	**Justification**

	**(Version initiale)**		
**P6** **Quels sont les bénéfices, les risques et les contraintes liés à votre participation ?**	Les effets secondaires attendus du nintedanib sont ceux qui ont été déjà observés avec ce traitement, c'est à dire pour les plus fréquemment rapportés: des troubles gastro-intestinaux : diarrhées, nausées, vomissements ; des douleurs abdominales ; une baisse d'appétit ; la perte de poids ; une augmentation du taux d'enzymes hépatiques.	Les effets secondaires attendus du nintedanib sont ceux qui ont été déjà observés avec ce traitement, c'est à dire pour les plus fréquemment rapportés: des troubles gastro-intestinaux : diarrhées, nausées, vomissements ; des douleurs abdominales ; une baisse d'appétit ; la perte de poids ; une augmentation du taux d'enzymes hépatiques. **Des maux de tête et des éruptions cutanées ont également été rapportés.**	**Following the IB update (V17), new adverse events have been added**

EPICURE : Amendement 2

Efficacy of Nintedanib per os as a treatment for epistaxis in HHT disease
A national, randomized, multicenter phase II study
EPICURE

N° d'enregistrement : 2019-002593-31

Modifications apportées (Texte en gras sur fond coloré)

Modifications	Ancienne version Protocole<br>Version 3 du 17/07/2020<br>(Amendement 1)	Nouvelle version Protocole :<br>Version 4 du 24/03/2022<br>(Amendement 2)	Justification
§ 1.6 p14	***Pharmacist***<br><br>Dr Stéphane EMERY<br>Service pharmaceutique – Secteur Essais cliniques<br>Groupement Hospitalier Est - 59, Bd Pinel - 69677 BRON<br>04 72 35 75 50<br>stephane.emery@chu-lyon.fr<br><br>Dr Caroline GERVAISE<br>Service pharmaceutique – Secteur Essais cliniques<br>Groupement Hospitalier Est<br>59, Bd Pinel<br>69677 BRON<br>04 72 35 75 50<br>caroline.gervaise@chu-lyon.fr	Dr Caroline GERVAISE<br>Service pharmaceutique – Secteur Essais cliniques<br>Groupement Hospitalier Est<br>59, Bd Pinel<br>69677 BRON<br>04 72 35 75 50<br>caroline.gervaise@chu-lyon.fr	Le Dr Stéphane Emery a quitté le service

§ 7.1 p 35	***Study calendar***<br><br>Duration of the inclusion period: 24 months<br>Length of the participation of each patient: 24 weeks, *i.e. about 6 months* (from 158 to 178 days).<br>Total duration of the study: 30 months<br>Start of inclusions: Second quarter 2019	***Study calendar***<br><br>Duration of the inclusion period: **30 months**<br>Length of the participation of each patient: 24 weeks, *i.e. about 6 months* (from 158 to 178 days).<br>Total duration of the study: **36 months**<br>Start of inclusions: **june 2020**	Le rythme des inclusions a été moins important par rapport à nos prévisions (impact de la période “COVID”)
