# Supplementary material for: Effect of oral nintedanib vs placebo on epistaxis in hereditary hemorrhagic telangiectasia: the EPICURE multicenter randomized double-blind trial
Source: Angiogenesis. 2024 Dec 24;28(1):9. doi: 10.1007/s10456-024-09962-4 (PMC11668894; doi:10.1007/s10456-024-09962-4)
Supplement: Supplementary file 3 — Supplementary file3 (PDF 1728 KB) [file 10456_2024_9962_MOESM3_ESM.pdf]

Efficacy of Nintedanib per os as a treatment for epistaxis in HHT disease
A national, randomized, multicenter phase II study

EPICURE

Statistical Analysis Plan

Study-No.: 5653

EudraCT No.: 2019-002593-31

SAP version: Final Version

Date: 13/06/2023

SAP author: Margaux HUOT

Proofreading and review: Aurélie DE MONTIGNY

Sponsor

Hospices Civils de Lyon

BP2251

3 quai des Célestins 69229 LYON Cedex 02

Coordinating and Leading Principal Investigator

Dr Sophie DUPUIS-GIROD

Department of genetics

HFME A1

59 boulevard Pinel 69677 BRON

Phone : 04 27 85 65 25, Fax : 04 27 85 67 98

Email : sophie.dupuis-girod@chu-lyon.fr

Methodologist

Dr Evelyne DECULLIER

Unité de Recherche Clinique, Pôle Santé Publique

162 avenue Lacassagne, 69424 LYON Cedex 03

Phone : 04 72 11 57 06

Email : evelyne.decullier@chu-lyon.fr

Study Coordination

Anne-Emmanuelle FARGETON

Department of genetics

HFME A1

59 boulevard Pinel 69677 BRON

Data Management Center

Adeline ROUX

Pôle Santé Publique

162 avenue Lacassagne, 69424 LYON Cedex 03

Biostatisticians

Margaux HUOT and Aurélie DE MONTIGNY

Service de Biostatistiques – Pôle Santé Publique

162 avenue Lacassagne, 69424 LYON Cedex 03

CONFIDENTIAL

Signature page

Prepared and reviewed by:

Biostatisticians

Name: Margaux HUOT Date: 13/06/2023

Name: Aurélie DE MONTIGNY Date: 13/06/2023

Approved by:

Coordinating Investigator

Name: Sophie DUPUIS-GIROD Date: 13/06/2023

DUPUIS GIROD Sophie

Study coordinator

Name: Anne-Emmanuelle FARGETON Date: 13/06/2023

AE Fargeton

Methodologist

Name: Evelyne DECULLIER Date: 14 juin 2023

References:

- Protocol : “2019-002593-31_PROTOCOLE SMA_V4_20220324_EPICURE.pdf”
- CRF : “EPICURE_CRFannoté_20221201_155126.pdf”
- Dataset : “Extraction the 2023/05/23 for the blind review”

History of previous versions		
**Version**	**Date**	**Reason for the update**
V1	13/04	First version
V2	30/05	Updates following the discussions during the meeting held on the 20April2023 for the review of the SAP
FV	13/06	Updates following the Blind Review meeting held on the 06June2023:<br>- Transfusions and infusions must be analyzed on P1, P2 and P3 (as for the primary efficacy endpoint).<br>- A test for the difference of hemoglobin level between V1/V5 and V1/V6 must be done.<br>- Imputation of missing values for the injected iron dose.<br>- Addition of a descriptive analysis of patients with iron (per os or IV) reported in the concomitant part.

Table of contents

Table of contents.....5
Abbreviations.....7
1 Introduction.....8
2 Study objectives.....8
2.1 Primary objective.....8
2.2 Secondary objectives.....8
3 Experimental design.....9
3.1 Study outline.....9
3.2 Study treatment groups.....9
3.3 Randomization and blinding.....9
3.4 Chronological sequence of visits.....10
4 Study evaluation criteria.....10
4.1 Primary efficacy endpoint.....10
4.2 Secondary efficacy endpoints.....10
4.3 Secondary safety endpoints.....11
5 Sample size determination.....11
6 Sequence of planned analyses.....12
6.1 Interim analyses.....12
6.2 Final analysis and reporting.....12
7 Study population.....12
7.1 Selection criteria for the study population.....12
7.1.1 Inclusion criteria.....12
7.1.2 Non-inclusion criteria.....12
7.2 Analysis populations.....13
7.2.1 Intention-to-treat population (ITT).....13
7.2.2 Per-protocol population (PP).....13
8 Statistical methods.....14
8.1 General issues for statistical analyses.....14
8.1.1 Statistical software.....14
8.1.2 Conventions for delay calculations.....14
8.1.3 Missing data and outliers.....14
8.1.4 Presentation of results.....14
8.1.5 Usual statistical tests.....14
8.2 General description of the study conduct.....15
8.2.1 Disposition of subjects and withdrawals.....15
8.2.2 Patients lost to follow-up.....15

8.3 Demographics and other baseline characteristics 15
8.3.1 Demographic and medical history data 15
8.3.2 Clinical data at inclusion 16
8.3.3 ENT examination at inclusion 16
8.3.4 Biological data at inclusion 17
8.3.5 Previous treatment 17
8.4 Exposition to study treatment 17
8.4.1 Study treatment discontinuation 17
8.4.2 Compliance 17
8.5 Follow-up variables 18
8.5.1 ENT examination 18
8.5.2 Clinical examination 18
8.5.3 Biological examination 19
8.5.4 Concomitant treatment 19
8.6 Efficacy analysis 20
8.6.1 General notations 20
8.6.2 Primary efficacy endpoint 20
8.6.3 Secondary efficacy endpoints 21
8.7 Safety analysis 25
9 Quality insurance 26
9.1 Data entry 26
9.2 Statistical analysis 26
10 Bibliographic references 26
11 Appendix 27

Abbreviations

**AE**	Adverse Events
**AESI**	Adverse Events with Specific Interest
**CI**	Confidence Interval
**CONSORT**	Consolidated Standards of Reporting Trials
**CT scan**	Computerized Tomography scanner
**DBP**	Diastolic Blood Pressure
**DSMB**	Data Safety Monitoring Board
**ECG**	ElectroCardioGram
**ENT**	Ear, Nose and Throat specialist
**ESS**	Epistaxis Severity Score
**GI**	Gastro-Intestinal
**HHT**	Hereditary Hemorrhagic Telangiectasia
**HLT**	High Level Term
**HR**	Heart Rate
**ITT**	Intention-To-Treat
**IWRS**	Interactive Web Response System
**MRI**	Magnetic Resonance Imaging
**PP**	Per-Protocol
**PT**	Preferred Term
**RBC**	Red Blood Cell
**SAE**	Severe Adverse Events
**SBP**	Systolic Blood Pressure
**SOC**	System Organ Class
**ULN**	Upper Limit of Normal

1 Introduction

The purpose of this detailed statistical analysis plan is to describe precisely and unambiguously the planned statistical analyses to be carried out on the EPICURE study database.

The analysis plan may be amended, in particular following the blind analysis carried out before the final database lock in preparation for the "blind review". Any statistical analysis not planned and described within this document that would be carried out after the final database lock will be considered as “post-hoc” and therefore exploratory. This will be clearly notified in the final statistical report.

The statistical analysis plan will be validated by the principal investigator, the methodologist and the coordinator prior to the final database lock and unblinding.

In this document, for more clarity if necessary, the name of the database will be mentioned into brackets as follow: [TABLE], and the name of a variable as follow: [*VARIABLE*].

2 Study objectives

The EPICURE study is a national, randomized, multicenter phase II study, evaluating the efficacy of Nintedanib per os for the treatment of epistaxis in Hereditary Hemorrhagic Telangiectasia (HHT) disease.

This study involved adult patients suffering from moderate to severe epistaxis related to HHT, which is responsible for severe alterations in social functioning and quality of life.

2.1 Primary objective

The primary objective of this study is to evaluate efficacy, at the end of the treatment period, on epistaxis duration of nintedanib treatment per os (300 mg/day for 12 weeks) *versus* placebo in HHT patients complicated by moderate to severe epistaxis.

2.2 Secondary objectives

Secondary objectives are:

1. To evaluate nintedanib safety in HHT patients.
2. To evaluate efficacy of nintedanib treatment on epistaxis (duration, frequency and severity).
3. To evaluate efficacy of nintedanib on other clinical criteria: Quality of life (SF36), number of red blood cell transfusions and number of iron infusions.
4. To evaluate efficacy of nintedanib treatment on biological criteria: hemoglobin and ferritin levels.

3 Experimental design

3.1 Study outline

The EPICURE study is a multicenter randomized phase II study, comparing nintedanib treatment *versus* placebo (ratio 1:1) carried out in a double blind setting.

Figure 1: General diagram of the study

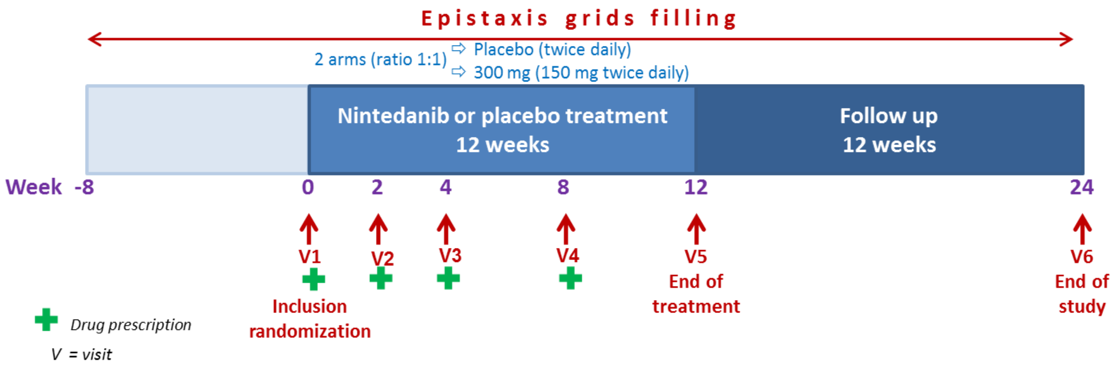

3.2 Study treatment groups

There is 2 treatment groups:

- Nintedanib group: Patients randomized in this group will receive 150mg nintedanib (capsules) twice daily administered approximately 12 hours apart.
- Placebo group: Patients randomized in this group will receive placebo as soft gelatin capsules (150mg) twice daily.

3.3 Randomization and blinding

This will be a double blind study in which neither the patient nor the investigator will be aware of the nature of the treatment administered so as to annul any bias in the follow-up and measurements. Placebo soft capsules are strictly identical as the study treatment, in order to preserve the blinding.

The randomization process will be centralized. Allocation of a randomization arm to a patient included will be made by Interactive Web Response System (IWRS) on the basis of a unique randomization list for all investigation centers. The list of randomization will be pre-established by the “Pôle de Santé Publique” at the Hospices Civils de Lyon – Clinical Research Unit.

All requests for unblinding must be clearly justified.

There is no randomization stratification factors in this study.

3.4 Chronological sequence of visits

The following table presents the chronological sequence of study visits, as set up in the protocol and reported the e-CRF, with the corresponding interval of time and variable category collected for each visit realization.

Table 1: Synopsis of the study per patient

Day (D) - Week (W)	**D-84 to D-56** *(=W-12 to W-8)*	**D0**	**D14** *(=2W) +/- 1 day*	**D28** *(=4W) +/- 1 day*	**D56** *(=8W) +/- 2 days*	**D84** *(=12W)* (D84 to D94)	**D168** *(=24W)* (D158 to D178)
Visit	**Standard follow-up**	**V1 Inclusion Randomisation**	**V2 Consultation**	**V3 Consultation**	**V4 consultation**	**V5 consultation end of treatment**	**V6 consultation end of study**
Information	X	X					
Informed Consent		X					
inclusion/exclusion criteria	X	X					
Randomization		X					
Dispensing treatments		X	X	X	X		
**Treatment (Day 1 to day 84)**		⟷	⟷	⟷	⟷		
Dose adjustment			X	X	X		
Compliance			X	X	X	X	
Blood transfusions collection		X	X	X	X	X	X
Iron injection collection		X	X	X	X	X	X
Concomitants treatments	X	X	X	X	X	X	X
AE & SAE collection		X	X	X	X	X	X
Epistaxis grids (2)	⟷	⟷	⟷	⟷	⟷	⟷	⟷
ENT examination		X				X	
Clinical examination		X	X	X	X	X	X
Blood pressure / heart rate		X	X	X	X	X	X
Pregnancy Test (βHCG dosage)		X	X	X	X	X*	X
SF36 QoL questionnaire		X				X	X
ESS questionnaire		X				X	X
Biology (NFS, hepatic function, complete iono, ferritin)		X	X	X	X	X	X

** delivery of two urine pregnancy tests for monthly check (16w & 20w)*

4 Study evaluation criteria

4.1 Primary efficacy endpoint

Proportion of participants reporting a response at the end of the treatment. A response is defined by a reduction of at least 50% on epistaxis monthly mean duration during the last 8 weeks of treatment as compared to the 8 weeks before treatment. This criterion will be assessed on the basis of monitoring of epistaxis grids filled in by the patients (collected at each visit or filled in online).

4.2 Secondary efficacy endpoints

1. **Efficacy on epistaxis:**

– Proportion of patients reporting a response at the end of follow-up. A response is defined by a reduction of at least 50% on epistaxis monthly mean duration during the last 8 weeks of follow-up as compared to the 8 weeks before treatment.

- Nosebleeds monthly mean duration will be computed during the 8 weeks before treatment, during the last 8 weeks of the treatment period and during the last 8 weeks of the follow-up period. Differences from baseline will be assessed.
- Nosebleeds monthly mean duration will be computed all over the study period using 4 weeks periods.
- Nosebleeds frequency (considered as continuous variable) will be computed during the 8 weeks before treatment, during the last 8 weeks of the treatment period and during the last 8 weeks of the follow-up. Differences from baseline will be assessed.
  NB: Assessment by epistaxis grids filled in by patients (collected at each visit or completed online).
- Epistaxis score = ESS will be calculated from questionnaire filled in by patients at inclusion visit, at the end of the treatment period and at the end of the follow-up.

2. Efficacy on other clinical criteria:

- Quality of life = SF36 score based on questionnaire filled in by patients at inclusion visit (baseline), at the end of the treatment and end of the follow up will be calculated.
- Number of red blood cell transfusions is collected for 8 weeks before treatment, during the last 8 weeks of the treatment period and during the last 8 weeks of the follow-up period.
- Number of iron infusions is collected for 8 weeks before treatment, during the last 8 weeks of the treatment period and during the last 8 weeks of the follow-up period.

3. Efficacy on biological criteria:

- Hemoglobin level will be measured at inclusion, at the end of the treatment visit and at the end of follow-up visit.
- Ferritin level will be measured at inclusion visit, at the end of the treatment visit and at the end of follow-up visit.

4.3 Secondary safety endpoints

All Adverse Events (AE) and Severe Adverse Events (SAE) observed during the study will be collected.

5 Sample size determination

The hypothesis is that 60% of patients will be improved in the treatment group against 15% in the placebo group. It is therefore necessary to include 27 patients in each group to reach a 90% power, leading to 54 patients overall.
Taking into account early withdrawal and patients who may be lost to follow-up, 30 patients will be include in each group, that is to say, a total of 60 patients.

6 Sequence of planned analyses

6.1 Interim analyses

Monitoring the safety of administration of the product, motivated by the iatrogenic risks, justifies the setting up of a specific independent monitoring and safety committee. The Data Safety Monitoring Board (DSMB) reviews the data and issues that may occur during the trial, especially the ones that are scientific, ethical and tolerance, which may change the benefit/risk ratio. Following this review, the DSMB shall provide recommendations to the sponsor, which may concern the continuation, modification or termination of the study.

During the course of this study, 4 DSMB have taken place, and at each meeting, DMSB members recommended the continuation of the study without any modification:

1. DSMB n°1 on 29/06/2021: results based on the first 10 patients included.
2. DSMB n°2 on 14/12/2021: results based on the first 39 patients included.
3. DSMB n°3 on 24/05/2022: results based on first 51 patients included;
4. DSMB n°4 on 17/01/2023: results based on all of the 60 patients included.

6.2 Final analysis and reporting

Planned analyses identified in the protocol and in this SAP will be performed only after the formal database lock. A blinded data review meeting will be held prior to database lock and completion of the final analyses. In addition, no database may be locked, random code unblinded, or analyses completed until this SAP has been approved and signed.

7 Study population

7.1 Selection criteria for the study population

7.1.1 Inclusion criteria

- Age > 18 years old
- Patients who have given their free informed and signed consent
- Patients affiliated to a social security scheme or similar
- Patients monitored for clinically confirmed HHT and/or with molecular biology confirmation
- Patient with an Epistaxis Severity Score (ESS) > 4

7.1.2 Non-inclusion criteria

- Pregnant woman or woman of child bearing potential not using two effective methods of birth control (one barrier and one highly effective non-barrier) for at least 1 month prior to trial and/or committing to using it until 3 months after the end of treatment.
- Woman who are breast-feeding.
- Patient who are protected adults under the terms of the law (French Public Health Code).
- Participation in another interventional clinical trial which may interfere with the proposed trial (judgment of the investigator).
- Clinical evidence of active infection.
- AST, ALT > 1,5 fold upper limit of normal (ULN) and/or Bilirubin > 1,5 fold upper limit of normal (ULN).

- Severe renal impairment (Creat Clearance <30 mL/min) estimated by the Cockcroft-Gault equation.
- Presence of non-treated pulmonary arteriovenous malformations accessible to a treatment on CT scan within 5 years.
- Patients with hemoptysis or hematuria within 12 weeks prior to inclusion.
- Patients with active gastro-intestinal (GI) bleeding or GI ulcers within 12 months prior to inclusion.
- Presence of cerebral arteriovenous malformation on MRI done within 5 years prior inclusion.
- Patients who require full-dose therapeutic anticoagulation (e.g. vitamin K antagonist or heparin, dabigatran) or high dose antiplatelet therapy, patients under anticoagulation with rivaroxaban, apixaban and epixaban.
- Patients with P-glycoprotein (P-gp) substrates/inducers/inhibitors (e.g.: ketoconazole, erythromycin, cyclosporine, rifampicin, carbamazepine, phenytoin, and St. John's Wort).
- Patients with known coronary artery disease or recent history of myocardial infarction (within 1 year).
- Known inherited predisposition to thrombosis or thrombotic events (including stroke and transient ischemic attack, excluded superficial venous thrombosis) within 12 months prior to inclusion.
- Patients with QTc prolongation (on ECG, less than 3 months).
- Hypersensitivity to nintedanib, peanut or soya, or to any of the excipients.
- Patient who incompletely filled in epistaxis grids within 8 weeks prior to inclusion.
- Patient who have received intravenous bevacizumab within 6 months prior to inclusion.
- Patient who had surgery (including ENT surgery) within 12 weeks prior to inclusion.
- Unhealed wound.
- Planned major surgery within the next 3 months, including liver transplantation, major abdominal or intestinal surgery.

7.2 Analysis populations

A patient included in the study has been randomized twice, with two ID patient (0827 and 0829) as well as two randomization codes (W008 and I072). The patient with ID 0827 has been firstly randomized under the randomization code W008 in July 2020, but at this time he couldn't start the treatment yet; so he came back in September 2020 where he was randomized a second time under the code I072 (with ID 0829). Both randomization codes are in the same treatment group.
For the analysis, patient with ID 0827 (with only few data at baseline) will be excluded, and the second one with ID 0829 kept.

7.2.1 Intention-to-treat population (ITT)

The intention-to-treat population is defined as all included patients having started the treatment, according to their arm randomly allocated.
Description of patients at baseline (inclusion) will be done in ITT. Primary and secondary efficacy endpoints will be analyzed in ITT as well as safety endpoints.

7.2.2 Per-protocol population (PP)

The per-protocol population is defined as all included patients with a good compliance, *i.e.* receiving at least 80% of the total treatment, and without treatment permanent discontinuation.
The 4 following patients will be excluded from the PP population for the following reasons:

- Patient 0135, with a bad compliance of 60%, due to one temporary discontinuation of treatment of 33 days.
- Patient 0541, with a bad compliance of 77%, partially due to one temporary discontinuation of treatment of 14 days.
- Patient 0325, with a permanent discontinuation of treatment due to a SAE.
- Patient 0829, with a permanent discontinuation of treatment due to a SAE.

A sensitivity analysis of the primary endpoint will be performed on the per-protocol population.

8 Statistical methods

Statistical analyses will be performed by the biostatistics unit of the Hospices Civils de Lyon.

8.1 General issues for statistical analyses

8.1.1 Statistical software

All statistical analyses will be performed using SAS® Entreprise Guide Software version 7.1 in a Windows environment or R software version 4.1.1.

8.1.2 Conventions for delay calculations

Calculated delays since randomization will be calculated as the time elapsed since the day of randomization (D0).

Delays will be converted in years or months by dividing the delays by 365.25 days or 30.4375 days respectively.

The epistaxis grids will be filled before the inclusion (D0). To name, for example, the day 10 before inclusion, we note day -10.

8.1.3 Missing data and outliers

All efforts will be made to collect outcome data also in patients withdrawn from the trial for whichever reasons and to minimize the amount of missing data.

Unless specifically indicated, missing data will not be replaced. Handling of missing data for the epistaxis data will be detailed in sections 8.6.2 and 8.6.3.1.

8.1.4 Presentation of results

Description of continuous variables includes the number of patients with available data, mean, standard deviation, median, 1st and 3rd quartile, and range. Categories can be defined using cut-off threshold from literature or according to quantiles.

Description of categorical variables includes the number of patients with available data, frequencies and percentages (missing data will not be counted for percentages calculation).

8.1.5 Usual statistical tests

Unless otherwise specified, between-group differences will be tested using the Mann-Whitney test for quantitative outcomes and using the Fisher test for qualitative outcomes (both non-parametric tests).

A two-sided p-value of less than 0.05 will be considered to indicate statistical significance. Two-sided 95% confidence intervals will be used.

8.2 General description of the study conduct

8.2.1 Disposition of subjects and withdrawals

Number of randomized patients

Number of randomized patients who completed the study in each treatment group

Number of randomized patients who completed the treatment period in each treatment group

The frequency and percent of subjects in each population (ITT, per-protocol) will also be presented by treatment arm.

Number of randomized patients with a low compliance (<80%) and listing of patients with reason.

Duration of follow-up (days): from randomization date to end of study date (variable [*RFENDSTC*] from [ES]).

8.2.2 Patients lost to follow-up

NA, No patients were lost to follow-up.

8.3 Demographics and other baseline characteristics

Patient characteristics at baseline will be described overall and by treatment group on the ITT population. No statistical tests will be performed according to the CONSORT recommendations (Moher D, Hopewell S, Schulz KF, Montori V, Gøtzsche PC, Devereaux PJ, et al. CONSORT 2010 Explanation and Elaboration: Updated guidelines for reporting parallel group randomised trials. J Clin Epidemiol. 2010; 2010(63):e1–37).

8.3.1 Demographic and medical history data

Demography

- Age (years)
- Sex (Male / Female)
- Weight (kg)
- Height (cm)

- Gene involved in the disease:
    - ALK1
    - ENG
    - SMAD4
    - Not identified

Medical history

- ENT (Ear, Nose, Throat : Otolaryngologist) history: surgery and cauterization (laser and chemical) (Y/N)
- Others surgical history (Y/N)
- Pulmonary history (Y/N)
    - If yes, then:
        - Presence of AVM (Arterio-Venous Malformation) accessible to a procedure (Y/N)

    - Hemoptysis (Y/N)
- Digestive signs within the last 12 months (Y/N)
  - If yes, then:
    - Active digestive bleedings (Y/N)
    - Gastro-intestinal ulcers (Y/N)
- Cerebral history (Y/N)
  - If yes, then presence of AVM (Y/N)
- Hematuria history (Y/N)
- Others medical history (Y/N)

A listing of ENT history details will be provided.

8.3.2 Clinical data at inclusion

ESS

- ESS score at inclusion

For the computation of the score and handling of missing data, see appendix 1: "ESS score computation".

Clinical examination

- Systolic blood pressure SBP (mmHg)
- Diastolic blood pressure DBP (mmHg)
- Heart rate HR (bpm)

ECG

- Presence of QT long syndrome in the ECG (Y/N)
- Abnormality detected in the ECG (Y/N)

8.3.3 ENT examination at inclusion

- Septal perforation (Y/N)
- Dysosmia (Y/N)
- Nasal obstruction (Y/N)
- Nasal humidification within the 8 previous weeks (Y/N)
  - If yes, then:
    - Frequency (Daily / Occasional)
    - Product used
- Lesions (Y/N)
- Right nasal fossa crust (Y/N)
  - If yes, then number of crusts (<50%/>50% of the visible area)
- Left nasal fossa crust (Y/N)
  - If yes, then number of crusts (<50%/>50% of the visible area)

8.3.4 Biological data at inclusion

- Hemoglobin (g/L)
- Ferritin (µg/L)
- ALAT (U/L)
- ASAT (U/L)
- Bilirubin (µmol/L)
- Gamma-GT (U/L)

8.3.5 Previous treatment

- Blood transfusion within the last 8 weeks (Y/N), with details of previous transfusion
- Iron infusions within the last 8 weeks (Y/N), with details of previous iron perfusion
- Bevacizumab treatment (Y/N), with date of last perfusion

Note: The modalities "CARBOXYMALTOSE FERRIQUE" and "FERINJECT" of the variable [*PERF_FER*] from [PERFUSION] were grouped under the modality "FERINJECT".

8.4 Exposition to study treatment

8.4.1 Study treatment discontinuation

Number and percentage of patients from the ITT population will be provided for:

- Patients with a dose reduction ([*DIMINUTION_YN*] from table [PRESCRIPTION_TTT])
- Patients with at least one temporary treatment interruption ([*ARRET_TEMPYN*] from table [PRESCRIPTION_TTT] or variable [*AEACN*] from table [EI_SOC]). Patients with an interruption strictly inferior to 7 days will be reported separately from those with an interruption up to or equal to 7 days.
- Patients with a definitely treatment interruption ([*ARRET_DEFYN*] from table [PRESCRIPTION_TTT])

All patients from the ITT population having a reduced dose, a temporary or definitely treatment interruption during the study will be listed with the reason(s).

8.4.2 Compliance

Calculation of the compliance is based on information reported by investigator at each post baseline visit on the e-CRF:

- The number of missing capsules is obtained from the variable [*ARRET_NBMANQ*] from the table [VISITES].
- If the patient stopped the treatment definitively, the variable [*ARRET_DEFYN*] from the table [PRESCRIPTION_TTT] contains "Oui".

The following formulas will be applied:

- If the patient didn't stopped the treatment before the end of the treatment period:
    - $Compliance = \frac{168 - number\ of\ missing\ capsules}{168} \times 100$

  considering that taking the overall treatment means taking theoretically 168 capsules in total.

- If the patient has definitively stopped the treatment before the end of the treatment period:
  - the date of end of the treatment is given by the variable [*ARRET_DELDTC*] from the table [PRESCRIPTION_TTT].
  - the theoretical total number of capsules on this period is computed considering this previous date as the final one. Considering N_d as the number of day elapsed from the randomization day to the end of treatment day (included): $Total\ number\ of\ capsules = 2 \times N_d$
  - $Compliance = \frac{Total\ number\ of\ capsules - number\ of\ missing\ capsules}{Total\ number\ of\ capsules} \times 100$

A patient is defined with a good compliance if he has received at least 80% of the total treatment.

The compliance will be described as continuous variable as well as categorical variable considering the 80% threshold.

8.5 Follow-up variables

Descriptive analyses of follow-up variables will be based on the Intention-to-treat population.

8.5.1 ENT examination

Descriptive analyses of the following ENT parameters will be provided overall and by treatment group at V5 visit:

- Septal perforation (Y/N)
- Dysosmia (Y/N)
- Nasal obstruction (Y/N)
- Nasal humidification within the 8 previous weeks (Y/N)
  - If yes, then:
    - Frequency (Daily / Occasional)
    - Product used
- Lesions (Y/N)
- Right nasal fossa crust (Y/N)
  - If yes, then number of crusts (<50%/>50% of the visible area)
- Left nasal fossa crust (Y/N)
  - If yes, then number of crusts (<50%/>50% of the visible area)

8.5.2 Clinical examination

Descriptive analyses of the following clinical parameters will be provided overall and by treatment group at each follow-up visit (V2, V3, V4, V5 and V6):

- SBP (mmHg)
- DBP (mmHg)
- HR (bpm)

8.5.3 Biological examination

Descriptive analyses of the following biological parameters will be provided overall and by treatment group at each follow-up visit (V2, V3, V4, V5 and V6):

- ALAT (U/L)
- ASAT (U/L)
- Bilirubin (µmol/L)
- Gamma-GT (U/L)

8.5.4 Concomitant treatment

Number of patients with at least one iron *per os*.

Number of patients with at least one iron IV.

A listing of those patients with the details of corresponding concomitant treatments could be provided.

Note: Information relative to concomitant treatment are collected in the table [CM].

- The following treatments will be considered as iron *per os* (variable [*CM_TERM*] in the following list and [*CM_ROUTE*]=’per os’ or ‘po’ or ‘oral’ or ‘orale’):

**CM_TERM**
ASCOFER
BISGLYCINATE DE FER
FER
FER + VITAMINE C
FER CHELATE
FER VEGETAL
FERO-GRAD
FERO-GRAD VITAMINE C
FERO GRAD VITAMINE C
FERO GRADE
FEROGRAD
FEROGRAD VIT C
FERROGRAD
FUMAFER
TARDIFERON
TARDYFERON
TARDYFERON B9
TIMOFEROL
TIMOFEROL 50
TRADYFERON

- The following treatments will be considered as iron IV (variable [*CM_TERM*] in the following list and [*CM_ROUTE*]=’IV’):

**CM_TERM**
FERINJECT
VENOFER

8.6 Efficacy analysis

Statistical analyses of the primary and secondary efficacy endpoints will primarily be based on the Intention-to-treat population. Secondarily, the same analyses will be repeated on the Per-protocol population for the primary efficacy endpoint.

8.6.1 General notations

Patients have filled epistaxis grids every day from week -8 until week 24 of the study. These grids contains the number of episodes per day and their duration, as well as the daily treatment intake (morning and evening). The figure bellow illustrate the epistaxis periods of collection and the corresponding analyzed periods.

Figure 2: Epistaxis reporting and corresponding analyzed periods for main endpoints

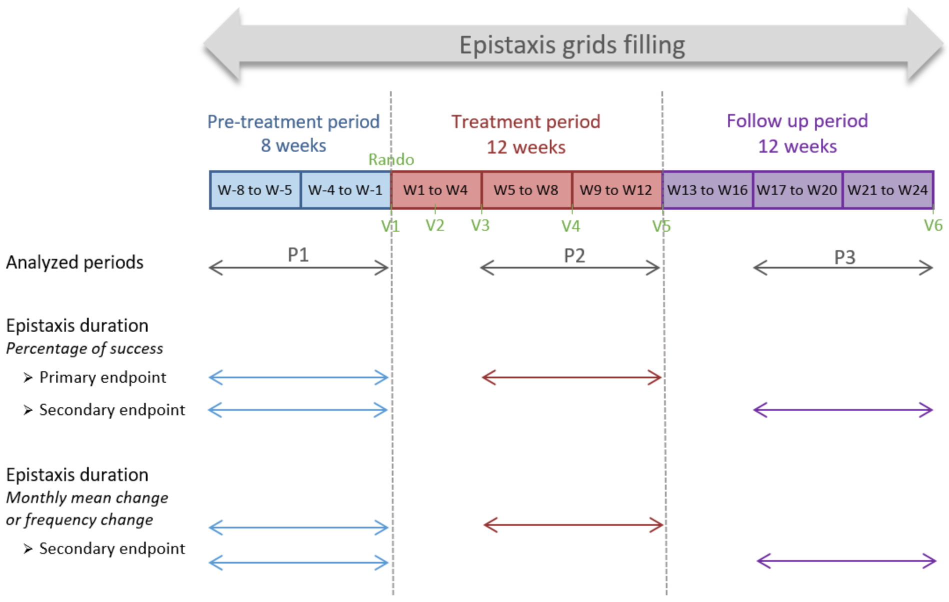

The three analyzed periods of 8 weeks (noted P1, P2, and P3 on the figure) will respectively be called the pre-treatment period, the treatment period and the follow-up period. To construct these periods, the variable [*EP_SEM*] from the table [EPISTAXIS] was used, containing the week's number of the study (value between -8 and 24):

- Pre-treatment period (P1) : [*EP_SEM*] in the interval [-8 ; -1]
- Treatment period (P2) : [*EP_SEM*] in the interval [5 ; 12]
- Follow-up period (P3) : [*EP_SEM*] in the interval [17 ; 24]

8.6.2 Primary efficacy endpoint

8.6.2.1 Construction of the primary efficacy endpoint

The primary efficacy endpoint is the proportion of patients experiencing improvement in their nosebleeds, *i.e.* a reduction of at least 50% on epistaxis monthly mean duration during the last 8 weeks of treatment as compared to the 8 weeks before treatment (on the basis of monitoring of epistaxis grids filled in by the patients).

The epistaxis monthly mean durations on the pre-treatment period (P1) and the treatment period (P2) are computed thanks to the variable [*EP_TOTAL*] from the table [EPISTAXIS], containing the epistaxis total duration per day.

The epistaxis monthly mean duration on each reporting period is computed over 56 theoretical days or less (corresponding to 8 weeks) and normalized/reduced to 28 days, considering that 1 month is equal to 4 weeks, thanks to the following formula:

$$28 * \frac{\sum_{1}^{\max 56} epistaxis\ daily\ duration}{total\ number\ of\ days\ (observed)}$$

Where $total\ number\ of\ days\ (observed)$ is the number of day with [*EP_TOTAL*] not being missing.

To obtain the primary efficacy criterion, the epistaxis monthly mean durations calculated on the periods P1 and P2 are compared:

- If the monthly mean duration of P2 is inferior or equal to 50% of the monthly mean of P1 → **success**
- If not → **failure**

The variable containing the result is [*rep_ttt_incl*].

Handling of missing data for the epistaxis data on P1 and P2 periods:

For patients with few missing data (less than 14 days), the monthly mean duration will be computed from the data available (from the 8 weeks, 56 days, period evaluated). No imputation will be performed.

8.6.2.2 Statistical analysis

The proportion of patients in the two groups will be compared using a Fisher test (or Chi2 test if the hypotheses of this test are fulfilled) on the ITT population. The difference will be tested with a 5% significance test.
To evaluate the robustness of the results, the same analysis will be performed on the PP population.

A secondary analysis on the primary endpoint will be done by estimating the difference of proportion between groups (and corresponding 95% CI).
An estimation of the OR (and corresponding 95% CI) could also be provided through a logistic model adjusted on the treatment group and on the monthly mean duration of epistaxis on P1.
A boxplot of the monthly mean duration by period and treatment group will also be provided.

8.6.3 Secondary efficacy endpoints

All secondary efficacy endpoints analyses will be performed on the ITT population.

Two more formulas are used, one to calculate the absolute change between two periods and the other one to calculate the relative change between two periods.

Absolute change:

$$Absolute\ change = new\ value - value\ of\ reference$$

Relative change:

$$Relative\ change = \frac{new\ value - value\ of\ reference}{value\ of\ reference}$$

8.6.3.1 Epistaxis

For the following endpoints, handling of missing data on P3 will be as followed:

- Patients with more than 14 days (included) missing over the whole P3 period, epistaxis monthly mean duration and monthly frequency will be considered as missing.
- For the endpoint relative to the treatment response , the following rules will be applied:
  - If less than 14 days (included) are missing: the monthly mean duration used to evaluate the result will be computed from the data available (from the 8 weeks, 56 days, period evaluated),
  - If more than 14 days missing on grids, the result for the concerned patient will be considered as a failure if the patient is in the nintedanib group or considered as a success if he is in the placebo group.

- Proportion of patients reporting a response to the treatment, comparing:
  - the pre-treatment period (P1) with the follow-up period (P3),
  - and the treatment period (P2) with the follow-up period (P3)

  This response will be computed as for the primary endpoint on P1, P2 and P3.

- Absolute and relative change (mean and 95% CI) of the epistaxis monthly mean duration
  - between P1 and P2,
  - between P1 and P3,
  - between P2 and P3

  For patients with more than 14 days (included) missing over P3 period, endpoints will be considered as missing.

- Absolute and relative change (mean and 95% CI) of epistaxis monthly frequency
  - between P1 and P2,
  - between P1 and P3,
  - between P2 and P3

  For patients with more than 14 days (included) missing over P3 period, endpoints will be considered as missing.

  The monthly frequency of epistaxis episodes on reporting period is calculated as follow: $28 * \frac{number\ of\ nosebleeds}{number\ of\ day\ (observed)}$.

  With the number of nosebleeds obtained by summing per day the number of variables with a non-zero value among the following variables from the table [EPISTAXIS]: [*EP1_VAL*], [*EP2_VAL*], [*EP3_VAL*], [*EP4_VAL*], [*EP5_VAL*], [*EP6_VAL*], [*EP7_VAL*], [*EP8_VAL*], [*EP9_VAL*].

- Mean duration of epistaxis all over the study period using a 4-week gathering **(*)**.

- Absolute change (mean and 95% CI) of ESS score *(see appendix 1 : "ESS score computation" for the construction of the score)*
  - ➢ between V1 and V5,
  - ➢ between V1 and V6,
  - ➢ between V5 and V6

For these secondary efficacy endpoints relative to the epistaxis, the following analysis will be performed:

- Fisher test for the proportions of patients reporting a response,
- Mann-Whitney test to compare between groups the absolute and relative changes of monthly mean duration and epistaxis monthly frequency,
- Mann-Whitney test to compare between groups the absolute change of ESS score,
- A graphic to show the evolution according to time and treatment of epistaxis for the criterion **(*)**.

8.6.3.2 Quality of life

The SF36 questionnaire gives 8 sub-scores that can be summarized into 2 summary scores: the Physical Health Score and the Mental Health Score. The 8 sub-scores are the result of the mean of questions spread out between the sub-scores as follow:

1. Physical Functioning: items 3a to 3j
2. Role-Physical: items 4a to 4d
3. Bodily Pain: items 7 and 8
4. General Health: items 1 and 11a to 11d
5. Vitality: items 9a, 9e, 9g and 9i
6. Social Functioning: items 6 and 10
7. Role-Emotional: items 5a to 5c
8. Mental Health: items 9b to 9d, 9f and 9h

For the construction of the 8-subscores and the 2 summary scores and also for the handling of missing data, see Appendix 2: "SF36 v2 score computation".

Relative change (mean and 95% CI) of SF36 sub-scores and summary scores, with V1 as reference in both cases:

- ➢ between V1 and V5, and
- ➢ between V1 and V6.

The sub-scores (as T-scores) will be described overall and by treatment group with usual statistics.

The 2 summary scores (as T-scores) are analyzed with a Mann-Whitney test to compare the relative changes between groups.

A spider graph, according to treatment group, representing each sub-scores (T-scores) at v1, V5 and V6, will also be presented.

8.6.3.3 Other clinical criteria

The other clinical criteria are:

- Absolute change of the cumulated number of Red Blood Cell (RBC) transfused
  - ➢ between P1 and P2, and
  - ➢ between P1 and P3.

- Absolute change of the <u>cumulated dose of injected iron (mg)</u>
  - ➢ between P1 and P2, and
  - ➢ between P1 and P3.

Those clinical criteria are collected on the pre-treatment period (8 weeks), the whole treatment period (12 weeks from week 1 to week 12), and the whole follow-up period (12 weeks from week 13 to week 24).

For the analysis, only RBC transfusion and iron perfusion done on the P1 (all pre-treatment period), P2 (restricted to the last 8 weeks of the period, so week 5 to week 12) and P3 (restricted to the last 8 weeks of the period, so week 17 to week 24) periods are of interest.

Note: To determine these analyzed periods, the period and the date of both transfusion and infusion ("S-8 à J0" for the pre-treatment period, "J1 à J84" for the whole treatment period and "J85 à J168" for the whole follow-up period), containing respectively in the variables [*TRANSFU_DTC*] and [*TRANSFU_MOMENT*] from [TRANSFUSION] and variables [*PERF_DTC*] and [*PERFU_MOMENT*] from [PERFUSION], are used. The dates of visits V1 (start of treatment period) and V5 (start of follow-up period), contained in [*VISIT_DTC*] from [VISITES], are also useful.

- If [*TRANSFU_MOMENT*] (resp. [*PERFU_MOMENT*]) = "S-8 à J0" (P1), the transfusion (resp. infusion) was done during the P1 period; all transfusion (resp. infusion) are considered.
- If [*TRANSFU_MOMENT*] (resp. [*PERFU_MOMENT*]) = "J1 à J84" (P2), the delay between date of V1 and date of transfusion (resp. infusion) is computed:
  - ➢ If this delay is strictly up to 28 days, the transfusion (resp. infusion) was done during the P2 period.
  - ➢ Otherwise, the transfusion (resp. infusion) was not considered as done during the P2 period.
- If [*TRANSFU_MOMENT*] (resp. [*PERFU_MOMENT*]) = "J85 à J168" (P3), the delay between date of V5 and date of transfusion (resp. infusion) is computed:
  - ➢ If this delay is strictly up to 28 days, the transfusion (resp. infusion) was done during the P3 period.
  - ➢ Otherwise, the transfusion (resp. infusion) was not considered as done during the P3 period.

<u>Handling of incomplete data for infusion date:</u>

Only one patient (0541) has an incomplete date for an infusion date, which will be imputed to the 15th of the month.

<u>Handling of missing data for iron injected dose:</u>

Two patients were reviewed during the blind review with the principal investigator; considering the previous and next infusions for each patient (on each period), the following missing iron injected doses were imputed:

- Patient 0545, infusion n° 3: iron injected dose imputed to 1000mg (same value as the other infusions received before).
- Patient 0721, infusions n° 1 to 6: iron injected dose imputed to 250mg (same value as the following infusions received).

The number of RBC for each transfusion and the injected iron dose for each infusion are collected in the variable [*TRANSFU_CGR*] from table [TRANSFU] and variable [*PERF_DOS*] from table [PERFUSION] respectively. The cumulated number of RBC and cumulated dose of injected iron are obtained by summing each variable on each separated period P1, P2 and P3. For patients without any transfusion (resp. infusion), the cumulated number of RBC (resp. cumulated dose of injected iron) will be imputed to 0.

For the transfusion endpoint, a descriptive analysis will be provided overall and by treatment group.

For the infusion endpoint, a Mann-Whitney test will be used to compare absolute changes between groups. A boxplot of the cumulated dose could also be provided according to treatment group for each period.

8.6.3.4 Biological criteria

- Relative change of hemoglobin level, with V1 as reference in both case
  - between V1 and V5, and
  - between V1 and V6.
- Relative change of ferritin level, with V1 as reference in both case
  - between V1 and V5, and
  - between V1 and V6.

For the ferritin level analysis, patients who received at least one iron IV during the study will be excluded.

A Mann-Whitney test to compare between groups the relative change of hemoglobin level will be used.
Descriptive analyses will be provided overall and by treatment group for the ferritin level.

8.7 Safety analysis

Safety parameters will be assessed on the ITT Population.
All AE will be coded by the Pharmacovigilance department (from the Direction de la Recherche en Santé) using the MedDRA dictionary (version 25.1) with their Preferred Term (PT) and System Organ Class (SOC) term.

An adverse event assessment committee was set-up to evaluate the relationship between the AE and nintedanib. In the following analyses, the corresponding variable [*C3*] from table [EI_SOC_CVE] will be used to describe the AE related to treatment.

The following events will be described overall and by treatment group, by SOC and PT (from table [EI_SOC_CVE]):

- All AE
- Serious AE
- AE of Special interest (AESI) (variable [*EIP*])
- AE related to treatment (variable [*C3*])
- AE leading to study drug withdrawal, to dose reduction

The following analysis will be provided for each type of events, overall and by treatment group:

- Descriptive table by SOC/PT, with number of events and number of patients with at least one event (and corresponding percentages)
- Listing of all events with details

For all AE only:

- Number and percentage of events by intensity (according to CTCAE classification)
- Number and percentage of events according to the relationship to treatment
- Descriptive statistics of number of AEs per patient

No statistical tests will be performed.

9 Quality insurance

9.1 Data entry

Data entry and data quality controls will be described in the Data management Plan document.

9.2 Statistical analysis

The derivation of the primary endpoint will be independently performed by a second biostatistician from the raw data, and compared with the results of the trial statistician.

10 Bibliographic references

1. The HHT-ESS was developed by Jeffrey B. Hoag, M.D., M.S. from Drexel University College of Medicine in conjunction with Christian A. Merlo, M.D., M.P.H. and members of The Johns Hopkins University HHT Center of Excellence. See on website : https://apps.med.drexel.edu/hht-ess

2. HAWTHORNE, Graeme, OSBORNE, Richard H., TAYLOR, Anne, et al. The SF36 Version 2: critical analyses of population weights, scoring algorithms and population norms. Quality of Life Research, 2007, vol. 16, p. 661-673.

3. Moher D, Hopewell S, Schulz KF, Montori V, Gøtzsche PC, Devereaux PJ, et al. CONSORT 2010 Explanation and Elaboration: Updated guidelines for reporting parallel group randomised trials. J Clin Epidemiol. 2010;2010(63):e1–37

11 Appendix

Appendix 1: ESS score computation

All information needed to Epistaxis Severity Score computation is contained in the table [ESS].

1. Coding answers:

A lowest value indicates a better health status.

- Question 1 [*ESS_VAL1*]:
  - 0 : Moins d'une fois par semaine
  - 1 : Une à trois fois par semaine
  - 2 : Une fois par semaine
  - 3 : Plusieurs fois par semaine
  - 4 : Une fois par jour
  - 5 : Plusieurs fois par jour
- Question 2 [*ESS_VAL2*]:
  - 0 : < 1 minute
  - 1 : 1 à 5 minutes
  - 2 : 6 à 15 minutes
  - 3 : 16 à 30 minutes
  - 4 : > 30 minutes
- Question 3 [*ESS_VAL3*]:
  - 0 : Généralement pas abondants
  - 1 : Généralement abondants
- Questions 4, 5 et 6 [*ESS_VAL4*], [*ESS_VAL5*], [*ESS_VAL6*]:
  - 0 : Non
  - 1 : Oui

2. Score computation:

$$\frac{[ESS_{VAL1}] \times 0.14 + [ESS_{VAL2}] \times 0.25 + [ESS_{VAL3}] \times 0.25 + [ESS_{VAL4}] \times 0.30 + [ESS_{VAL5}] \times 0.20 + [ESS_{VAL6}] \times 0.31}{2.76} \times 10$$

where 2.76 is the value of the numerator if all the questions have the maximum value.

Missing values:

The 5th question ask the patient if he is anemic, which he may not know. Thus if this answer is missing, since we have at each visit for the value of the hemoglobin, we can replace the missing value based on the hemoglobin value measured at the same visit.

Appendix 2: SF36 v2 score computation

Table of main differences between SF36 v1 and v2:

Questions	Version 1	Version 2
		Simpler instructions and questionnaire items.<br>An improved layout for questions and answers.
4a to 4d and 5a to 5c<br>(correspond to sub-scales RP, RE)	Dichotomous answers:<br>Oui/Non	Answers with 5 degrees:<br>Tout le temps, La plupart du temps, Parfois, Rarement, Jamais
9a to 9i and 10<br>(correspond to sub-scales VT, MH, SF)	Answers with 6 degrees:<br>En permanence, Très souvent, Souvent, Quelquefois, Rarement, Jamais	Answers with 5 degrees:<br>Tout le temps, La plupart du temps, Parfois, Rarement, Jamais

1. Coding answers:

A biggest value indicates a better health status.

Each question is normalized to be between 0-100. For each question the lowest value is 0, the biggest value is 100, and depending on the number of answer's degrees, coding answers is either:

- 3 degrees of answer: 0, 50, 100
- 5 degrees of answer: 0, 25, 50, 75, 100
- 6 answer's degrees: 0, 20, 40, 60, 80, 100

2. Construction of sub-scores:

Each question participate at the construction of a unique sub-score. The question number 2 is excluded from any computation of score.

A sub-score is the result of the mean of some items, following the allocation hereafter:

1. Physical Functioning (PF): items 3a to 3j
2. Role-Physical (RP): items 4a to 4d
3. Bodily Pain (BP): items 7 and 8
4. General Health (GH): items 1 and 11a to 11d
5. Vitality (VT): items 9a, 9e, 9g and 9i
6. Social Functioning (SF): items 6 and 10
7. Role-Emotional (RE): items 5a to 5c
8. Mental Health (MH): items 9b to 9d, 9f and 9h

Missing data: In each sub-score:

- If 50% or more of the items are missing, the sub-score is set to missing.
- If strictly less than 50% of the items are missing, we compute the mean only for items available.

3. z-score transformation:

Each sub-score is normalized with its own mean and standard deviation (sd). The following coefficients correspond to the US population for both version 1 and 2 of the SF36.

	Version 1		**Version 2**	
	Mean	Sd	Mean	Sd
PF	84.52	22.89	83.29	23.76
RP	81.20	33.80	82.51	25.52
BP	75.49	23.56	71.33	23.66
GH	72.21	20.17	70.85	20.98
VT	61.05	20.87	58.31	20.02
SF	83.60	22.38	84.30	22.92
RE	81.29	33.03	87.40	21.44
MH	74.84	18.01	74.99	17.76

4. **Construction of 2 summary scores:** Physical Health Score (PHS) and Mental Health Score (MHS)

Each sub-score participate in the computation of each summary score by multiplying the sub-score by a correlation coefficient, following the table hereafter (values for the US population and version V2 of the SF36 which are identical to V1).

	PHS	MHS
PF	0.424	-0.230
RP	0.351	-0.123
BP	0.318	-0.097
GH	0.250	-0.016
VT	0.029	0.235
SF	-0.008	0.269
RE	-0.192	0.434
MH	-0.221	0.486

Missing data: If only 1 sub-score is missing, the 2 summary scores are set to missing too.

5. T-score transformation:

Each sub-score and the 2 summary scores are transformed into T-score that is to say considering the same standardized scoring with a mean of 50 and a standard deviation of 10.

$$T\text{-}score = 50 + z\text{-}score * 10$$
