# Supplementary material for: Effect of oral nintedanib vs placebo on epistaxis in hereditary hemorrhagic telangiectasia: the EPICURE multicenter randomized double-blind trial
Source: Angiogenesis. 2024 Dec 24;28(1):9. doi: 10.1007/s10456-024-09962-4 (PMC11668894; doi:10.1007/s10456-024-09962-4)
Supplement: Supplementary file 4 — Supplementary file4 (PDF 1059 KB) [file 10456_2024_9962_MOESM4_ESM.pdf]

SF36 survey

8 sub-scores

Table 32: Descriptive analysis of SF36 8 sub-scores (T-scores) at V1, V5 and V6 - ITT population

**Visit**	**V1**<br>N = 60	**V5**<br>N = 60	**V6**<br>N = 60
Physical Functioning (PF)			
Treatment arm=Nintedanib			
Mean (SD)	46.36 (7.07)	46.26 (8.90)	47.55 (6.93)
Median	47.56	47.56	47.56
Q1-Q3	40.20 - 52.30	38.62 - 52.82	44.41 - 52.82
Min-Max	33.88 - 57.03	25.47 - 57.03	31.31 - 57.03
N	30	30	30
Treatment arm=Placebo			
Mean (SD)	46.09 (9.55)	48.55 (7.35)	45.28 (11.24)
Median	48.62	50.72	49.67
Q1-Q3	38.62 - 54.40	46.51 - 52.82	35.99 - 52.82
Min-Max	17.05 - 57.03	27.57 - 57.03	21.26 - 57.03
N	30	29	28
Role-Physical (RP)			
Treatment arm=Nintedanib			
Mean (SD)	38.43 (9.66)	41.91 (10.88)	42.81 (9.90)
Median	37.26	42.16	40.93
Q1-Q3	34.81 - 42.16	32.36 - 49.51	37.26 - 51.96
Min-Max	20.12 - 56.85	17.67 - 56.85	27.46 - 56.85
N	30	29	30
Treatment arm=Placebo			
Mean (SD)	41.83 (9.34)	44.27 (9.23)	41.71 (10.35)
Median	42.16	44.61	42.16
Q1-Q3	37.26 - 47.06	37.26 - 51.96	36.04 - 49.51
Min-Max	20.12 - 56.85	25.02 - 56.85	17.67 - 56.85
N	30	29	27
Bodily Pain (BP)			
Treatment arm=Nintedanib			
Mean (SD)	49.54 (10.46)	50.32 (10.39)	51.59 (10.32)
Median	48.38	50.49	52.61
Q1-Q3	43.10 - 61.06	40.19 - 62.12	41.78 - 62.12
Min-Max	25.14 - 62.12	29.36 - 62.12	33.59 - 62.12
N	30	30	30

Visit	V1<br>N = 60	V5<br>N = 60	V6<br>N = 60
Treatment arm=Placebo			
Mean (SD)	50.88 (11.23)	51.26 (8.94)	49.40 (10.89)
Median	57.36	52.61	47.85
Q1-Q3	43.10 - 62.12	44.15 - 57.89	44.15 - 62.12
Min-Max	29.36 - 62.12	29.36 - 62.12	24.08 - 62.12
N	30	29	28
General Health (GH)			
Treatment arm=Nintedanib			
Mean (SD)	40.06 (8.33)	41.21 (10.10)	40.72 (10.19)
Median	37.68	42.45	40.06
Q1-Q3	35.30 - 46.62	35.30 - 49.59	32.91 - 44.83
Min-Max	25.76 - 56.74	18.61 - 56.74	18.61 - 59.13
N	30	29	29
Treatment arm=Placebo			
Mean (SD)	40.28 (9.92)	41.97 (9.98)	40.36 (9.93)
Median	36.49	40.06	40.06
Q1-Q3	32.91 - 49.45	32.91 - 49.59	32.32 - 46.32
Min-Max	25.76 - 59.13	25.76 - 59.13	25.76 - 61.51
N	30	29	28
Vitality (VT)			
Treatment arm=Nintedanib			
Mean (SD)	45.95 (7.63)	48.65 (10.02)	48.76 (9.55)
Median	45.85	52.09	48.97
Q1-Q3	39.61 - 48.97	42.73 - 55.21	39.61 - 58.34
Min-Max	33.36 - 64.58	27.12 - 64.58	36.48 - 64.58
N	30	29	29
Treatment arm=Placebo			
Mean (SD)	47.72 (7.85)	51.55 (9.70)	48.53 (11.22)
Median	50.53	52.09	47.93
Q1-Q3	43.51 - 52.09	45.85 - 58.34	41.17 - 58.34
Min-Max	27.12 - 61.46	30.24 - 67.70	30.24 - 70.82
N	30	29	28
Social Functioning (SF)			
Treatment arm=Nintedanib			
Mean (SD)	41.03 (11.93)	43.58 (12.13)	45.40 (11.22)
Median	40.49	45.94	45.94
Q1-Q3	35.03 - 51.40	35.03 - 56.85	35.03 - 56.85
Min-Max	13.22 - 56.85	24.13 - 56.85	24.13 - 56.85
N	30	30	30

Visit	V1<br>N = 60	V5<br>N = 60	V6<br>N = 60
Treatment arm=Placebo			
Mean (SD)	41.40 (9.93)	47.07 (10.55)	42.44 (12.35)
Median	40.49	45.94	43.22
Q1-Q3	35.03 - 45.94	45.94 - 56.85	35.03 - 52.76
Min-Max	24.13 - 56.85	24.13 - 56.85	13.22 - 56.85
N	30	29	28
Role-Emotional (RE)			
Treatment arm=Nintedanib			
Mean (SD)	40.33 (12.33)	43.14 (13.54)	43.44 (12.39)
Median	38.39	48.10	44.22
Q1-Q3	32.56 - 53.93	32.56 - 55.88	32.56 - 55.88
Min-Max	17.01 - 55.88	9.24 - 55.88	20.90 - 55.88
N	30	29	30
Treatment arm=Placebo			
Mean (SD)	43.55 (12.12)	46.49 (10.36)	43.50 (11.41)
Median	44.22	48.10	44.22
Q1-Q3	32.56 - 55.88	40.33 - 55.88	36.44 - 55.88
Min-Max	17.01 - 55.88	20.90 - 55.88	13.12 - 55.88
N	29	29	27
Mental Health (MH)			
Treatment arm=Nintedanib			
Mean (SD)	46.19 (9.50)	46.15 (10.10)	46.12 (9.29)
Median	50.01	47.19	50.01
Q1-Q3	39.45 - 52.82	41.56 - 52.82	41.56 - 52.82
Min-Max	24.67 - 64.08	21.85 - 61.27	21.85 - 55.64
N	30	29	29
Treatment arm=Placebo			
Mean (SD)	47.75 (8.44)	48.55 (8.92)	47.29 (11.53)
Median	50.01	50.01	50.01
Q1-Q3	44.38 - 52.82	44.38 - 55.64	41.56 - 55.64
Min-Max	24.67 - 61.27	30.30 - 64.08	19.04 - 64.08
N	30	29	28

Table 33: Relative change of SF36 8 sub-scores (T-scores) between V1-V5 and V1-V6 - ITT population

Treatment arm	Nintedanib<br>N = 30	Placebo<br>N = 30	p-value
Relative change of PF between V1 and V5 (//V1)			
Mean (SD)	0.01 (0.18)	0.08 (0.25)	p=0.230 (Mann-Whitney)
95% CI	[-0.06, 0.07]	[-0.01, 0.18]	
Median	0.00	0.00	
Q1-Q3	-0.08 - 0.15	-0.04 - 0.09	
Min-Max	-0.43 - 0.44	-0.19 - 1.23	
N	30	29	
Relative change of PF between V1 and V6 (//V1)			
Mean (SD)	0.04 (0.15)	0.01 (0.21)	p=0.785 (Mann-Whitney)
95% CI	[-0.02, 0.09]	[-0.07, 0.09]	
Median	0.00	0.00	
Q1-Q3	-0.07 - 0.14	-0.06 - 0.09	
Min-Max	-0.18 - 0.44	-0.56 - 0.56	
N	30	28	
Relative change of RP between V1 and V5 (//V1)			
Mean (SD)	0.10 (0.25)	0.08 (0.22)	p=0.549 (Mann-Whitney)
95% CI	[0.01, 0.20]	[-0.01, 0.16]	
Median	0.09	0.05	
Q1-Q3	-0.07 - 0.26	-0.06 - 0.12	
Min-Max	-0.36 - 0.76	-0.23 - 0.71	
N	29	29	
Relative change of RP between V1 and V6 (//V1)			
Mean (SD)	0.15 (0.27)	0.05 (0.26)	p=0.182 (Mann-Whitney)
95% CI	[0.05, 0.25]	[-0.05, 0.16]	
Median	0.06	0.00	
Q1-Q3	-0.04 - 0.34	-0.13 - 0.19	
Min-Max	-0.21 - 0.97	-0.36 - 0.71	
N	30	27	
Relative change of BP between V1 and V5 (//V1)			
Mean (SD)	0.06 (0.31)	0.04 (0.20)	p=0.605 (Mann-Whitney)
95% CI	[-0.06, 0.18]	[-0.04, 0.11]	
Median	0.00	0.00	
Q1-Q3	-0.06 - 0.21	-0.07 - 0.12	
Min-Max	-0.49 - 0.92	-0.28 - 0.57	
N	30	29	

Treatment arm	Nintedanib<br>N = 30	Placebo<br>N = 30	p-value
Relative change of BP between V1 and V6 (//V1)			
Mean (SD)	0.08 (0.34)	0.02 (0.29)	p=0.388 (Mann-Whitney)
95% CI	[-0.05, 0.21]	[-0.09, 0.13]	
Median	0.00	0.00	
Q1-Q3	-0.07 - 0.15	-0.16 - 0.08	
Min-Max	-0.29 - 1.47	-0.43 - 1.12	
N	30	28	
Relative change of GH between V1 and V5 (//V1)			
Mean (SD)	0.05 (0.23)	0.05 (0.18)	p=0.969 (Mann-Whitney)
95% CI	[-0.03, 0.14]	[-0.02, 0.12]	
Median	0.00	0.00	
Q1-Q3	-0.07 - 0.14	-0.06 - 0.17	
Min-Max	-0.40 - 0.51	-0.27 - 0.47	
N	29	29	
Relative change of GH between V1 and V6 (//V1)			
Mean (SD)	0.03 (0.25)	0.04 (0.18)	p=0.817 (Mann-Whitney)
95% CI	[-0.06, 0.13]	[-0.03, 0.11]	
Median	0.00	0.00	
Q1-Q3	-0.15 - 0.14	-0.07 - 0.10	
Min-Max	-0.34 - 0.61	-0.29 - 0.51	
N	29	28	
Relative change of VT between V1 and V5 (//V1)			
Mean (SD)	0.07 (0.22)	0.10 (0.27)	p=1.000 (Mann-Whitney)
95% CI	[-0.01, 0.16]	[-0.00, 0.20]	
Median	0.07	0.06	
Q1-Q3	-0.09 - 0.25	-0.06 - 0.20	
Min-Max	-0.38 - 0.60	-0.42 - 1.15	
N	29	29	
Relative change of VT between V1 and V6 (//V1)			
Mean (SD)	0.08 (0.20)	0.05 (0.32)	p=0.310 (Mann-Whitney)
95% CI	[0.00, 0.16]	[-0.07, 0.17]	
Median	0.06	0.00	
Q1-Q3	-0.07 - 0.14	-0.16 - 0.18	
Min-Max	-0.25 - 0.68	-0.42 - 1.27	
N	29	28	

Treatment arm	Nintedanib<br>N = 30	Placebo<br>N = 30	p-value
Relative change of SF between V1 and V5 (//V1)			
Mean (SD)	0.10 (0.27)	0.15 (0.25)	p=0.391 (Mann-Whitney)
95% CI	[0.00, 0.20]	[0.06, 0.24]	
Median	0.00	0.11	
Q1-Q3	0.00 - 0.21	0.00 - 0.40	
Min-Max	-0.31 - 0.83	-0.36 - 0.68	
N	30	29	
Relative change of SF between V1 and V6 (//V1)			
Mean (SD)	0.18 (0.43)	0.06 (0.30)	p=0.414 (Mann-Whitney)
95% CI	[0.02, 0.34]	[-0.05, 0.18]	
Median	0.11	0.00	
Q1-Q3	0.00 - 0.38	-0.11 - 0.16	
Min-Max	-0.31 - 2.06	-0.55 - 0.74	
N	30	28	
Relative change of RE between V1 and V5 (//V1)			
Mean (SD)	0.10 (0.31)	0.08 (0.23)	p=0.520 (Mann-Whitney)
95% CI	[-0.02, 0.21]	[-0.01, 0.16]	
Median	0.11	0.00	
Q1-Q3	0.00 - 0.26	-0.09 - 0.23	
Min-Max	-0.63 - 0.72	-0.26 - 0.72	
N	29	28	
Relative change of RE between V1 and V6 (//V1)			
Mean (SD)	0.12 (0.29)	0.07 (0.31)	p=0.396 (Mann-Whitney)
95% CI	[0.01, 0.23]	[-0.06, 0.19]	
Median	0.00	0.00	
Q1-Q3	0.00 - 0.26	-0.14 - 0.26	
Min-Max	-0.43 - 0.91	-0.40 - 0.74	
N	30	27	
Relative change of MH between V1 and V5 (//V1)			
Mean (SD)	0.01 (0.18)	0.02 (0.17)	p=0.875 (Mann-Whitney)
95% CI	[-0.05, 0.08]	[-0.04, 0.09]	
Median	0.00	0.00	
Q1-Q3	-0.05 - 0.06	-0.07 - 0.07	
Min-Max	-0.53 - 0.43	-0.34 - 0.46	
N	29	29	

Treatment arm	Nintedanib N = 30	Placebo N = 30	p-value
Relative change of MH between V1 and V6 (//V1)			
Mean (SD)	0.02 (0.20)	0.00 (0.19)	p=0.719 (Mann-Whitney)
95% CI	[-0.06, 0.10]	[-0.07, 0.07]	
Median	0.00	0.00	
Q1-Q3	-0.09 - 0.11	-0.10 - 0.10	
Min-Max	-0.39 - 0.51	-0.47 - 0.36	
N	29	28	

Figure 3: Spider graph of SF36 8 sub-scores (T-scores) at V1, V5 and V6 for Nintedanib group - ITT population

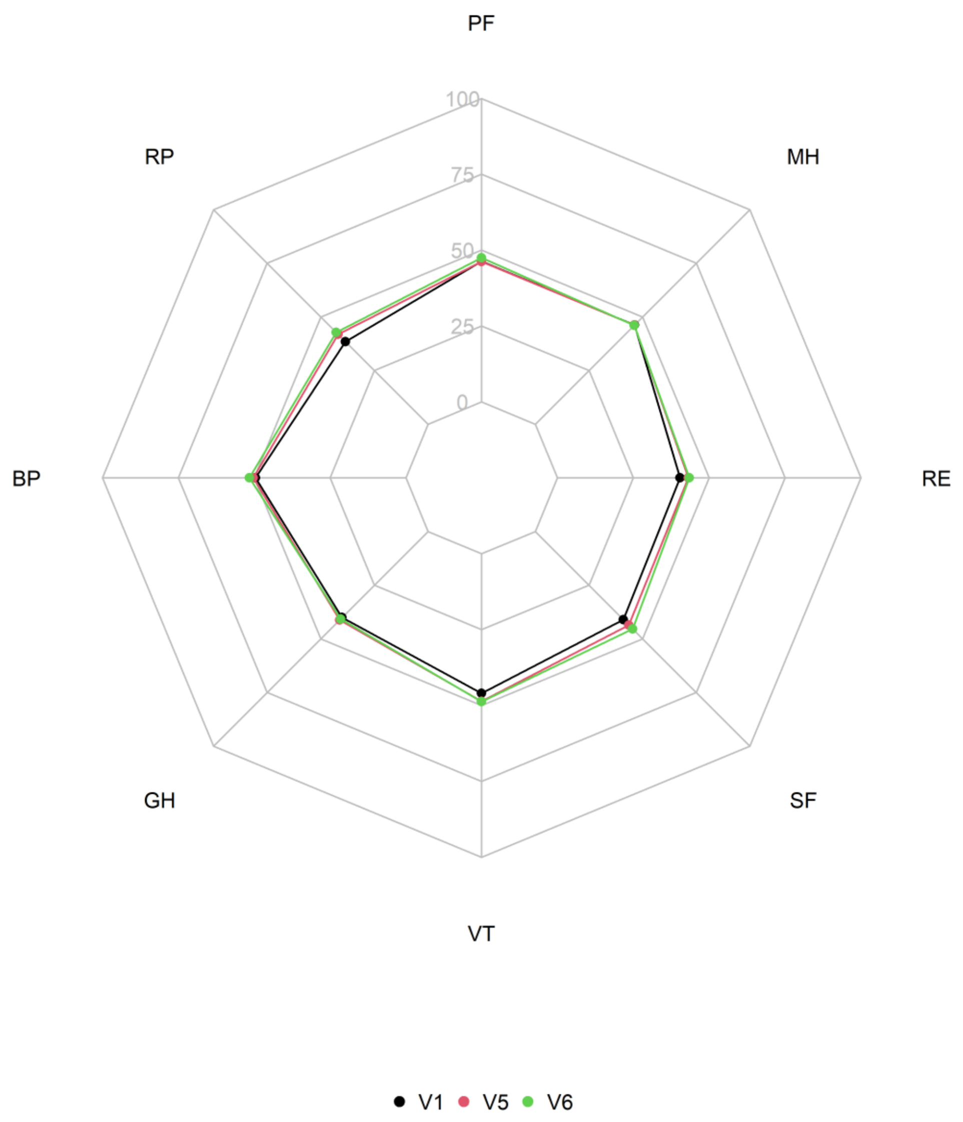

Figure 4: Spider graph of SF36 8 sub-scores (T-scores) at V1, V5 and V6 for Placebo group - ITT population

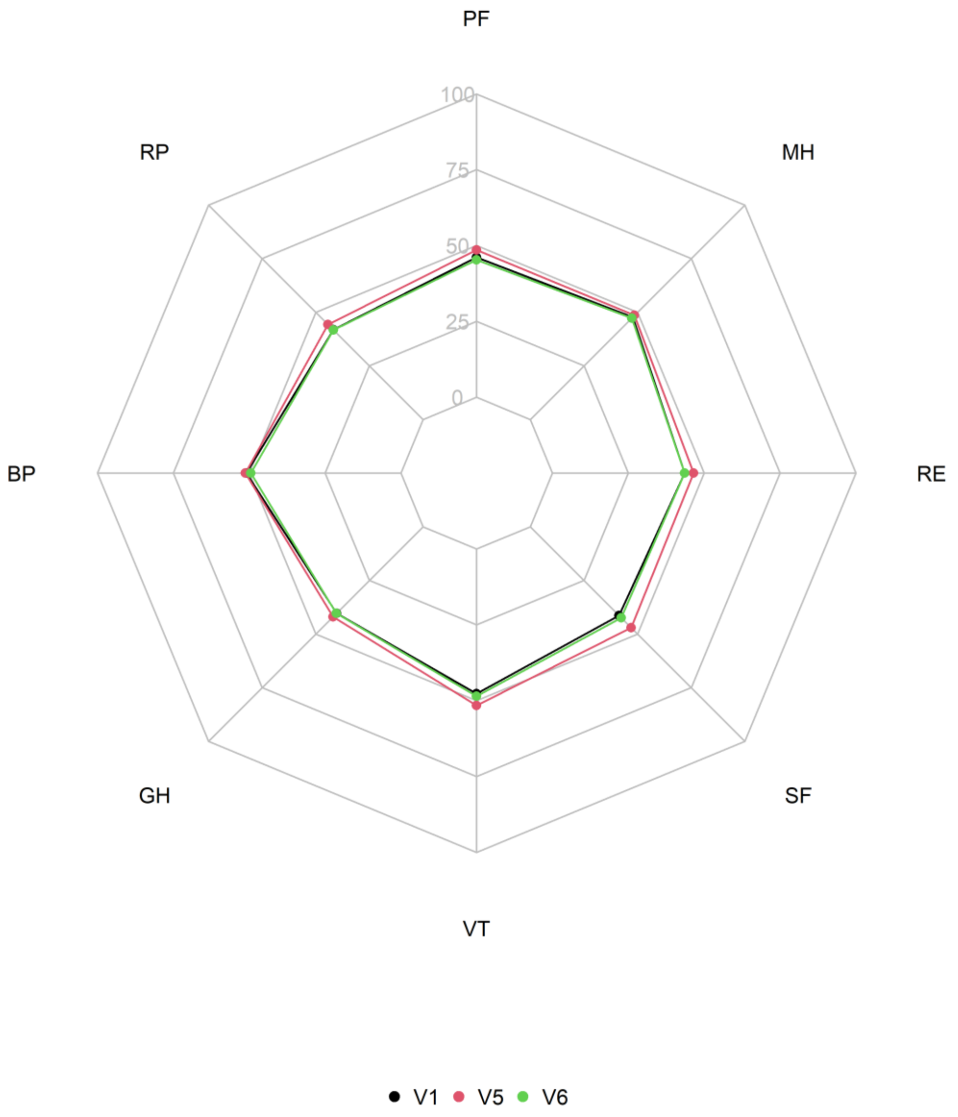

Table 34: Descriptive analysis of SF36 summary scores (T-scores) at visit V1, V5 and V6 - ITT population

**Visit**	**V1**<br>N = 60	**V5**<br>N = 60	**V6**<br>N = 60
Physical Health Score (PHS)			
Treatment arm=Nintedanib			
Mean (SD)	44.42 (7.14)	46.11 (7.85)	47.09 (6.50)
Median	43.99	45.85	46.25
Q1-Q3	40.66 - 48.75	40.83 - 52.52	43.61 - 48.68
Min-Max	30.15 - 57.04	26.01 - 57.93	37.98 - 60.00
N	30	28	29
Treatment arm=Placebo			
Mean (SD)	44.83 (8.98)	46.83 (7.93)	44.10 (8.71)
Median	45.72	46.62	43.86
Q1-Q3	38.76 - 51.29	44.07 - 50.82	39.88 - 50.51
Min-Max	26.83 - 57.81	19.18 - 59.72	16.64 - 57.68
N	29	29	27
Mental Health Score (MHS)			
Treatment arm=Nintedanib			
Mean (SD)	43.05 (10.89)	44.66 (11.88)	45.05 (11.28)
Median	43.70	49.39	48.81
Q1-Q3	38.54 - 50.96	36.01 - 54.16	33.97 - 54.88
Min-Max	14.48 - 58.99	20.59 - 60.63	22.74 - 58.54
N	30	28	29
Treatment arm=Placebo			
Mean (SD)	45.26 (9.58)	48.39 (10.10)	45.89 (12.07)
Median	48.87	50.00	46.98
Q1-Q3	39.62 - 51.77	42.00 - 55.81	38.43 - 55.33
Min-Max	19.76 - 61.65	26.90 - 64.26	18.95 - 64.71
N	29	29	27

Table 35: Relative change of SF36 summary scores (T-scores) between V1-V5 and V1-V6 - ITT population

Treatment arm	Nintedanib<br>N = 30	Placebo<br>N = 30	p-value
Relative change of PHS between V1 and V5 (//V1)			
Mean (SD)	0.04 (0.17)	0.06 (0.15)	p=0.608 (Mann-Whitney)
95% CI	[-0.03, 0.11]	[-0.00, 0.12]	
Median	0.02	0.02	
Q1-Q3	-0.09 - 0.16	-0.05 - 0.14	
Min-Max	-0.21 - 0.49	-0.29 - 0.45	
N	28	28	
Relative change of PHS between V1 and V6 (//V1)			
Mean (SD)	0.07 (0.18)	0.01 (0.15)	p=0.246 (Mann-Whitney)
95% CI	[0.00, 0.14]	[-0.05, 0.07]	
Median	0.04	-0.02	
Q1-Q3	-0.06 - 0.15	-0.05 - 0.06	
Min-Max	-0.22 - 0.56	-0.38 - 0.33	
N	29	27	
Relative change of MHS between V1 and V5 (//V1)			
Mean (SD)	0.08 (0.24)	0.07 (0.22)	p=0.643 (Mann-Whitney)
95% CI	[-0.02, 0.17]	[-0.02, 0.16]	
Median	0.10	0.04	
Q1-Q3	-0.02 - 0.17	-0.04 - 0.19	
Min-Max	-0.43 - 0.76	-0.41 - 0.81	
N	28	28	
Relative change of MHS between V1 and V6 (//V1)			
Mean (SD)	0.10 (0.35)	0.05 (0.27)	p=0.602 (Mann-Whitney)
95% CI	[-0.04, 0.23]	[-0.06, 0.16]	
Median	0.04	0.03	
Q1-Q3	-0.14 - 0.23	-0.10 - 0.17	
Min-Max	-0.41 - 1.55	-0.46 - 0.73	
N	29	27	
